# Directional Macrocycle Transport, Release, and Recapture Enabled by a Rotaxane Transporter

**DOI:** 10.1002/chem.202501106

**Published:** 2025-04-22

**Authors:** Sohom Kundu, Shubhadip Mallick, Jan Riebe, Jochen Niemeyer

**Affiliations:** ^1^ Faculty of Chemistry (Organic Chemistry) and Center for Nanointegration Duisburg‐Essen (CENIDE) University of Duisburg‐Essen Universitätsstrasse 7 45141 Essen Germany; ^2^ Research Center for Trustworthy Data Science and Security (UA Ruhr) Joseph‐von‐Fraunhofer‐Str. 25 44227 Dortmund Germany

**Keywords:** molecular devices, molecular release systems, molecular transporters, rotaxanes, supramolecular chemistry

## Abstract

A transporter for a directional macrocycle transport, release, and recapture was constructed. This was achieved using a rotaxane featuring a dibenzo‐24‐crown‐8 macrocycle, dibenzylammonium (DBA)/methyl triazolium (MTA) stations on the thread and anthracene/triisopropylsilyl‐acetylene stoppers, respectively. In the protonated rotaxane, the macrocycle primarily resides on the DBA station, followed by directional shuttling to the MTA station upon treatment with base. Addition of fluoride as an additional chemical input cleaves the triisopropylsilyl stopper, leading to release of the macrocycle and the half‐thread into solution. The released macrocycle can be recaptured by protonation, and the mechanical bond can be reestablished via CuAAC click reaction, enabled by the terminal acetylene unit on the half‐thread. This generates an elongated second‐generation rotaxane transporter, which was used for a second cycle of directional macrocycle transport and release, proving the possibility of an iterative operation of the rotaxane‐transporter in this molecular design.

## Graphical abstract

This study represents a bistable rotaxane that enables the directional shuttling, release, and recapture of a dibenzo‐24‐crown‐8 macrocycle upon addition of suitable chemical stimuli. This two‐step molecular transport and release can be followed by NMR and distinct changes in the molecular fluorescence. Based on rational molecular design, the liberated macrocycle is recaptured on to the thread and the mechanical bond is regenerated which allows for directional macrocycle transport and release in an iterative manner.

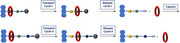

## 1 Introduction

Many biological systems such as motor proteins (e.g., kinesin, dynein),^[^ ^1^ ^]^ ion pumps,^[^ ^2^ ^]^ and molecular motors^[^ ^3^ ^]^ operate through directional and stepwise movements^[^ ^4^ ^]^ to perform life‐sustaining functions^[^ ^5^ ^]^ such as intracellular transport,^[^ ^6^ ^]^ cargo delivery,^[^ ^7^ ^]^ ion channel regulation,^[^ ^8^ ^]^ and more. Such sophisticated functions of biological systems have inspired supramolecular chemists to construct artificial molecular devices^[^ ^9^ ^]^ based on mechanically interlocked molecules (MIMs)^[^ ^10^ ^]^ to mimic biomolecular complexity and function. Among functional MIMs,^[^ ^11^ ^]^ rotaxanes have garnered significant attention stemming from their ability to exhibit large amplitude movements^[^ ^12^ ^]^ of the macrocycle along the thread.^[^ ^13^ ^]^ Enabled by suitable stations on the thread, such co‐conformational changes can be controlled by external stimuli such as pH,^[^ ^14^ ^]^ light,^[^ ^15^ ^]^ chemical stimuli,^[^ ^16^ ^]^ redox processes,^[^ ^17^ ^]^ chemical fuels,^[^ ^18^ ^]^ or even mechanical force.^[^ ^19^ ^]^ These molecular designs have been exploited to develop functional interlocked molecules such as molecular switches,^[^ ^20^ ^]^ molecular muscles,^[^ ^21^ ^]^ molecular rachets,^[^ ^22^ ^]^ processive catalysts,^[^ ^23^ ^]^ molecular therapeutics,^[^ ^11^, ^24^ ^]^ nanovalves,^[^ ^25^ ^]^ drug delivery devices,^[^ ^26^ ^]^ cargo transporters,^[^ ^27^ ^]^ ion transporters,^[^ ^28^ ^]^ and information processing devices.^[^ ^29^ ^]^

**Figure 1:**
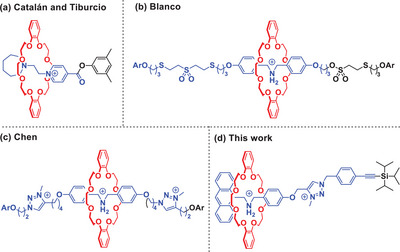
Previously reported rotaxane‐based macrocycle transporters as reported by Catalán and Tiburcio (a) Blanco (b) and Chen (c) together with this work (d). Cleavable stopper groups are shown in black.

Directional rotaxane transporters^[^ ^30^ ^]^ have recently gained significant interest in designing functional molecular machinery where co‐conformational changes of the macrocyclic component occur in a particular direction through defined states when exposed to chemical stimuli. On the other hand, with a view to creating molecular release systems^[^ ^31^ ^]^ rotaxanes with cleavable stoppers^[^ ^32^ ^]^ have been studied extensively. Here, the release of a macrocycle from the thread to the bulk solution can occur following simple dethreading mechanism.^[^ ^33^ ^]^ For example, Catalan and Tiburcio reported a macrocycle transporter where the macrocycle dissociation is enabled by transesterification (see Figure 1a).^[^ ^31c^ ^]^ Blanco and coworkers studied a [2]rotaxane enabling release of the macrocycle upon bromide‐induced cleavage of a vinyl sulfonate stopper (see Figure 1b).^[^ ^31e^ ^]^ Chen and coworkers reported a [2]rotaxane transporter which allows for unidirectional macrocycle release by DBU‐induced cleavage of a phenolate stopper (see Figure 1c).^[^ ^31a^ ^]^

However, in the reported cases, macrocycle release is an irreversible process and the mechanical bond is permanently lost. As such, these release systems can function only for singular use unlike biological analogues which are often reversible or repetitive.^34^ The fabrication of a rotaxane‐based directional release system requires rational molecular design, information handling, and interference‐free interplay among multiple stimuli and chemical entities. Along this line, here we demonstrate a stepwise and directional release of a macrocycle into the solution enabled by a stopper cleavable bistable [2]rotaxane (see Figure 1d) driven by chemical stimuli. Importantly, the released macrocycle can be recaptured and the mechanical bond can be regenerated, thus resetting the system to an interlocked state that can be reutilized in additional release/recapture cycles.

## 2 Results and Discussion

As our transport system, we designed [2]rotaxane **R1‐H^2+^** featuring a dibenzo‐24‐crown‐8 macrocycle and dibenzyl‐ammonium (DBA)/methyl triazolium (MTA)^[^ ^35^ ^]^ as stations on the thread (see Figure 1). Anthracene was chosen as the stopper appended to the DBA station for monitoring the macrocycle transport process through fluorescence read‐out. A triisopropylsilylacetylene (TIPS‐acetylene) group was chosen as the second stopper for two reasons: The TIPS group can readily be cleaved in the presence of a fluoride source, and this leads to formation of a terminal alkyne, which is amenable for further modification, for example, by an alkyne‐azide click reaction.

**Figure 2:**
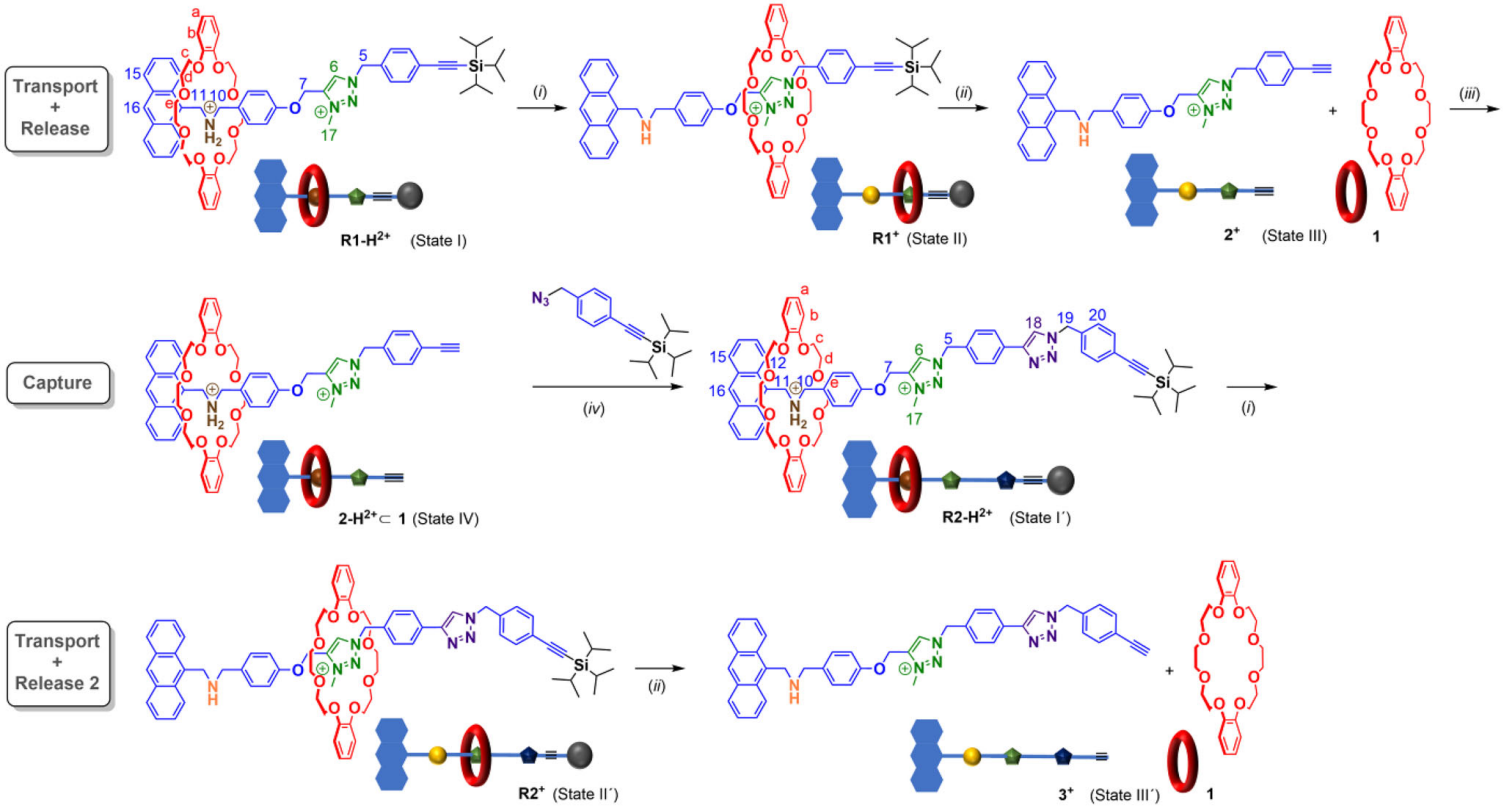
Depiction of the stepwise and directional transport of macrocycle **1** along a recyclable [2]rotaxane transporter. Anions are omitted for clarity (rotaxane **R1‐H^2+^** is used as the bis‐hexafluorophosphate salt, but addition of TFA in step iii leads to a mixture of counteranions). Reagents and conditions i, ii, iii performed *in‐situ* in CD_2_Cl_2_, rotaxane **R2‐H^2+^** was isolated before second transport and release cycle): *i*) BEMP, 1.2 equiv., 5 minutes *ii*) TBAF, 2.0 equiv. 5 minutes, *iii*) trifluoroacetic acid (TFA), 5.0 equiv, 5 minutes (*iv*) Cu(CH_3_CN)_4_PF_6_, 0.9 equiv, NH_4_PF_6_, 4.0 equiv., 48 hours, isolation by size‐exclusion chromatography, 53%.

The stepwise and directional release of macrocycle **1** through the bistable rotaxane **R1‐H^2+^** and recycling back to the interlocked state are dependent on the following design principles (see Figure 2): (a) In the initial protonated state of **R1‐H^2+^**, macrocycle **1** must reside on the DBA recognition site stabilized by hydrogen bonding; (b) upon treatment with base, the macrocycle should shuttle to the MTA station and form **R1^+^** favored by an electrostatic interaction; (c) when exposed to suitable fluoride source, the TIPS stopper should get readily cleaved resulting in the formation of alkyne terminated half‐thread **2^+^** and facilitate release of macrocycle **1** into solution; (d) upon protonation, amine **2^+^** must convert to the corresponding ammonium salt **2‐H^2+^** which should instantly recapture the released macrocycle **1** to form the corresponding pseudorotaxane **2‐H^2+^** ⊂**1**; (e) the pseudorotaxane **2‐H^2+^** ⊂**1** should act as a passive template for CuAAC‐assisted rotaxane formation with azide **4**, thus regenerating the mechanical bond to give the second‐generation rotaxane transporter **R2‐H^2+^** with an elongated thread and (f) **R2‐H^2+^** must also offer stepwise and directional macrocycle release by deprotonation and TIPS‐cleavage, respectively.

The bistable [2]rotaxane **R1‐H^2+^** was synthesized via a passive templated approach: An anthracene‐stoppered secondary ammonium salt featuring a terminal alkyne was reacted with a TIPS‐acetylene functionalized azide **4** in the presence of dibenzo‐24‐crown‐8 macrocycle in a CuAAC reaction. The resulting triazole‐unit was methylated to give rotaxane **R1‐H^2+^** in 74% yield over two steps (see Supporting Information chapter 1.2 for synthesis and characterization).

**Figure 3:**
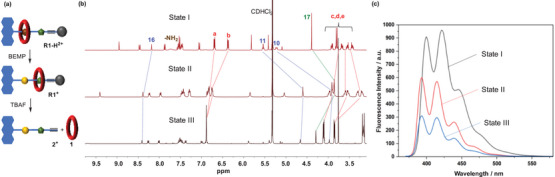
(a) Schematic representation of the stepwise and directional transport (State I→State II→State III), (b) Partial ^1^H NMR (CD_2_Cl_2_, 400 MHz, 298 K) spectra of State I, State II, and State III as prepared *in‐situ*, for numbering see Figure 2, (c) Fluorescence spectra (λ_exc_ = 350 nm, 1×10 ^−5^ M in CH_2_Cl_2_, 298 K) of State I, State II, and State III as prepared *in‐situ*.

With the bistable [2]rotaxane in hand, we went on to perform the *in‐situ* stepwise and directional release of macrocycle **1** from the interlocked state to the solution phase monitored by ^1^H NMR spectroscopy (see Figures 2 and 3). In the ^1^H NMR, **R1‐H^2+^** showed split signals of the macrocyclic a‐H/b‐H protons at 6.69/6.37 ppm (c.f. one signal at 6.88 ppm for free macrocycle **1**), suggesting that the macrocycle is residing on the DBA station stabilized by hydrogen bonds (State I). Addition of 1.2 equivalents phosphazene resin (BEMP) resulted in formation of the deprotonated state **R1^+^** (state II). An upfield shift of the benzylic 11‐H/10‐H proton signals from 5.54/5.22 ppm to 4.60/3.91 ppm is observed, together with an upfield shift of 17‐H methyl proton from 4.40 ppm to 3.86 ppm, indicating a co‐conformational change resulting from binding of the macrocycle to the MTA station through electrostatic interaction. Finally, to achieve the release of macrocycle **1** into the solution, 2.0 equivalents of TBAF were added. Quantitative release of **1** into the solution can be observed, as indicated by a single signal in the aromatic region at 6.88 ppm, together with three sets of signals for the c‐H, d‐H, and e‐H protons between 3.76 and 4.10 ppm (state III). The free half‐thread **2^+^** is also observed (e.g., signal for 17‐H at 4.30 ppm, c.f. 3.86 ppm in **R1^+^**), proving the dissociation of both components (**1** + **2** ^+^) in state III. Thus, stepwise and directional release of macrocycle **1** (State I→State II→State III) was achieved in our [2]rotaxane transporter by successive addition of two chemical stimuli. The action of the [2]rotaxane transporter can also be monitored through fluorescence spectroscopy (see Figure 3c). Upon excitation at 350 nm, State I displayed an emission peak at λ_max_ = 424 nm based on the anthracene emission. Upon conversion to State II, the fluorescence intensity was quenched to 60% of the original intensity, which can be rationalized by PET quenching of the anthracene stopper by the deprotonated amine function as observed in similar anthracene appended [2]rotaxane switches.^[^ ^18^, ^36^ ^]^ In State III, fluorescence was further quenched to 30% of the original intensity, possibly due to an additional intramolecular charge transfer quenching from anthracene to the electron deficient triazolium unit present in noninterlocked **2^+^**.

**Figure 4:**
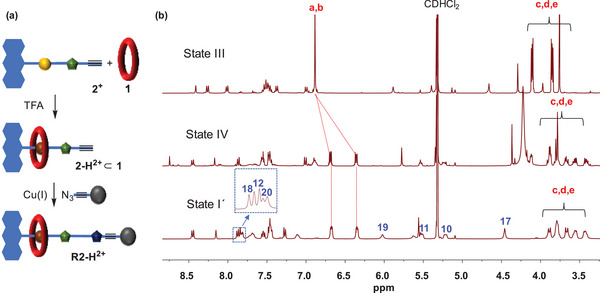
(a) Recapture of released macrocycle to a new interlocked State I´, (b) Partial ^1^H NMR (CD_2_Cl_2_, 400 MHz, 298K) spectra of State III (prepared from **1** to **2^+^**), State IV (as prepared *in‐situ*), and State 1´ (isolated product), for numbering see Figure 2.

In order to investigate the recapture of the macrocycle, we prepared State III *in‐situ* in an NMR tube following State I→State II→State III conversion route (as discussed earlier, also see Supporting Information chapter 3 for experimental details). Then, in the same NMR tube containing state III, 5.0 equivalents of (TFA) were added. By NMR (see Figure 4), 61% of the macrocycle is captured in pseudorotaxane **2‐H^2+^** ⊂**1** (state IV), evidenced by macrocyclic a‐H, b‐H proton signals at 6.69/6.36 ppm and five sets of c‐H, d‐H, and e‐H protons. Thereafter, 3.0 equivalents of stopper azide **4,** 4.0 equivalents of ammonium hexafluorophosphate, and 0.9 equivalents of [Cu(CH_3_CN)_4_]PF_6_ were added in the same NMR tube and the reaction mixture was stirred at room temperature for 48hours. This resulted in a successful CuAAC reaction to give [2]rotaxane **R2‐H^2+^** in 53% isolated yield. Formation of this second‐generation [2]rotaxane **R2‐H^2+^** was confirmed from its ^1^H NMR, which revealed signals for the DBA‐bound macrocycle (e.g., a‐H/b‐H protons at 6.67/6.34 ppm, 11‐H/10‐H protons at 5.51/5.21 ppm), the free triazolium‐station (e.g., 17‐H protons at 4.46 ppm), but also a signal for the novel triazole‐unit (18‐H proton at 7.87 ppm) (see Figure 4).This shows that the DBA and MTA recognition sites are preserved in **R2‐H^2+^** and macrocycle **1** is bound to DBA station analogous to **R1^2+^**, thus resetting the transporter system into a mechanically interlocked state. This resembles State I for **R1‐H^2+^** and can thus be referred to as State I´.

**R2‐H^2+^** and **R1‐H^2+^** possess the same key functionalities for transport and release (DBA and MTA stations, TIPS stopper), with the only difference that **R2‐H^2+^** features an elongated thread with an additional triazole unit stemming from the second click reaction. Thus, we predicted that that **R2‐H^2+^** would also operate as a stepwise and directional macrocycle release system in a second cycle (for structures see Figure 2, for ^1^H NMR spectra of the second cycle see Supporting Information Figure S11). Accordingly, addition of 1.2 equivalents of BEMP converted State I´ to State II´ (resembling State II), with the macrocycle binding to the MTA station (e.g., visible from 17‐H proton at 3.99 ppm). Finally, addition of 2.0 equivalents of TBAF transformed the system to State III´ (resembling State III) resulting in quantitative release of macrocycle **1** and elongated half‐thread **3^+^** into the solution. Thus, the [2]rotaxane **R2‐H^2+^**, which stems from recapture of the macrocycle, allows for a second cycle of macrocycle transport and release with high efficiency. In principle, the resulting thread **3^+^** could undergo additional cycles of macrocycle capture and release in an iterative fashion.

## 3 Conclusion

In conclusion, we have fabricated a [2]rotaxane transporter that enables the release of a crown‐ether macrocycle into the solution by sequential application of two chemical stimuli. In the first step, the deprotonation of the DBA station leads to shuttling towards the MTA station. In the second step, fluoride‐induced TIPS‐cleavage triggers directional release of the macrocycle and the half‐thread in solution. Importantly, the alkyne‐terminated half‐thread allows recapture of the macrocycle by protonation and reestablishment of the mechanical bond by CuAAC click reaction, giving a new interlocked rotaxane featuring an elongated thread. The recycled interlocked state can be used for a second cycle of directional macrocycle release. As a result, this molecular delivery system showcases how molecular information can be directionally propagated, released, and restored in multistate artificial transporter systems enabled by chemical stimuli. We believe that the present transporter system might serve as a platform for functional interlocked molecules, such as stimuli‐responsive drug delivery devices^[^ ^37^, ^38^ ^]^ or organocatalysts,^[^ ^23^, ^39^ ^]^ which is currently under investigation in our laboratories.

## Supporting Information Summary

All experimental details and analytical data can be found in the supporting information. The authors have cited additional references within the supporting information.^[^ ^40^, ^41^ ^]^

## Conflict of Interests

The authors declare no conflict of interest.

## Supporting information



Supporting Information

## Data Availability

The data that support the findings of this study are available in the supplementary material of this article.
