# Supplementary material for: Directional Macrocycle Transport, Release, and Recapture Enabled by a Rotaxane Transporter
Source: Chemistry. 2025 Apr 22;31(37):e202501106. doi: 10.1002/chem.202501106 (PMC12223476; doi:10.1002/chem.202501106)
Supplement: Supplementary file 1 — Supporting Information [file CHEM-31-e202501106-s001.pdf]

# Directional Macrocycle Transport, Release and Recapture enabled by a Rotaxane Transporter

Sohom Kundu,[a],[b]* Shubhadip Mallick,[a] Jan Riebe[a] and Jochen Niemeyer[a]*

[a] Faculty of Chemistry (Organic Chemistry) and
Center for Nanointegration Duisburg-Essen (CENIDE), University of Duisburg-Essen,
Universitätsstrasse 7, 45141 Essen, (Germany)

[b] Research Center for Trustworthy Data Science and Security (UA Ruhr),
Joseph-von-Fraunhofer-Str. 25, 44227 Dortmund (Germany)

email: jochen.niemeyer@uni-due.de

# Table of Contents


**1.** Synthesis

**1.1** General information S2

**1.2** Synthesis and characterization of rotaxanes S3

**2.** *In-situ* two-step directional macrocycle release studies S10

**3.** *In-situ* recycling of released macrocycle S12

**4.** *In-situ* two-step directional macrocycle release studies in 2nd cycle S17

**5.** Synthesis of compound $\mathbf{2}^+$ (*ex-situ*) S18

**6.** 2D, $^{19}$F and $^{31}$P NMR Spectra S25

**7.** IR Spectra S37

**8.** HR-MS Spectra S40

**9.** References S42

# 1. Synthesis

**1.1 General information.** For thin layer chromatography (TLC) analysis, Polygram® SIL G/UV254 TLC plates (silica gel 0.2 mm, 40 × 80 mm) were used. Spots were visualized under a 254/366 nm UV light source. Flash column chromatography was performed using silica gel 60M (40-63 µm) from MACHEREY-NAGEL GmbH & Co. KG. Size exclusion chromatography was performed using SX1-biobeads from Biorad. Aqueous work-ups and column chromatographies were carried out using technical grade solvents. Methyl iodide was purchased from Acros Organics. Ammonium hexafluorophosphate was purchased from Carbolution. Di-*tert*-butyl-dicarbonate was purchased from Fluorochem. Dibenzo-24-crown-8 and tetrakis(acetonitrile)copper(I) hexafluorophosphate were purchased from TCI. Polymer bound BEMP resin, Amberlyst 15, aqueous hexafluorophosphoric acid and tetrabutylammoniumfluoride trihydrate were purchased from Sigma-Aldrich. Methanol, ethanol, acetone and dichloromethane were purchased from Fischer Scientific. $CDCl_3$ and $CD_2Cl_2$ were purchased from Eurisotop. All commercially available chemicals were used without further purification unless stated otherwise. Compounds **3**[1] and **4**[2] were synthesized according to known protocols. The spectral data of compounds **3** and **4** were in full agreement with those in the literature reports.

IR spectra were measured on a Jasco FT/IR-4600 spectrometer. The NMR spectra were recorded with a Bruker Avance NEO 400 spectrometer [$^{1}$H: 400 MHz, $^{19}$F: 376 MHz, $^{31}$P: 162 MHz, $^{13}$C: 101 MHz]. All measurements were performed at room temperature, using [$D_1$]chloroform ($CDCl_3$) or [$D_2$]methylene chloride ($CD_2Cl_2$) as solvents. The chemical shifts are referenced relative to the residual proton signals of the solvent in the $^{1}$H-NMR ($CHCl_3$: δ = 7.26 ppm; $CDHCl_2$: δ = 5.32 ppm; or relative to the solvent signal in the $^{13}$C-NMR ($CDCl_3$: δ = 77.16 ppm; $CD_2Cl_2$: δ = 53.8 ppm. The coupling constants are given in Hertz. The description of the fine structure means: s = singlet, br s = broad singlet, d = doublet, t = triplet, m = multiplet. High resolution ESI mass spectra were recorded on a Bruker Maxis 4G spectrometer. Fluorescence spectra were recorded on a Varian Eclipse fluorescence spectrophotometer.

## 1.2 Synthesis and characterization of rotaxanes

Synthetic route to rotaxane **R1-H$^{2+}$**

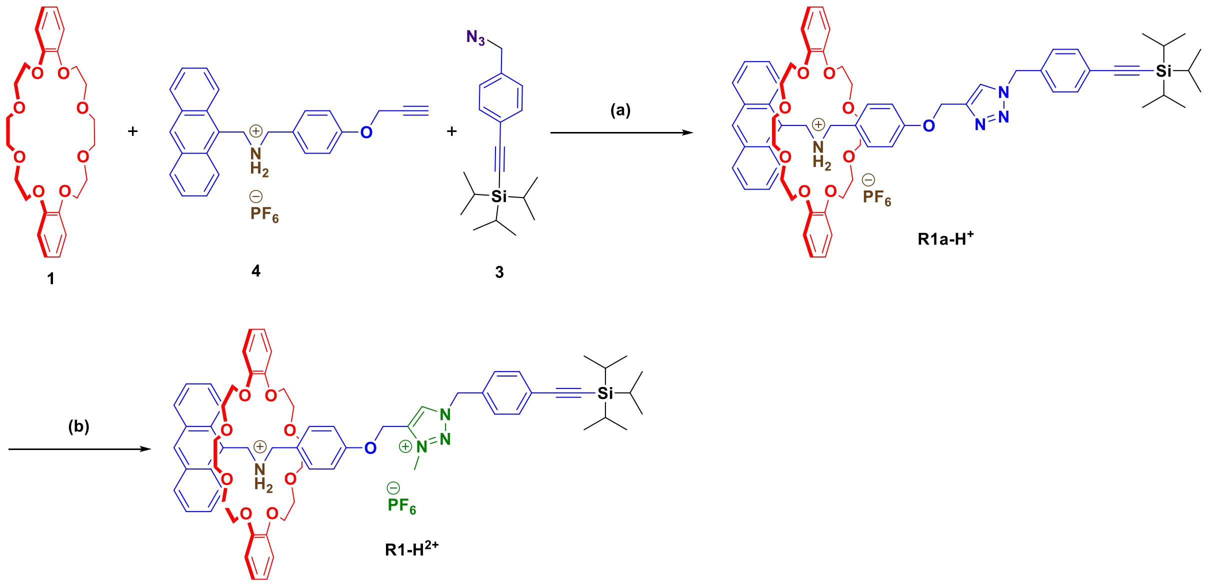


**Scheme1**. Synthesis route to **R1-H$^{2+}$**. (a) tetrakis(acetonitrile)copper(I) hexafluorophosphate (0.9 eq.), DCM, r.t., 12h, 82%; (b) $CH_3I$, r.t., 48h; $NH_4PF_6$, DCM, r.t., 3h, 90%.

## Synthesis and characterization of **R1a-H**$^{+}$

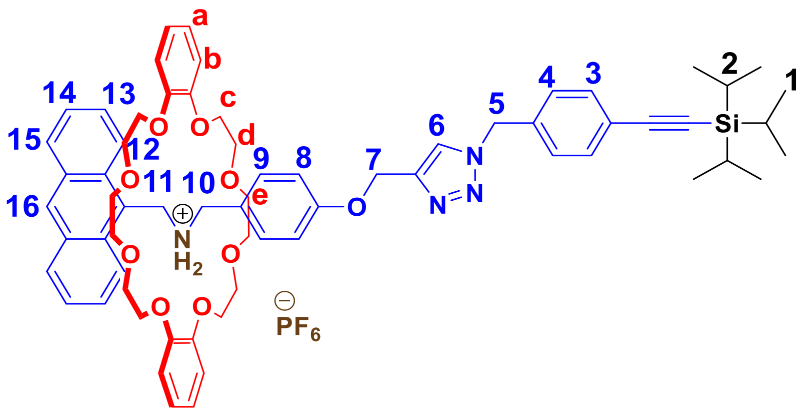

In an oven dried 10 mL Schlenk-tube, dibenzylammonium salt **4** (100 mg, 201 µmol, 1.0 equiv.), macrocycle **1** (108 mg, 241 µmol, 1.2 equiv.) and azide **3** (75.6 mg, 241 µmol, 1.2 equiv.) were added under argon atmosphere. Then dichloromethane (1 mL) was added as solvent and the solution was degassed by purging with argon for ten minutes. Tetrakis(acetonitrile)copper(I) hexafluorophosphate (67.4 mg, 181 µmol, 0.9 equiv.) was added and the mixture was stirred at room temperature for 48 h. The mixture was filtered and the crude product was subjected to column chromatography using dichloromethane/methanol gradient ($R_f$ = 0.3, DCM:MeOH = 99:1). The fraction collected from column chromatography was evaporated, dissolved in DCM (2 mL) and the DCM solution was subjected to size exclusion chromatography using SX-1 bio-beads. The collected fractions were evaporated and dried under vacuum which gave the desired [2]rotaxane **R1a-H**$^{+}$ (as the $PF_6^{-}$-salt, 206 mg, 163 µmol, 82% yield) as a yellow powder.

**$C_{67}H_{81}F_6N_4O_9PSi$:** 1258.4452 g/mol

**$^{1}$H NMR (CDCl$_3$, 400 MHz, 298K)**: $\delta$ = 8.43 (d, $^{3}J$ = 8.9 Hz, 2H, 15-H), 8.12 (s, 1H, 16-H), 7.83 (s, 1H, 6-H), 7.82 (d, $^{3}J$ =8.4 Hz, 2H, 12-H,), 7.69 (br s, 2H, ammonium-N-H), 7.53 (ddd, $^{3}J$ = 8.9 Hz, $^{3}J$ = 6.5 Hz, $^{4}J$ = 1.2 Hz, 2H, 14-H), 7.43 (t, $^{3}J$ =8.4 Hz, 2H, 13-H), 7.42 (d, $^{3}J$ = 8.2 Hz, 2H, 3-H), 7.35 (d, $^{3}J$ = 8.2 Hz, 2H, 9-H), 7.28 (d, $^{3}J$ = 8.2 Hz, 2H, 4-H), 6.94 (d, $^{3}J$ = 8.2 Hz, 2H, 8-H), 6.67 (dd, $^{3}J$ = 6.5 Hz, $^{4}J$ = 3.6 Hz, 4H, a-H), 6.34 (dd, $^{3}J$ = 6.5 Hz, $^{4}J$ = 3.6 Hz, 4H, b-H), 5.52 (s, 2H, 5-H), 5.47 (m, 2H, 11-H), 5.16 (s, 2H, 7-H), 5.14 (m, 2H, 10-H), 3.34-3.91 (m, 24H, c-, d-, e-H), 1.11 (s, 21H, 1-H, 2-H) ppm.

**$^{13}$C NMR (CDCl$_3$, 101 MHz, 298K)**: $\delta$ = 158.3 (C$_{\text{Quaternary-phenyl-C-O}}$), 146.7 (C$_{\text{Quaternary-crown-C-O}}$), 143.7 (C$_{\text{Quaternary-triazole}}$), 134.8 (C$_{\text{Quaternary-phenyl-CH2-triazole}}$), 132.5 (3-C), (130.7 several

overlapping signals, 16-C, two different $C_{Quaternary\text{-}anthracene}$), 130.6 ($C_{Quaternary\text{-}anthracene\text{-}CH2}$), 129.4 (12-C), 128.8 (9-C), 128.2 (4-C), 127.1 (14-C), 125.0 (13-C), 125.0 ($C_{Quaternary\text{-}phenyl\text{-}CH2\text{-}NH2+}$), 123.9 (6-C), 123.8 (15-C), 121.3 (a-C), 115.1 (8-C), 112.0 (b-C), 106.3 ($C_{Quaternary\text{-}phenyl\text{-}ethynyl}$), 91.6 ($C_{ethynyl\text{-}Ph}$), 71.0, 70.3, 68.0 (c-C, d-C and e-C), 61.5 (7-C), 53.4 (5-C), 52.4 (10-C), 45.2 (11-C), 18.6 (1-C), 11.3 (2-C) ppm. $C_{ethynyl\text{-}Si}$ carbon atom was not detected hence could not be assigned.

**COSY (400 MHz / 400 MHz, $CDCl_3$, 298 K)** δ ($^1$H) / δ ($^1$H) = 8.43/7.53 (15-H/14-H), 7.53/8.43 (14-H/15-H), 7.82/7.43 (12-H/13-H), 7.69/5.47, 5.14 (ammonium NH/11-H, 10-H), 7.43/7.82 (13-H/12-H), 7.42/7.28 (3-H/4-H), 7.35/6.94 (9-H/8-H), 7.28/7.42 (4-H, 3-H), 6.94/7.35 (8-H/9-H), 6.67/6.34 (a-H/b-H), 6.34/6.67 (b-H/a-H), 5.47, 5.14/7.69 (11-H, 10-H/ammonium-NH).

**HSQC (400 MHz/101 MHz, $CDCl_3$, 298 K):** *δ* ($^1$H)/*δ* ($^{13}$C) = 8.43/123.8 (15-H/15-C), 8.12/130.7 (16-H/16-C), 7.83/123.9 (6-H/6-C), 7.82/129.4 (12-H/12-C), 7.53/127.1 (14-H/14-C), 7.43/125.0 (13-H/13-C), 7.42/132.50 (3-H/3-C), 7.35/128.8 (9-H/9-C), 7.28/128.2 (4-H/4-C), 6.94/115.1 (8-H/8-C), 6.67/121.3 (a-H/a-C), 6.34/112.0 (b-H/b-C), 5.52/53.4 (5-H/5-C), 5.47/45.2 (11-H/11-C), 5.16/61.5 (7-H/7-C), 5.14/52.4 (10-H/10-C), 3.34-3.91/68.0, 70.3, 71.0 (c-H, d-H, e-H/c-C, d-C, e-C), 1.10/18.6, 11.3 (1-H/1-C, 2-H/2-C).

**HMBC (400 MHz/101 MHz, $CDCl_3$, 298 K):** *δ* ($^1$H)/*δ* ($^{13}$C) = 8.43/125.0, 130.7 (15-H/13-C, 16-C), 8.12/130.7 (16-H/ $C_{Quaternary\text{-}anthracene}$), 7.83/143.7 (6-H/$C_{Quaternary\text{-}triazole}$), 7.82/125.0, 127.1, 130.6, 130.7 (12-H/13-C, 14-C, $C_{Quaternary\text{-}anthracene\text{-}CH2,}$ $C_{Quaternary\text{-}anthracene}$), 7.53/130.7 (14-H/ $C_{Quaternary\text{-}anthracene}$), 7.43/123.8, 130.6 (13-H/15-C, $C_{Quaternary\text{-}anthracene}$), 7.42/106.3, 132.5, 134.8 (4-H/ $C_{Quaternary\text{-}ethynyl,}$ 3-C, $C_{Quaternary\text{-}phenyl\text{-}CH2\text{-}triazole}$ ), 7.35/52.4, 128.8, 158.3 (9-H/10-C, 9-C, $C_{Quaternary\text{-}phenyl\text{-}C\text{-}O}$), 7.28/53.4, 123.9, 128.2 (4-H/5-C, 6-C, 4-C), 6.94/115.1, 125.0, 158.3 (8-H/8-C, $C_{Quaternary\text{-}phenyl\text{-}CH2\text{-}NH2+,}$ $C_{Quaternary\text{-}phenyl\text{-}C\text{-}O}$), 6.67/112.0, 146.7 (a-H/b-C, $C_{Quaternary\text{-}crown\text{-}C\text{-}O}$), 6.34/121.0, 146.7 (b-H/a-C, $C_{Quaternary\text{-}crown\text{-}C\text{-}O}$), 5.52/123.9, 128.2, 134.8 (5-H/6-C, 4-C, $C_{Quaternary\text{-}CH2\text{-}triazole}$), 5.47/130.7 (11-H/ $C_{Quaternary\text{-}anthracene}$), 5.16/123.9, 143.7, 158.3 (7-H/6-C, $C_{Quaternary\text{-}triazole,}$ $C_{Quaternary\text{-}phenyl\text{-}C\text{-}O}$), 5.14/125.0, 128.8 (10-H/$C_{Quaternary\text{-}phenyl\text{-}ammonium,}$ 9-C), 3.34-3.91/68.0, 70.3, 71.0 (c-H, d-H, e-H/c-C, d-C, e-C), 1.11/11.3, 18.6, (1-H, 2-H/1-C, 2-C).

**$^{19}$F NMR ($CDCl_3$, 376 MHz, 298 K)** *δ* = -73.7 (d, *J* = 711 Hz, $PF_6^-$ )

**$^{31}$P NMR ($CDCl_3$, 162 MHz, 298 K)** *δ* = -144.5 (sept, *J* = 711 Hz, $PF_6^-$ )

**HR-MS** (ESI-pos, MeOH): *m/z* = 1113.5771 [**R1a-H**]$^+$, calcd. 1113.5767 for $C_{67}H_{81}N_4O_9Si^+$.

**IR (ATR-FT):** $\tilde{\nu}$ = 2939, 2866, 1591, 1505, 1453, 1253, 1217, 1123, 1108, 1052, 955, 840, 746 cm$^{-1}$.

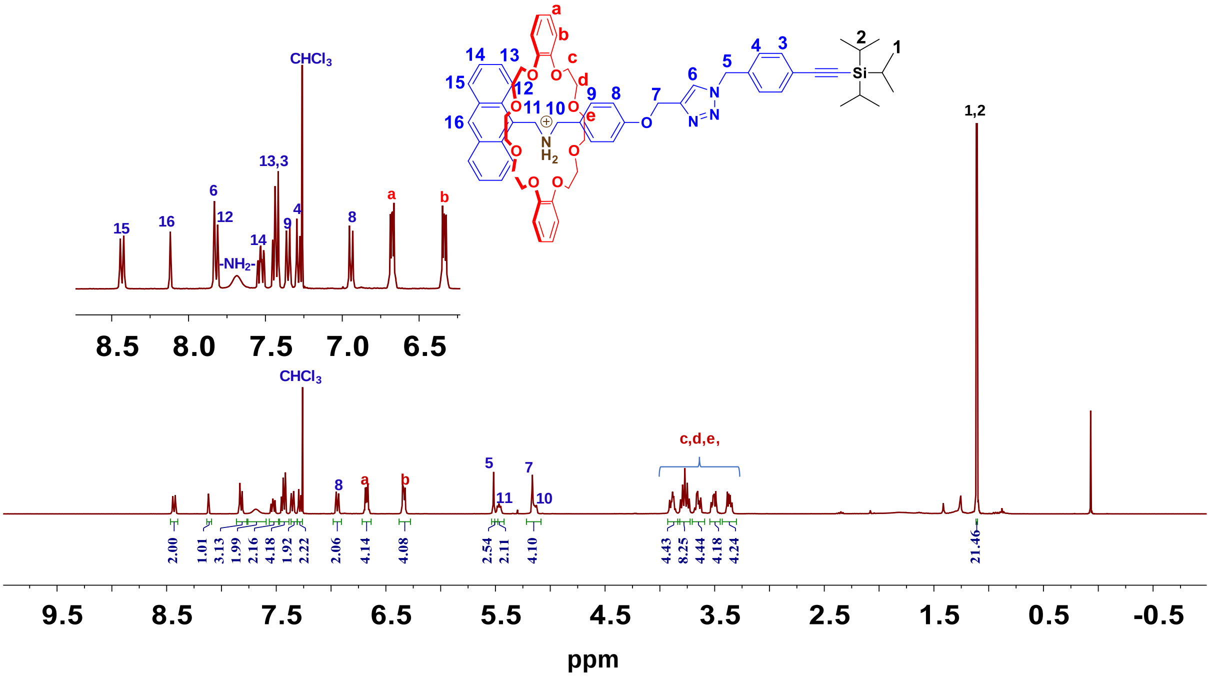

**Figure S1.** $^1$H NMR spectrum ($CDCl_3$, 400 MHz, 298K) of **R1-H**$^+$ (as the $PF_6^-$-salt).

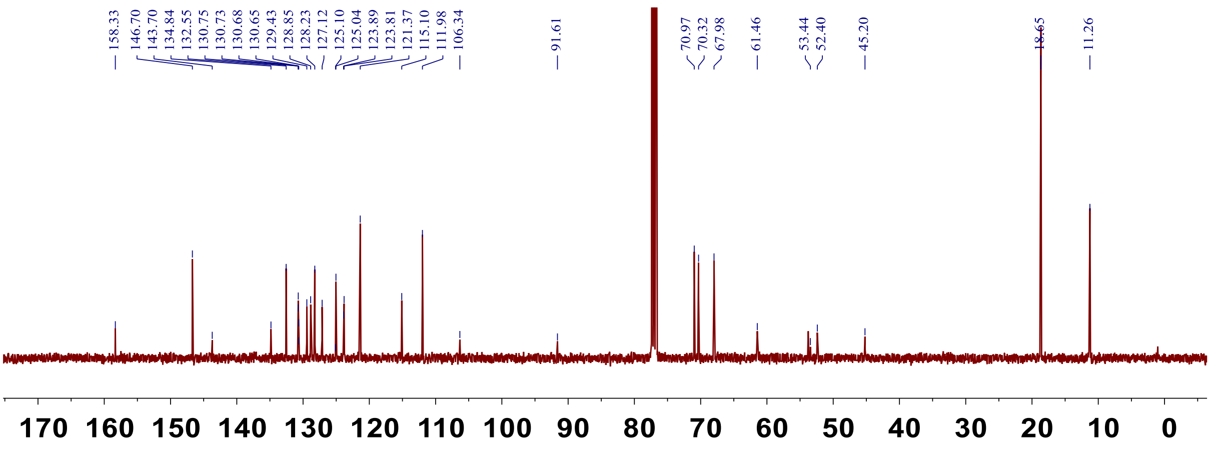

**Figure S2.** $^{13}$C NMR spectrum ($CDCl_3$, 101 MHz, 298K) of **R1-H**$^+$ (as the $PF_6^-$-salt).

Synthesis and characterization of **R1-H$^{2+}$**

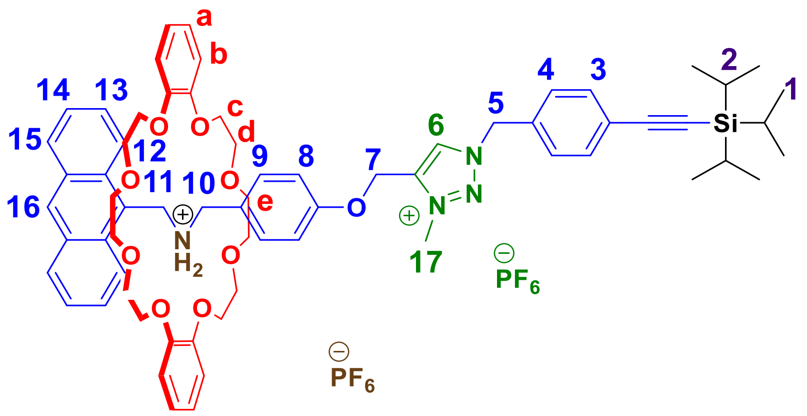


In an oven dried 5 mL Schlenk-flask, **R1a-H$^{+}$** (as the as the $PF_6^{-}$-salt, 100 mg, 79.1 µmol, 1.0 equiv.) was dissolved in an excess amount of iodomethane (5 mL). The reaction mixture was stirred at room temperature for 48 h. Then the excess of iodomethane was evaporated and the resulting yellow solid was dissolved in dichloromethane (5 mL). Ammonium hexafluorophosphate (25.7 mg, 158 µmol, 2.0 equiv.) was suspended into the reaction flask and the reaction mixture was stirred at room temperature for another 3 h. Then, the reaction mixture was filtered and the filtrate was evaporated and dried under vacuum which gave the desired product **R1-H$^{2+}$** as yellow solid (as the bis-$PF_6^{-}$-salt, 102 mg, 71.0 µmol, 90% yield).

**$C_{68}H_{84}F_{12}N_4O_9P_2Si$:** 1418.4444 g/mol

**$^{1}$H NMR ($CD_2Cl_2$, 400 MHz, 298K)**: $\delta$ = 8.95 (s, 1H, 6-H), 8.46 (d, $^{3}J$ = 8.9 Hz, 2H, 15-H), 8.18 (s, 1H, 16-H), 7.86 (d, $^{3}J$ =8.4 Hz, 2H, 12-H,), 7.72 (br s, 2H, ammonium-NH), 7.55 (m, 2H, 14-H), 7.53 (m, 2H, 3-H), 7.52 (m, 2H, 4-H), 7.47 (m, 2H, 9-H), 7.46 (m, 2H, 13-H), 7.04 (d, $^{3}J$ = 8.2 Hz, 2H, 8-H), 6.69 (dd, $^{3}J$ = 6.5 Hz, $^{4}J$ = 3.6 Hz, 4H, a-H), 6.36 (dd, $^{3}J$ = 6.5 Hz, $^{4}J$ = 3.6 Hz, 4H, b-H), 5.82 (s, 2H, 5-H), 5.54 (m, 2H, 11-H), 5.41 (s, 2H, 7-H), 5.22 (m, 2H, 10-H), 4.40 (s, 3H, 17-H), 3.42-3.90 (m, 24H, c-, d-, e-H), 1.12 (s, 21H, 1-H, 2-H) ppm.

**$^{13}$C NMR ($CD_2Cl_2$, 101 MHz, 298K)**: $\delta$ = 157.0 ($C_{Quaternary\text{-}phenyl\text{-}C\text{-}O}$), 146.8 ($C_{Quaternary\text{-}crown\text{-}C\text{-}O}$), 140.1 ($C_{Quaternary\text{-}triazolium}$), 132.8 (4-C), 130.8 (overlapping signals of two $C_{Quaternary\text{-}anthracene}$), 130.7 (16-C), 130.0 (6-C), 129.4 (12-C), 129.4 (3-C), 129.0 (9-C), 127.1 (14-C), 126.8 ($C_{Quaternary\text{-}phenyl\text{-}CH2\text{-}NH2+}$), 125.4 ($C_{Quaternary\text{-}phenyl\text{-}CH2\text{-}triazolium}$), 125.0 (13-C), 123.8 (15-C), 121.4 ($C_{Quaternary\text{-}anthracene\text{-}CH2}$ possibly, could not be assigned unambiguously), 121.2 (a-C), 115.0 (8-C), 112.0 (b-C), 105.7 ($C_{Quaternary\text{-}phenyl\text{-}ethynyl}$), 92.9 ($C_{ethynyl\text{-}phenyl}$), 71.0, 70.4, 68.0 (c-C, d-C, e-

C), 58.6 (7-C), 57.3 (5-C), 52.2 (10-C), 45.3 (11-C), 39.3 (17-C), 18.3 (1-C), 11.2 (2-C) ppm. $C_{ethynyl\text{-}Si}$ carbon atom was not detected hence could not be assigned.

**COSY (400 MHz / 400 MHz, $CD_2Cl_2$, 298 K)** δ ($^1$H) / δ ($^1$H) = 8.46/7.55 (15-H/14-H), 7.86/7.46 (12-H/13-H), 7.72/5.54 (ammonium-NH/11-H), 7.72/5.22 (ammonium-NH/10-H), 7.55/8.46 (14-H/15-H), 7.47/7.04 (9-H/8-H), 7.46/7.86 (13-H/12-H), 7.04/7.47 (8-H/9-H), 6.69/6.36 (a-H/b-H), ,6.36/6.69 (b-H/a-H),. 5.54/7.72 (11-H/ammonium-NH), 5.22/7.72 (10-H/ammonium-NH).

**HSQC (400 MHz/101 MHz, $CD_2Cl_2$, 298 K):** *δ* ($^1$H)/*δ* ($^{13}$C) = 8.95/130.0 (6-H/6-C), 8.46/123.8 (15-H/15-C), 8.18/130.7 (16-H/16-C), 7.86/129.4 (12-H/12-C), 7.55/127.1 (14-H/14-C), 7.53/129.4 (3-H/3-C), 7.52/132.8 (4-H/4-C), 7.47/129.0 (9-H/9-C), 7.46/125.0 (13-H/13-C), 7.04/115.0 (8-H/8-C), 6.69/121.2 (a-H/a-C), 6.36/112.0 (b-H/b-C), 5.82/57.3 (5-H/5-C), 5.54/45.3 (11-H/11-C), 5.41/58.6 (7-H/7-C), 5.22/52.2 (10-H/10-C), 4.40/39.3 (17-H/17-C), 3.42-3.90/71.0, 70.4, 68.0 (c-H,d-H, e-H/c-C, d-C, e-C), 1.12/11.2, 18.3 (1-H, 2-H/1-C, 2-C).

**HMBC (400 MHz/101 MHz, $CD_2Cl_2$, 298 K):** *δ* ($^1$H)/*δ* ($^{13}$C) = 8.95/140.1 (6-H/$C_{Quaternary\text{-}triazolium}$), 8.46/125.0, 130.7, 130.8 (15-H/ 13-C, 16-C, $C_{Quaternary\text{-}anthracene}$), 8.18/130.8 (16-H/ $C_{Quaternary\text{-}anthracene}$) 7.86/127.1, 130.8 (12-H/14-C, $C_{Quaternary\text{-}anthracene}$), 7.55/129.4, 130.8 (14-H/12-C, $C_{Quaternary\text{-}anthracene}$), 7.53/105.7 (3-H/$C_{Quaternary\text{-}phenyl\text{-}ethynyl}$), 7.52/57.3, 125.4 (4-H, 5-C, $C_{Quaternary\text{-}phenyl\text{-}CH2\text{-}triazolium}$), 7.47/52.2, 129.0, 157.0 (9-H/ 10-C, 9-C, $C_{Quaternary\text{-}phenyl\text{-}C\text{-}O}$), 7.46/123.8, 130.8 (13-H/15-C, $C_{Quaternary\text{-}anthracene}$), 7.04/126.8 (8-H/ $C_{Quaternary\text{-}phenyl\text{-}CH2\text{-}NH2+}$), 6.69/112.0 (a-H/b-C), 6.36/121.2, 146.8 (b-H/a-C, $C_{Quaternary\text{-}crown\text{-}C\text{-}O}$), 5.82/130.0 (5-H/6-C), 5.41/130.0, 140.1, 157.0 (7-H/6-C, $C_{Quaternary\text{-}triazolium,}$ $C_{Quaternary\text{-}phenyl\text{-}C\text{-}O}$), 3.42-3.90/71.0, 70.4, 68.0 (c-H,d-H, e-H/c-C, d-C, e-C), 1.12/11.2, 18.3 (1-H, 2-H/1-C, 2-C).

**$^{19}$F NMR ($CD_2Cl_2$, 376 MHz, 298 K)** *δ* = -73.2 (d, *J* = 711 Hz, $PF_6^-$ )

**$^{31}$P NMR ($CD_2Cl_2$, 162 MHz, 298 K)** *δ* = -144.6 (sept, *J* = 711 Hz, $PF_6^-$ )

**HR-MS** (ESI-pos, MeOH): *m/z* = 564.3000 [**R1-H**]$^{2+}$, calcd. 564.2998 for $C_{68}H_{84}N_4O_9Si^{2+}$.

**IR (ATR-FT):** $\tilde{\nu}$ = 3320, 3138, 3058, 2922, 2863, 1591, 1503, 1450, 1248, 1207, 1121, 1102, 1053, 952, 831, 734, 677, 555 cm$^{-1}$.

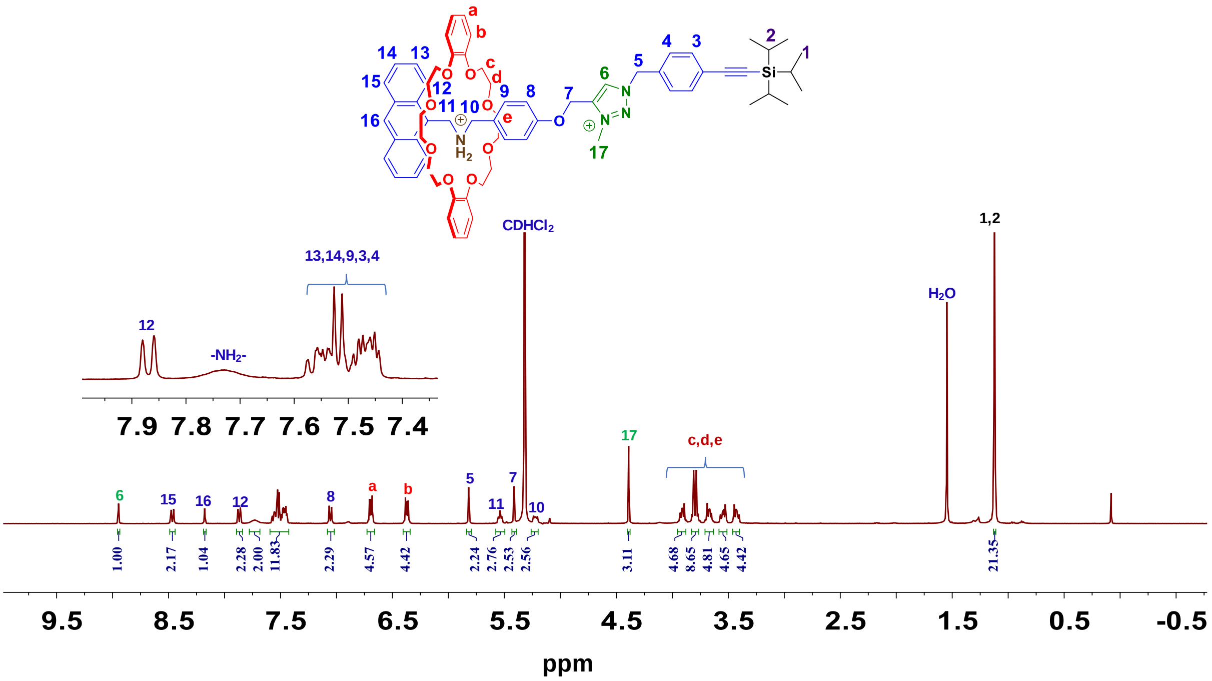


**Figure S3.** $^{1}H$ NMR spectrum ($CD_2Cl_2$, 400 MHz, 298K) of **R1-H$^{2+}$** (as the bis-$PF_6^{-}$-salt).

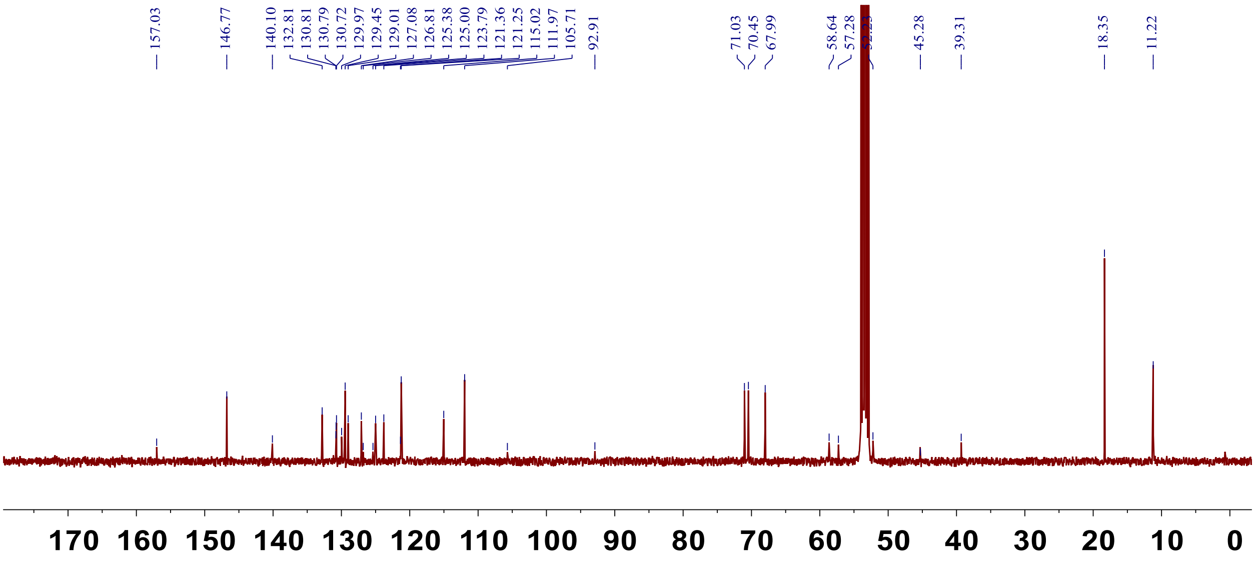


**Figure S4.** $^{13}C$ NMR spectrum ($CD_2Cl_2$, 101 MHz, 298K) of **R1-H$^{2+}$** (as the $PF_6^{-}$-salt).

## 2. *In-situ* two-step directional macrocycle release studies

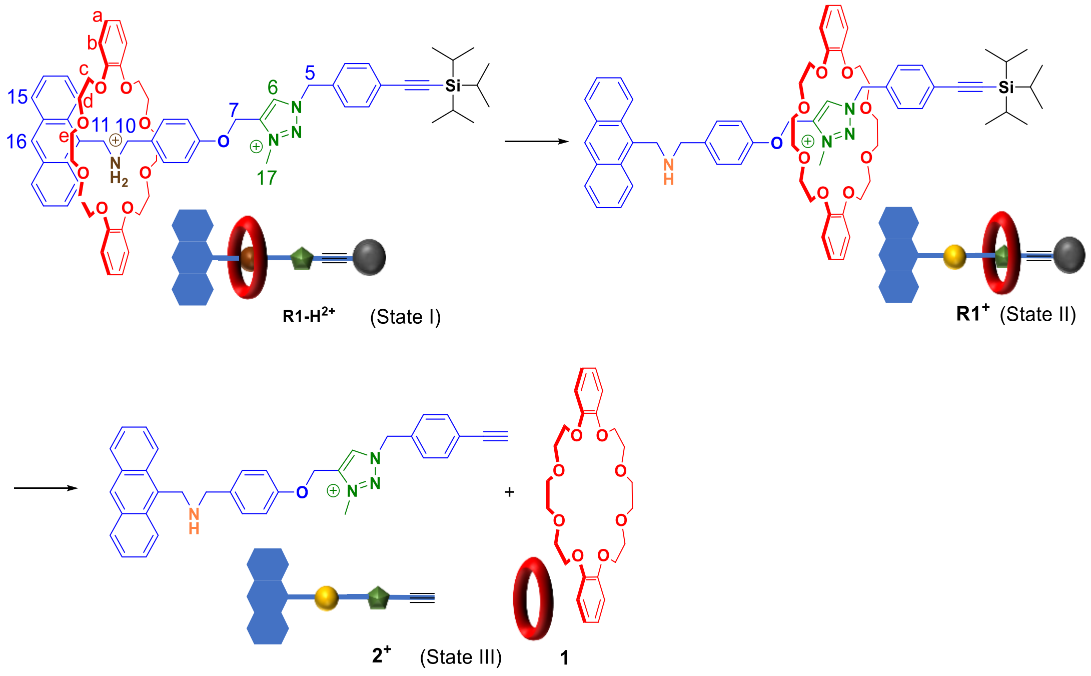


**State I**: An NMR tube was loaded with **R1-H$^{2+}$** (as the bis-$PF_6^-$-salt, 5.20 mg, 3.60 µmol, 1.0 equiv.) and dissolved in 500 µL of $CD_2Cl_2$. The $^1$H NMR was recorded (state I).

**State II**: In the same NMR tube, polymer bound 2-*tert*-Butylimino-2-diethylamino-1,3-dimethylperhydro-1,3,2-diazaphosphorin resin (BEMP) (2.20 mg, 4.32 µmol, 1.2 equiv.) with 1% cross-linking was added considering 2.0 mmol/gm base loading. After 5 minutes, $^1$H NMR was recorded which shows formation of **R1$^+$** (state II).

**State III:** In the same NMR tube containing **R1$^+$**, TBAF•3$H_2O$ (2.41 mg, 7.20 µmol, 2.0 equiv.) was added. After 5 minutes, $^1$H NMR was recorded which shows formation of **2$^+$** and free **1** (state III).

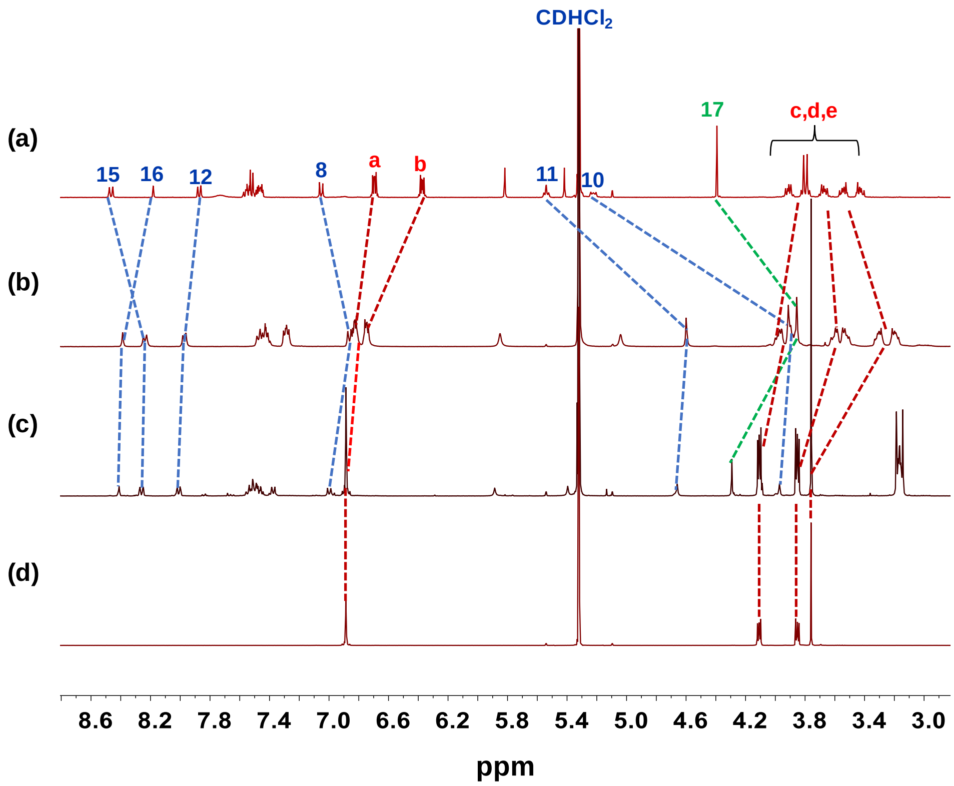


**Figure S5**. $^{1}$H NMR ($CD_2Cl_2$, 400 MHz, 298K) comparison spectra of (a) **R1-H$^{2+}$** (state I) (b) **R1$^{+}$** (state II) (c) **2$^{+}$** + **1** (state III), (d) free macrocycle **1**.

## 3. *In-situ* recycling of released macrocycle

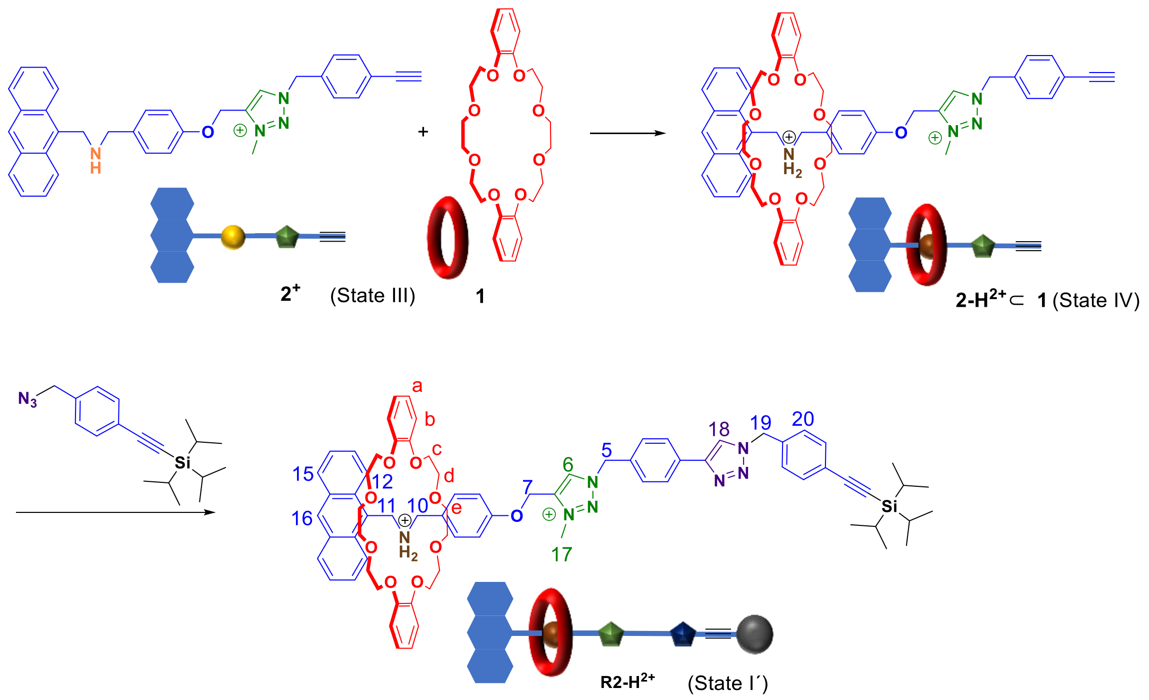


In an NMR tube, state III ($2^+$ + **1**) was prepared starting with **R1-H**$^{2+}$ (as the bis-$PF_6^-$-salt, 5.2 mg, 3.60 µmol, 1.0 equiv.) (following state I→state II→state III conversion as prepared in chapter 2) in 500 µL $CD_2Cl_2$. Thereafter, TFA (2.05 mg, 18.0 µmol, 5.0 equiv. added as 6.75 µL from a standard solution of 5 µL TFA in 25 µL of $CD_2Cl_2$ ) was added into the same NMR tube and an NMR-spectrum was recorded subsequently which corresponded to formation of pseudorotaxane **2-H**$^{2+}$⊂**1** in 61% yield (by integrating a/b-H proton signal at 6.69/6.36 of **2-H**$^{2+}$⊂**1** and a/b-H signal of free **1** at 6.88 ppm). Then in the same NMR tube, azide **3** (3.38 mg, 10.8 µmol, 3.0 equiv.), $NH_4PF_6$ (2.34 mg, 14.4 µmol, 4.0 equiv.) and tetrakis(acetonitrile)copper(I) hexafluorophosphate (1.20 mg, 3.24 µmol, 0.9 equiv.) were added and the mixture was stirred (a micro stir bar was added inside the NMR tube) at room temperature for 48h. Then the reaction mixture was filtered and the filtrate was evaporated, dissolved in 1 mL DCM and was directly subjected to size exclusion chromatography using SX-1 bio-beads and DCM as an eluent. The collected fractions were evaporated and dried which gave [2]rotaxane **R2-H**$^{2+}$ in 53% yield (as the bis-$PF_6^-$-salt, 3.35 mg, 1.91 µmol). It was characterized and reused in the second cycle for the directional transport without further purification.

**$C_{77}H_{91}F_{12}N_7O_9P_2Si$**: 1575.6204 g/mol

**$^{1}$H NMR** (**$CD_2Cl_2$, 400 MHz, 298K**): $\delta$ = 8.46 (d, $^{3}J$ = 8.9 Hz, 2H, 15-H), 8.15 (s, 1H, 16-H), 7.87 (s, 1H, 18-H), 7.84 (d, $^{3}J$ =8.4 Hz, 2H, 12-H), 7.82 (m, 2H, 20-H), 7.72 (br s, 2H, ammonium-NH), 7.69 (m, 2H, 21-H), 7.55 (m, 2H, 14-H), 7.48 (s, 1H, 6-H), 7.47 (m, 2H, 3-H), 7.46 (m, 2H, 13-H), 7.45 (m, 2H, 9-H), 7.27 (d, $^{3}J$ = 8.2 Hz, 2H, 4-H), 7.10 (m, 2H, 8-H), 6.67 (dd, $^{3}J$ = 6.5 Hz, $^{4}J$ = 3.6 Hz, 4H, a-H), 6.34 (dd, $^{3}J$ = 6.5 Hz, $^{4}J$ = 3.6 Hz, 4H, b-H), 6.02 (s, 2H, 19-H), 5.62 (s, 2H, 7-H), 5.56 (s, 2H, 5-H), 5.51 (m, 2H, 11-H), 5.21 (m, 2H, 10-H), 4.46 (s, 3H, 17-H), 3.42-3.90 (m, 24H, c-, d-, e-H), 1.12 (s, 21H, 1,2-H) ppm.

**$^{13}$C NMR ($CDCl_3$, 101 MHz, 298K)**: $\delta$ = 157.3 ($C_{Quaternary\text{-}phenyl\text{-}C\text{-}O}$), 146.9 ($C_{Quaternary\text{-}triazole}$), 146.7 ($C_{Quaternary\text{-}crown\text{-}C\text{-}O}$), 135.1 ($C_{Quaternary\text{-}phenyl\text{-}CH2\text{-}triazolium}$), 132.5 (overlapping signals, 3-C, $C_{Quaternary\text{-}phenyl\text{-}CH2\text{-}triazole}$), 132.1 ($C_{Ar}$) , 130.8 ($C_{Quaternary\text{-}anthracene}$), 130.7 (16-C), 130.2 (21-C), 129.4 (12-C), 128.9 (9-C), 128.0 (4-C), 127.1 (14-C), 126.5 ($C_{Ar}$), 126.2 (20-C), 125.0 (13-C), 123.9 ($C_{Ar}$), 123.8 (15-C), 121.2 (a-C), 120.5 (18-C), 115.2 (8-C), 112.0 (b-C), 106.1 ($C_{Quaternary\text{-}phenyl\text{-}triazole}$), 91.7 ($C_{ethynyl\text{-}phenyl}$), 71.1, 70.5, 68.0 (c-C, d-C and e-C) , 58.7 (7-C), 56.8 (19-C), 53.9 (5-C), 52.2 (10-C), 45.2 (11-C), 39.2 (17-C), 18.3 (2-C), 11.2 (1-C) ppm. Signals at 132.1, 126.5 and 123.9 ppm could not be assigned. Vice versa, signals for 6-C, $C_{Quaternary\text{-}ethynyl\text{-}Si}$, $C_{Quaternary\text{-}phenyl\text{-}ethynyl}$, $C_{Quaternary\text{-}phenyl\text{-}CH2\text{-}NH2+}$, $C_{Quaternary\text{-}anthracene\text{-}CH2}$, $C_{Quaternary\text{-}triazolium}$ could not be found/assigned.

**COSY (400 MHz / 400 MHz, $CD_2Cl_2$, 298 K)** δ ($^{1}$H) / δ ($^{1}$H) = 8.46/7.55 (15-H/14-H), 7.84/7.46 (12-H/13-H), 7.82/7.69 (20-H/21-H), 7.72/5.51, 5.21 (ammonium-NH/11-H, 10-H), 7.69/7.82 (21-H/20-H), 7.55/8.46 (14-H/15-H), 7.47/7.27 (3-H/4-H), 7.46/7.84 (13-H/12-H), 7.45/7.10 (9-H/8-H), 7.27/7.47 (4-H/3-H), 7.10/7.45 (8-H/9-H), 6.67/6.34 (a-H/b-H), 6.34/6.67 (b-H/a-H), 5.21, 5.51/7.72 (10-H, 11-H/ ammonium-NH/).

**HSQC (400 MHz/101 MHz, $CD_2Cl_2$, 298 K):** $\delta$ ($^{1}$H)/$\delta$ ($^{13}$C) = 8.46/123.8 (15-H/15-C), 8.15/130.7 (16-H/16-C), 7.87/120.5 (18-H/18-C), 7.84/129.4 (12-H/12-C), 7.82/126.2 (20-H/20-C), 7.69/130.2 (21-H/21-C), 7.55/127.1 (14-H/14-C), 7.47/132.5 (3-H/3-C), 7.46/125.0 (13-H/13-C), 7.45/128.9 (9-H/9-C), 7.27/128.0 (4-H/4-C), 7.10/115.2 (8-H/8-C), 6.67/121.2 (a-H/a-C), 6.34/112.0 (b-H/b-C), 6.02/56.8 (19-H/19-C), 5.62/58.7 (7-H/7-C), 5.56/53.9 (5-H/5-C), 5.51/45.2 (11-H/11-C), 5.21/52.2 (10-H/10-C), 4.46/39.2 (17-H/17-C), 3.42-3.90/71.1, 70.5, 68.0 (c-H,d-H, e-H/c-C, d-C, e-C), 1.12/11.2, 18.3 (1-H, 2-H/1-C, 2-C). 6-C was not detected.

**HMBC (400 MHz/101 MHz, $CD_2Cl_2$, 298 K):** $\delta$ ($^{1}$H)/$\delta$ ($^{13}$C) = 8.46/125.0, 130.7, 130.8 (15-H/13-C, 16-C, $C_{Quaternary\text{-}anthracene}$), 8.15/130.8 (16-H/$C_{Quaternary\text{-}anthracene}$), 7.87/146.9 (18-

H/C$_{Quaternary-triazole}$), 7.84/127.1, 130.8 (12-H/14-C, C$_{Quaternary-anthracene}$), 7.69/132.5 (21-H/C$_{Quaternary-phenyl--CH2-triazole}$), 7.55/130.8 (14-H/C$_{Quaternary-anthracene}$), 7.47/106.1, 135.1 (3-H/C$_{Quaternary-phenyl-triazole,}$ C$_{Quaternary-phenyl-CH2-triazolium}$), 7.46/123.8, 130.8 (13-H/15-C, C$_{Quaternary-anthracene}$), 7.45/157.3 (9-H/ C$_{Quaternary-phenyl-C-O}$), 7.27/53.9, 132.5 (4-H/5-C, 3-C), 6.34/121.2, 146.7 (b-H/ a-C, C$_{Quaternary-crown-C-O}$), 5.56/128.0, 135.1 (5-H/4-C, C$_{Quaternary-Ph-CH2-triazolium}$), 1.12/11.2, 18.3 (1-H, 2-H/1-C, 2-C).

**$^{19}$F NMR ($CD_2Cl_2$, 376 MHz, 298 K)** $\delta$ = -73.2 (d, $J$ = 711 Hz, $PF_6^-$ )

**$^{31}$P NMR ($CD_2Cl_2$, 162 MHz, 298 K)** $\delta$ = -144.6 (sept, $J$ = 711 Hz, $PF_6^-$ )

**HR-MS** (ESI-pos, MeOH): $m/z$ = 642.8316 [**R2-H**]$^{2+}$, calcd. 642.8318 for $C_{77}H_{91}N_7O_9Si^{2+}$.

**IR (ATR-FT):** $\tilde{\nu}$ = 2956, 2925, 2861, 1507, 1459, 1253, 1121, 1101, 1053, 1019, 955, 842, 801, 744 cm$^{-1}$.

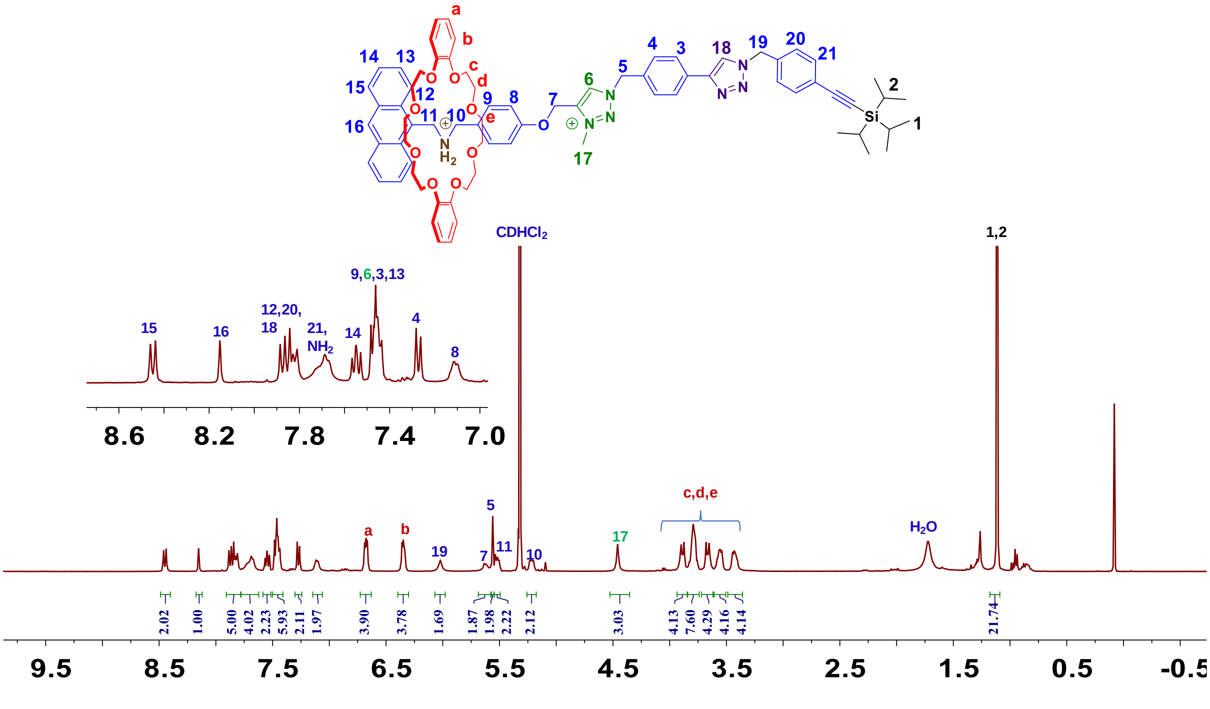


**Figure S6.** $^{1}$H NMR spectrum ($CD_2Cl_2$, 400 MHz, 298K) of **R2-H$^{2+}$** (as the bis-$PF_6^-$-salt).

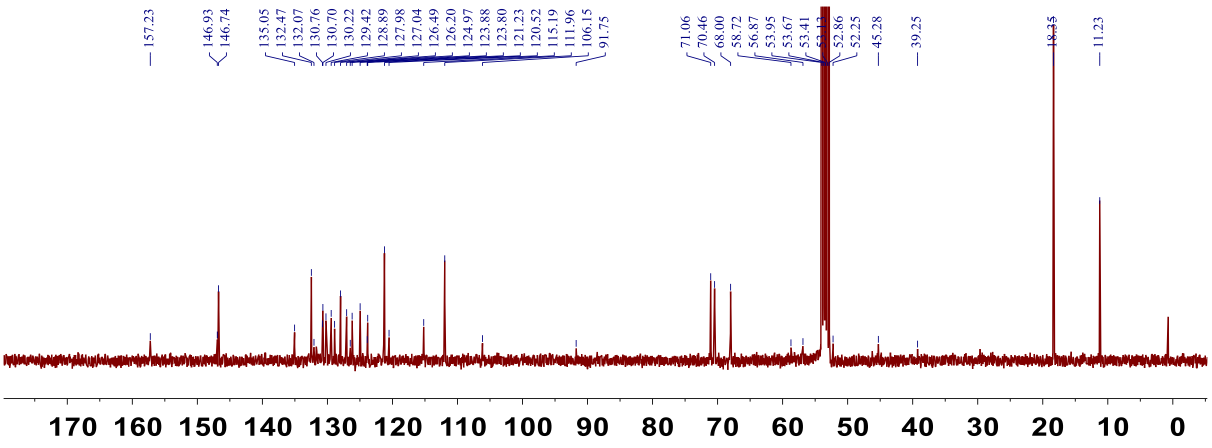

**Figure S7.** $^{13}C$ NMR spectrum ($CD_2Cl_2$, 101 MHz, 298K) of **R2-H$^{2+}$** (as the bis-$PF_6^-$-salt).

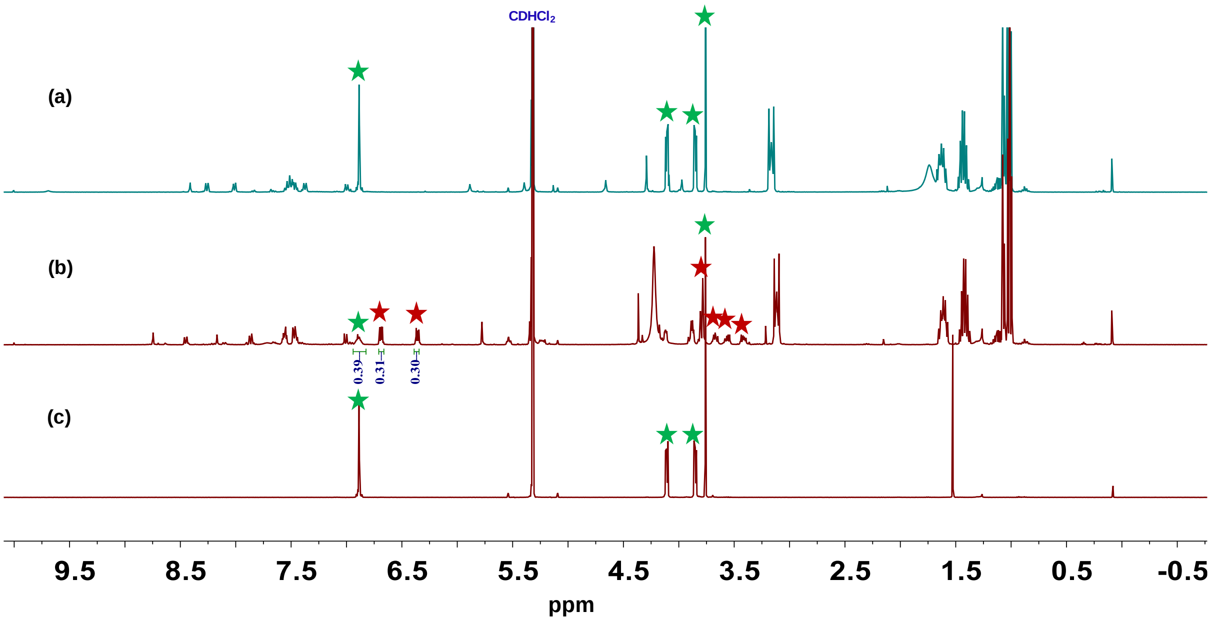


**Figure S8.** $^1H$ NMR ($CD_2Cl_2$, 400 MHz, 298K) comparison spectra of (a) **2$^+$** + **1**, (b) pseudorotaxane **2-H$^{2+}$⊂1**, (c) free macrocycle **1**. Red asterisk marked proton signals corresponds to the macrocyclic protons in the pseudorotaxane while green asterisk marked protons represent the free macrocycle.

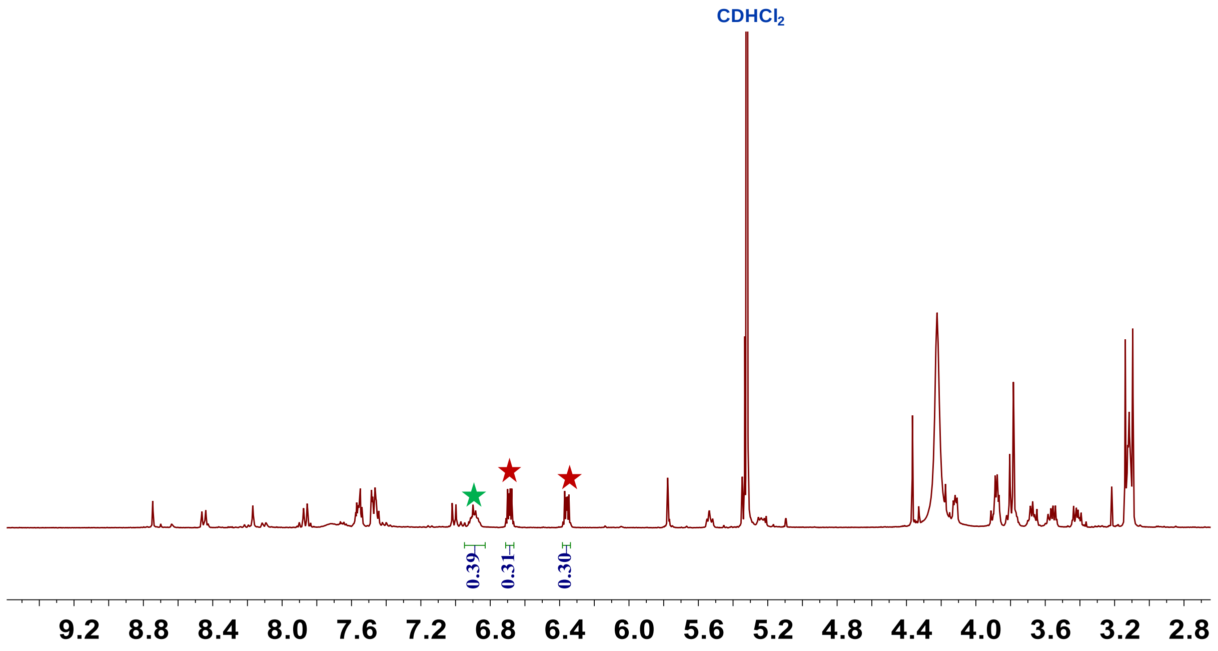


**Figure S9.** $^{1}$H NMR ($CD_2Cl_2$, 400 MHz, 298K) spectra of **2-H$^{2+}$⊂1** which is present in 61% in the mixture (39% of the macrocycle is unbound free macrocycle, as determined by integrating red asterisk marked a/b-H proton signal at 6.69/6.36 of **2-H$^{2+}$⊂1** and green asterisk marked a/b-H signal of free **1** at 6.88 ppm).

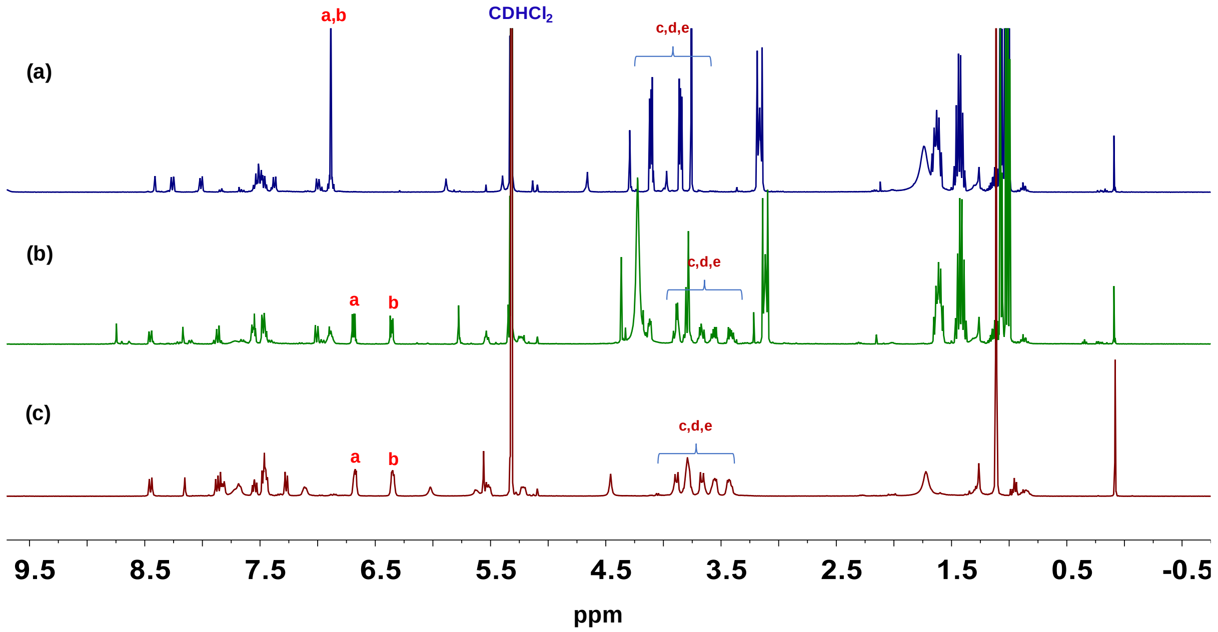


**Figure S10**. $^{1}$H NMR ($CD_2Cl_2$, 400 MHz, 298K) comparison spectra of (a) **2$^{+}$** + **1**, (b) pseudorotaxane **2-H$^{2+}$⊂1** , (c) **R2-H$^{2+}$.**

## 4. *In-situ* two-step directional macrocycle release studies in 2nd cycle

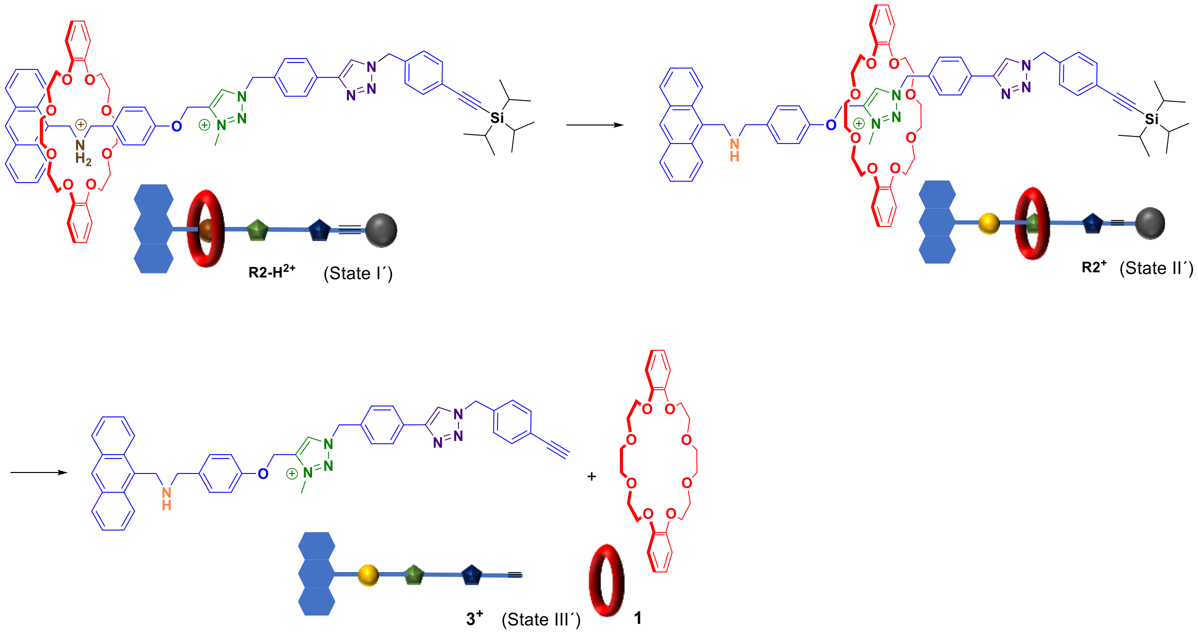


**State I'**: An NMR tube was loaded with **R2-H**$^{2+}$ (as the bis-$PF_6^-$-salt, 3.35 mg, 1.91 µmol, 1.0 equiv. recycled from 1st cycle) and dissolved in 500 µL of $CD_2Cl_2$. The $^1$H NMR was recorded (State I').

**State II'**: In the same NMR tube, polymer bound 2-*tert*-Butylimino-2-diethylamino-1,3-dimethylperhydro-1,3,2-diazaphosphorin resin (BEMP), (1.17 mg, 2.30 µmol, 1.2 equiv.) with 1% cross-linking was added considering 2.0 mmol/gm base loading. After 5 minutes, $^1$H NMR was recorded which shows formation of **R2**$^+$(state II').

**State III':** In the same NMR tube containing **R2**$^+$, TBAF•3$H_2O$ (1.28 mg, 3.82 µmol, 2.0 equiv.) was added. After 5 minutes, $^1$H NMR was recorded which shows formation of **3**$^+$ and free **1** (state III').

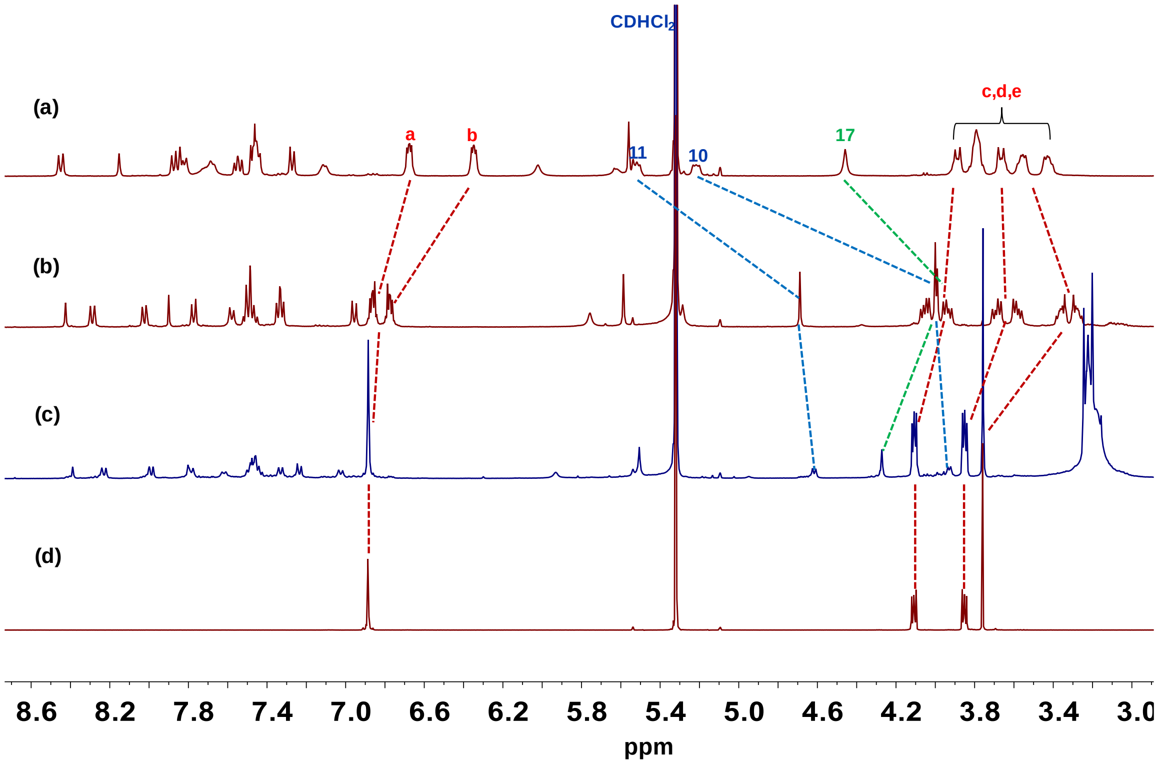


**Figure S11**. $^1$H NMR ($CD_2Cl_2$, 400 MHz, 298K) comparison spectra of (a) **R2-H$^{2+}$**, (b) **R-2$^+$**, (c) **3$^+$** + **1**, (d) free **1.**

## 5. Synthesis of compound 2$^+$ (*ex-situ*)

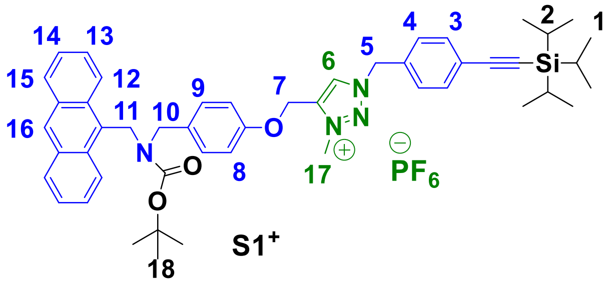

**S1$^+$**

Alkyne **4** (600 mg, 1.71 mmol, 1 eq.) and azide **3** (541 mg, 1.72 mmol, 1.01 eq.) were dissolved in dichloromethane (15 mL) and the solution was degassed by purging with argon for five minutes. Then tetrakis(acetonitrile)copper(I) hexafluorophosphate (636 mg, 1.71 mmol, 1.eq.) was added and the mixture was stirred for 16 hours at room temperature und argon atmosphere. The solution was washed with a saturated solution of ethylenediaminetetraacetic acid disodium salt (3 x 15 mL). The organic phase was dried over sodium sulfate, filtered and concentrated under vacuum. The brown residue (1.18 g) was dissolved in a mixture of ethanol and dichloromethane (3/5 v/v, 8 mL) and Amberlyst 15 (240 mg) was added. Di-*tert*-butyl-

dicarbonate (426 mg, 1.95 mmol, 1.1 eq.) was added and the mixture was stirred for 8 hours at room temperature. The mixture was filtered, volatiles were removed *in vacuo* and the crude product was dissolved in acetonitrile (15 mL). Methyl iodide (1 mL, 16.3 mmol, 10 eq.) was added and the mixture was heated to reflux under argon atmosphere for 12 hours. Volatiles were removed under reduced pressure and the residue was dissolved in dichloromethane. Ammonium hexafluorophosphate (1.33 g, 8.17 mmol, 5 eq.) was added and the suspension was stirred for 5 hours at room temperature. The mixture was filtered and concentrated under reduced pressure. After purification by flash column chromatography on silica using a gradient of diethyl ether/acetone (from 100/0 to 10/90). The desired product **S1**$^{+}$ (as the $PF_6^{-}$-salt, 876 mg, 947 µmol, 55% yield over 3 steps) was isolated as a yellow foamy solid.

Some signals in the $^{1}$H- and $^{13}$C-NMR spectra appear broad, probably due to slowly equilibrating rotamers caused by the *N*-Boc-group.

**$C_{49}H_{59}F_6N_4O_3PSi$**: 925.0852 g/mol

**$^{1}$H-NMR (400 MHz, $CD_2Cl_2$, 298 K)**: δ = 8.91 (s, 1H, 6-H), 8.45 (s, 1H, 16-H), 8.25 – 8.17 (m, 2H, 12-H), 8.08 – 7.96 (m, 2H, 15-H), 7.57 – 7.48 (m, 4H, 3,4-H), 7.48 – 7.43 (m, 4H, 13,14-H), 6.77 (br s, 4H, 8,9-H), 5.84 (s, 2H, 5-H), 5.52 (s, 2H, 11-H), 5.30 (s, 2H, 7-H), 4.36 (s, 3H, 17-H), 3.98 (s, 2H, 10-H), 1.52 (br s, 9H, 18-H), 1.15 – 1.10 (m, 21H, 1,2-H) ppm.

**$^{13}$C NMR (101 MHz, $CD_2Cl_2$, 298 K)**: δ = 155.5 (overlapping signals, $C_{\text{Quarternary-phenyl-C-O}}$, $C_{\text{carbonyl}}$), 139.8 ($C_{\text{Quarternary-triazolium}}$), 133.0 ($C_{\text{Quarternary-phenyl-CH2-N}}$), 132.9 (3-C) 131.33, 131.26 (overlapping signals, 2 x $C_{\text{Quarternary-anthracene}}$), 130.5 ($C_{\text{Quarternary-phenyl-CH2-triazolium}}$), 130.0 (6-C), 129.5 (4-C), 129.0 (15-C), 128.4 (overlapping signals, 9-C, $C_{\text{Quarternary-anthracene}}$), 128.1 (16-C), 126.1 (13-C), 125.6 ($C_{\text{Quarternary-phenyl-ethynyl}}$), 125.0 (14-C), 124.3 (12-C), 114.2 (8-C), 105.6 ($C_{\text{Quarternary-ethynyl-phenyl}}$), 93.2 ($C_{\text{Quarternary-ethynyl-Si}}$), 80.1 ($C_{\text{Quarternary-}\textit{tert}\text{-butyl}}$), 58.3 (7-C), 57.4 (5-C), 47.0 (10-C), 40.8 (11-C, very broad, detected by HSQC), 39.2 (17-C), 28.2 (18-C), 18.3 (1-C), 11.2 (2-C) ppm.

**COSY (400 MHz/400 MHz, $CD_2Cl_2$, 298 K):** δ ($^{1}$H) / δ ($^{1}$H) = 8.25 – 8.17/7.48 – 7.43, (12-H/13-H), 8.08 – 7.96/7.48 – 7.43 (15-H/14-H), 7.48 – 7.43/8.25 – 8.17, 8.08 – 7.96 (13-H/12-H, 14-H/15-H).

**HSQC (400 MHz/101 MHz, $CD_2Cl_2$, 298 K):** δ ($^{1}$H) / δ ($^{13}$C) = 8.91/130.0 (6-H/6-C), 8.45/128.1 (16-H/16-C), 8.25 – 8.17/124.3 (12-H/12-C), 8.08 – 7.96/129.0 (15-H/15-C), 7.57 – 7.48/132.9, 129.5 (H-3/C-3, H-4/C-4), 7.48 – 7.43/126.1, 125.0 (13-H/13-C, 14-H/14-C),

6.77/128.4, 114.2 (8,9-H/8,9-C), 5.84/57.4 (5-H/5-C), 5.52/40.8 (11-H/11-C), 5.30/58.3 (7-H/7-C), 4.36/39.2 (17-H/17-C), 3.98/47.0 (10-H/10-C), 1.52/28.2 (17-H/17-C), 1.15 – 1.10/18.3, 11.2 (1-H/1-C, 2H/2-C).

**HMBC (400 MHz/101 MHz, $CD_2Cl_2$, 298 K):** δ ($^{1}H$) / δ ($^{13}C$) = 8.91/139.8 (6-H/$C_{Quarternary-triazolium}$), 8.45/131.33, 131,26, 129.0 (16-H/$C_{Quarternary-anthracene}$, $C_{Quarternary-anthracene}$, 15-C), 8.25 – 8.17/131.33, 131.26, 125.0 (12-H/$C_{Quarternary-anthracene}$, $C_{Quarternary-anthracene}$, 14-C), 8.08 – 7.96/131.33, 131.26, 128.1, 126.1 (15-H/ $C_{Quarternary-anthracene}$, $C_{Quarternary-anthracene}$, 16-C, 13-C), 7.57 – 7.48/132.9, 130.5, 129.5, 125.6, 105.6, 57.4 (4-H/C-3, 3-H/$C_{Quarternary-CH2-triazolium}$, 4-C, 4-H/$C_{Quarternary-phenyl-ethynyl}$, 3-H/ $C_{Quarternary-ethynyl-phenyl}$, 4-H/5-C), 7.48 – 7.43/131.33, 131.26, 129.0, 124.3 (13,14-H/ $C_{Quarternary-anthracene}$, 15-C, 14-C), 5.84/130.5, 129.5 (5-H/$C_{Quarternary-phenyl-CH2-triazolium}$, 4-C), 5.52/155.5, 131.33, 131.26, 128.4 (10-H/$C_{Carbonyl}$, $C_{Quarternary-Ph-O}$, 9-C), 5.30/155.3, 139.8, 130.0 (7-H/$C_{Quarternary-phenyl-C-O}$, $C_{Quarternary-triazolium}$, 6-C), 4.36/139.8 (17-H/$C_{Quarternary-triazolium}$), 3.98/155.3, 133.0, 128.4 ($C_{Carbonyl}$, $C_{Quarternary-phenyl-CH2-N}$, 9-C), 1.15 – 1.10/18.3, 11.2 (1-H/2-C, 2H/1-C).

**$^{19}$F NMR (376 MHz, $CD_2Cl_2$, 298 K)** δ = -73.15 (d, *J* = 711 Hz, P*F*$_6^-$ ) ppm.

**$^{31}$P NMR (162 MHz, $CD_2Cl_2$, 298 K)** δ = -144.6 (sept, *J* = 711 Hz, *P*F$_6^-$ ) ppm.

**IR (ATR-FT):** $\tilde{\nu}$ = 3142, 2981, 2970, 2943, 2891, 2864, 1684, 1508, 1457, 1450, 1365, 1237, 1158, 1115, 831, 790, 736, 677, 659, 557 cm$^{-1}$.

**HR-MS** (ESI-pos, MeOH): m/z = 779.4358 ([**S1**]$^+$, calcd. 779.4351 for [$C_{49}H_{59}O_3N_4Si$]$^+$).

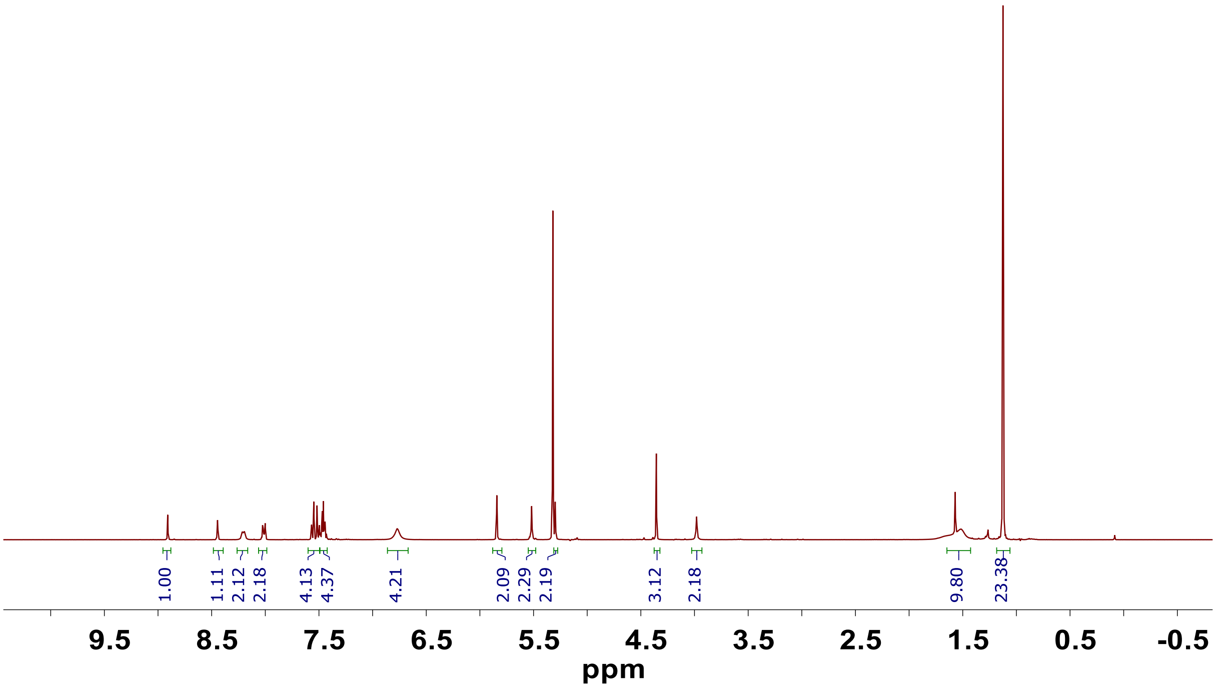


**Figure S12.** $^{1}$H NMR spectrum ($CD_2Cl_2$, 400 MHz, 298K) of **S1**$^{+}$ (as the $PF_6^{-}$-salt).

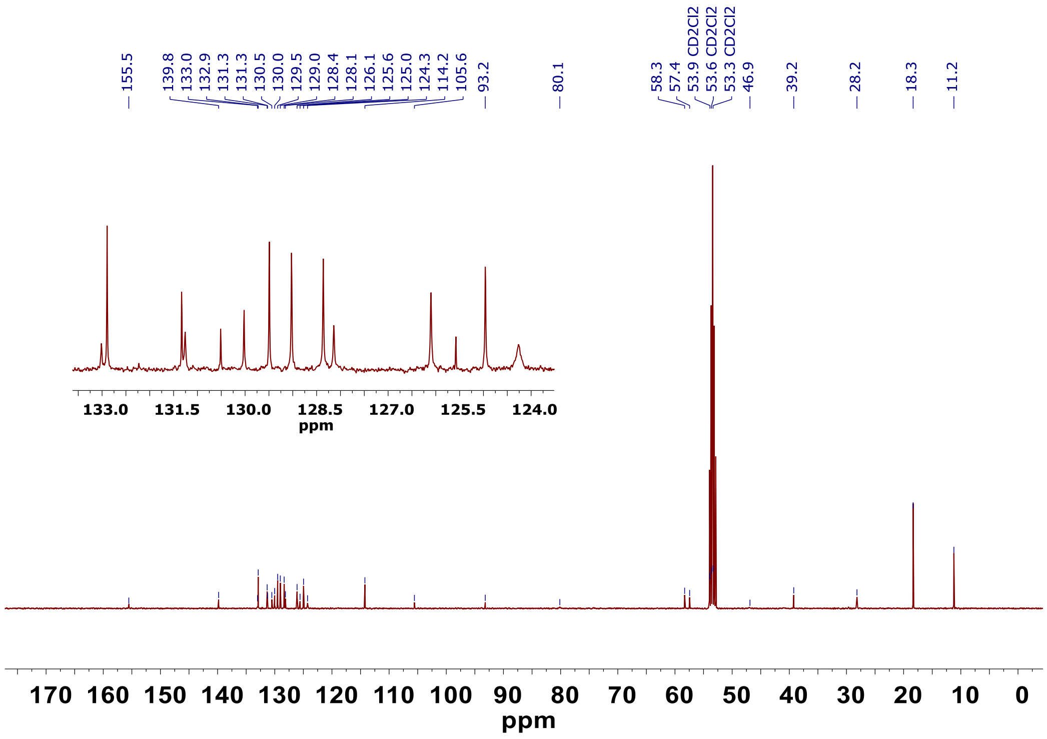


**Figure S13.** $^{13}$C NMR spectrum ($CD_2Cl_2$, 101 MHz, 298 K) of **S1**$^{+}$ (as the $PF_6^{-}$-salt).

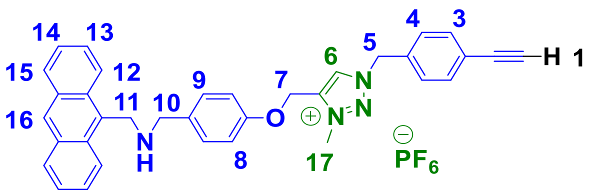


**2⁺**

**S1⁺** (100 mg, 108 µmol, 1eq.) was dissolved in tetrahydrofuran (2 mL) and tetrabutylammonium fluoride trihydrate (68.2 mg, 216 µmol, 2 eq.) was added. The mixture was stirred for 10 minutes at room temperature. Then, aqueous hexafluorophosphoric acid (55%, 87.0 µL, 540 µmol, 5 eq.) was added and the mixture was stirred at room temperature for 2 hours. The solution was diluted with dichloromethane (10 mL) and aqueous sodium hydroxide (2 M, 10 mL). Phases were separated and the aqueous phase was extracted with dichloromethane (3 x 10 mL). The combined organic phases were dried over sodium sulfate, filtered and volatiles were removed under reduced pressure to yield the desired compound as a yellow solid (160 mg) as an admixture with two equivalents of a tetrabutylammonium salt, which could not be removed with either column chromatography on silica (eluent: diethyl ether/acetone 8/2 or dichloromethane/methanol 95/5) or liquid/liquid extractions (dichloromethane/brine or dichloromethane/hydrochloric acid (2 M)).

**$C_{35}H_{31}F_6N_4OP$**: 668.6242 g/mol

**$^1$H-NMR (400 MHz, $CD_2Cl_2$, 298 K)**: δ = 8.41 (s, 1H, 16-H), 8.38 (s, 1H, 6-H), 8.25 (d, *J* = 9.3 Hz, 2H, 12-H), 8.00 (d, *J* = 7.4 Hz, 2H, 15-H), 7.57 – 7.34 (m, 10H, 3,4,9,13,14-H), 6.97 (d, *J* = 8.7 Hz, 2H, 8-H), 5.67 (s, 2H, 5-H), 5.25 (s, 2H, 7-H), 4.66 (s, 2H, 11-H), 4.30 (s, 3H, 17-H), 3.98 (s, 2H, 10-H), 3.22 (s, 1H, 1-H) ppm.

**$^{13}$C NMR (101 MHz, $CD_2Cl_2$, 298 K)**: δ = 156.5 ($C_{Quarternary\text{-}phenyl\text{-}C\text{-}O}$), 140.7 ($C_{Quarternary\text{-}triazolium}$), 135.3 ($C_{Quarternary\text{-}phenyl\text{-}CH2\text{-}N}$), 134.7 ($C_{Ar,\ could\ not\ be\ assigned}$), 133.6 (3-C), 132.0 ($C_{Quarternary\text{-}anthracene}$), 131.7 ($C_{Quarternary\text{-}phenyl\text{-}CH2\text{-}triazolium}$), 130.8 ($C_{Quarternary\text{-}anthracene}$), 130.4 (9-C), 129.9 (overlapping signals, 4,6-C), 129.5 (15-C), 127.7 (16-C), 127.5 ($C_{Quarternary\text{-}anthracene\text{-}CH2}$), 126.6 (16-C), 125.6 (14-C), 124.8 (12-C), 124.6 ($C_{Quarternary\text{-}phenyl\text{-}ethynyl}$), 115.1 (8-C), 82.8 ($C_{Quarternary\text{-}ethynyl\text{-}phenyl}$), 79.5 ($C_{ethynyl\text{-}H}$), 59.2 ($C_{tetrabutylammonium}$), 58.5 (7-C), 57.8 (5-C), 54.0 (10-C, overlapping with the solvent signal, assigned by HSQC and HMBC), 45.5 (11-C), 39.4 (17-C), 24.18, 20.09, 13.77 (3 x $C_{tetrabutylammonium}$) ppm.

**COSY (400 MHz/400 MHz, $CD_2Cl_2$, 298 K):** δ ($^1$H) / δ ($^1$H) = 8.25/7.57 – 7.34 (12-H/13-H), 8.00/7.57 – 7.34 (15-H/14-H), 7.57 – 7.34/8.25, 8.00, 6.97 (13-H/12-H, 14-H/15-H, 9-H/8-H), 6.97/7.57 – 7.34 (8-H/9-H).

**HSQC (400 MHz/101 MHz, $CD_2Cl_2$, 298 K):** δ ($^1$H) / δ ($^{13}$C) = 8.41/127.7 (16-H/16-C), 8.38/129.9 (6-H/6-C), 8.25/124.8 (12-H/12-C), 8.00/129.5 (H-15/C-15), 7.57 – 7.34/133.6, 130.4, 129.9, 126.6, 125.6 (3-H/3-C, 9-H/9-C, 4-H/4-C, 13-H/13-C, 14-H/14-C), 6.97/115.1 (8-H/8-C), 5.67/57.8 (5-H/5-C), 5.25/58.5 (7-H/7-C), 4.66/45.5 (11-H/11-C), 4.30/39.4 (17-H/17-C), 3.98/54.0 (10-H/10-C).

**HMBC (400 MHz/101 MHz, $CD_2Cl_2$, 298 K):** δ ($^1$H) / δ ($^{13}$C) = 8.41/130.8, 129.5 (16-H/ $C_{Quarternary\text{-}anthracene}$, 15-C), 8.38/140.7 (6-H/$C_{Quarternary\text{-}triazolium}$), 8.25/132.0, 125.6 (12-H/ $C_{Quarternary\text{-}anthracene}$, 14-C), 8.00/132.0, 130.8, 127.7, 127.5, 126.6 (15-H/$C_{Quarternary\text{-}anthracene}$, 16-C, $C_{Quarternary\text{-}anthracene\text{-}CH2}$, 13-C), 7.57 – 7.34/156.5, 133.6, 132.0, 131.7, 130.8, 129.5, 124.6, 124.8, 82.8, 57.8, 54.0 (9-H/$C_{Quarternary\text{-}phenyl\text{-}C\text{-}O}$, 4-H/3-C, 13,14-H/$C_{Quarternary\text{-}anthracene}$, 4-H/$C_{Quarternary\text{-}phenyl\text{-}CH2\text{-}triazolium}$, 13,14-H/$C_{Quarternary\text{-}anthracene}$, 13-H/15-C, 14-H/12-C, 4-H/$C_{Quarternary\text{-}phenyl\text{-}ethynyl}$, 3-H/$C_{Quarternary\text{-}ethynyl\text{-}phenyl}$, 4-H/5-C, 9-H/10-C), 6.97/156.5, 135.3 (8-H/$C_{Quarternary\text{-}phenyl\text{-}C\text{-}O}$, $C_{Quarternary\text{-}phenyl\text{-}CH2\text{-}N}$), 5.67/131.7, 129.9 (5-H/$C_{Quarternary\text{-}phenyl\text{-}CH2\text{-}triazolium}$, 6-C), 5.25/156.5, 140.7 (7-H/$C_{Quarternary\text{-}phenyl\text{-}C\text{-}O}$, $C_{Quarternary\text{-}triazolium}$), 4.66/132.0, 130.8, 54.0 (11-H/$C_{Quarternary\text{-}anthracene}$, 10-C), 4.30/140.7 (17-H/$C_{Quarternary\text{-}triazolium}$), 3.98/135.3, 130.4, 45.5 (10-H/$C_{Quarternary\text{-}phenyl\text{-}C\text{-}O}$, 9-C, 11-C), 3.22/82.8, 79.5 (1-H/ $C_{Quarternary\text{-}ethynyl\text{-}phenyl}$, $C_{ethynyl\text{-}H}$).

**$^{19}$F NMR (376 MHz, $CD_2Cl_2$, 298 K)** δ = -72.6 (d, *J* = 711 Hz, P$F_6^-$ ) ppm.

**$^{31}$P NMR (162 MHz, $CD_2Cl_2$, 298 K)** δ = -142.3 (sept, *J* = 711 Hz, *P*$F_6^-$ ) ppm.

**IR (ATR-FT):** $\tilde{\nu}$ = 3283, 2965, 2936, 2879, 1474, 1453, 1386, 1235, 1110, 1065, 1034, 878, 829, 737, 555 $cm^{-1}$.

**HR-MS** (ESI-pos, MeOH): m/z = 523.2495 ([**2-PF$_6$**]$^+$, calcd. 523.2492 for [$C_{35}H_{31}N_4O$]$^+$).

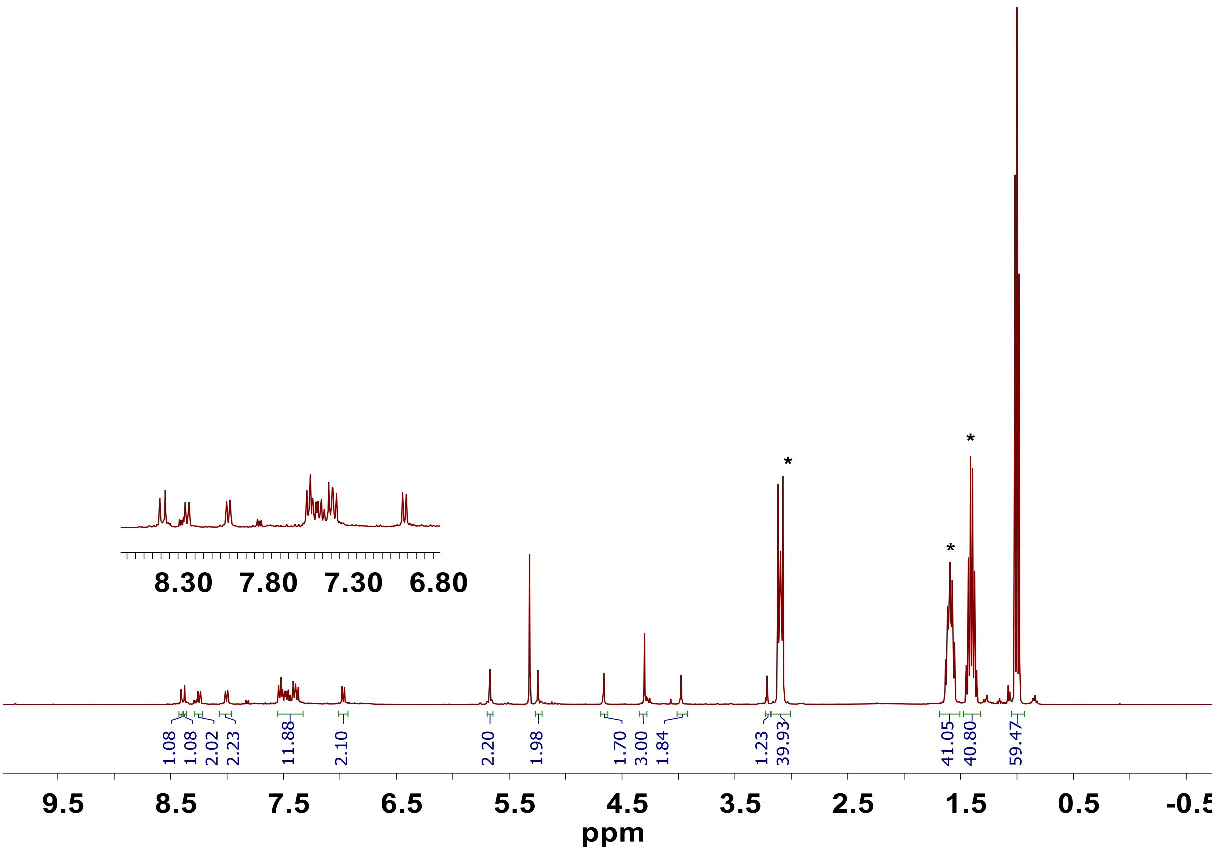


**Figure S14.** $^{1}H$ NMR spectrum ($CD_2Cl_2$, 400 MHz, 298K) of **2**$^{+}$ (as the $PF_6^{-}$-salt). * = Tetrabutylammonium-signals.

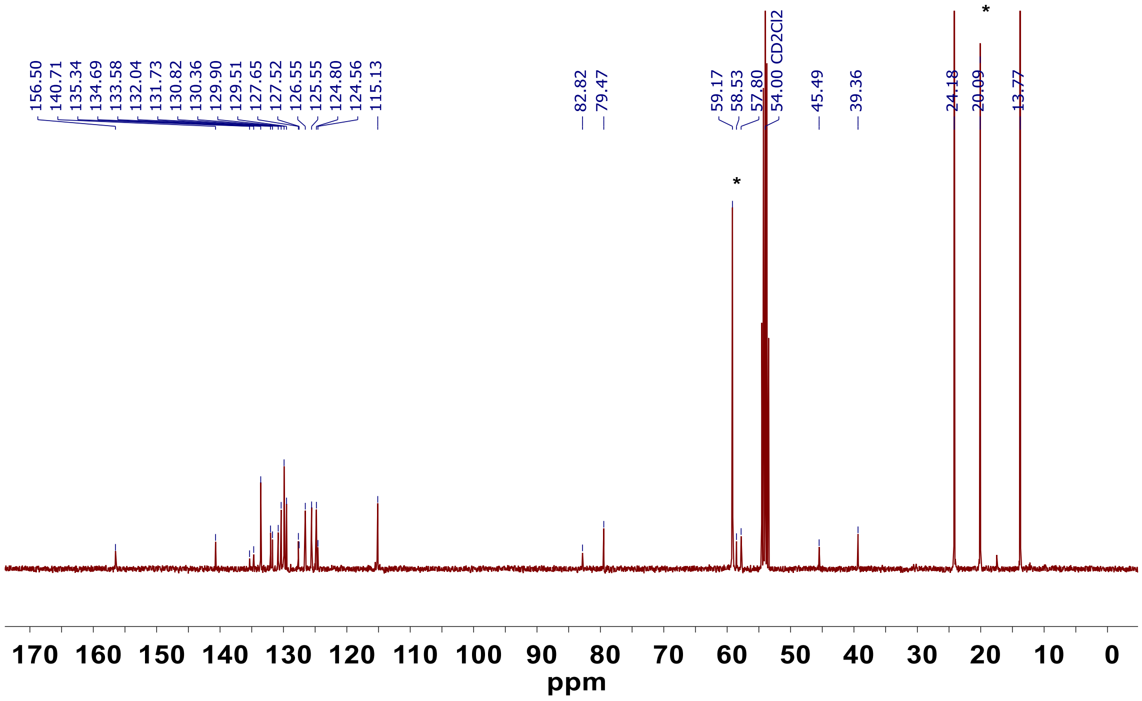


**Figure S15.** $^{13}C$ NMR spectrum ($CD_2Cl_2$, 101 MHz, 298K) of **2**$^{+}$ (as the bis-$PF_6^{-}$-salt). * = Tetrabutylammonium-signals.

## 6. 2D, 19F and 31P Spectra

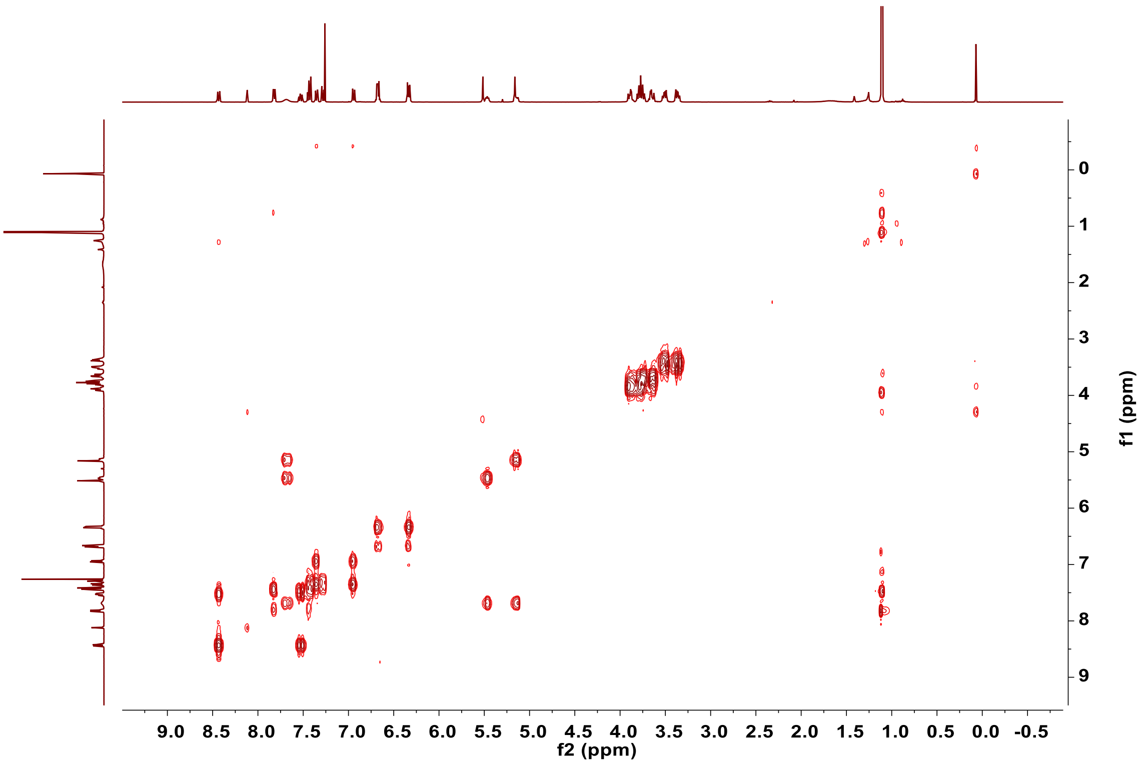


**Figure S16.** $^{1}H$-$^{1}H$ NMR COSY (400 MHz/400 MHz, $CD_2Cl_2$, 298 K) spectra of **R1a-H$^{+}$**.

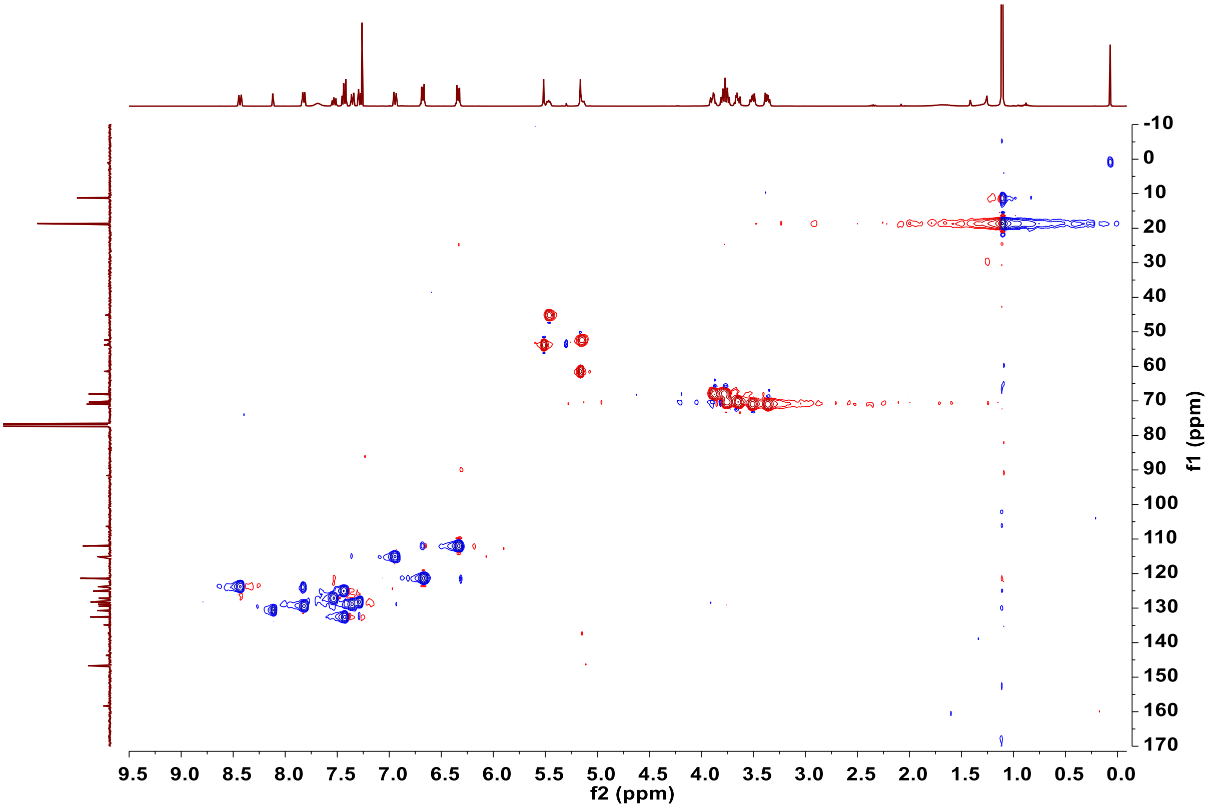


**Figure S17**. $^{1}H$-$^{13}C$ HSQC (400 MHz/101 MHz, $CD_2Cl_2$, 298 K) spectra of **R1a-H$^{+}$**.

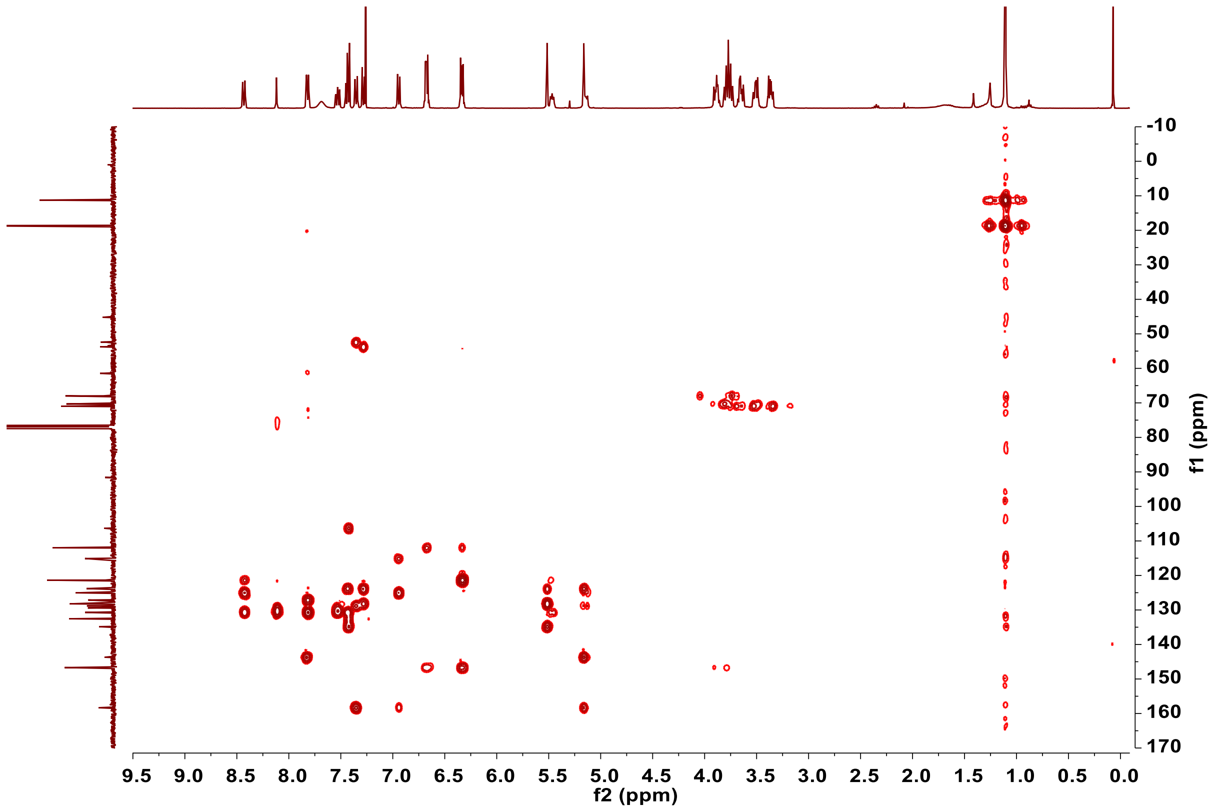


**Figure S18**. $^{1}H$-$^{13}C$ HMBC (400 MHz/101 MHz, $CD_2Cl_2$, 298 K) spectra of **R1a-H**$^{+}$.

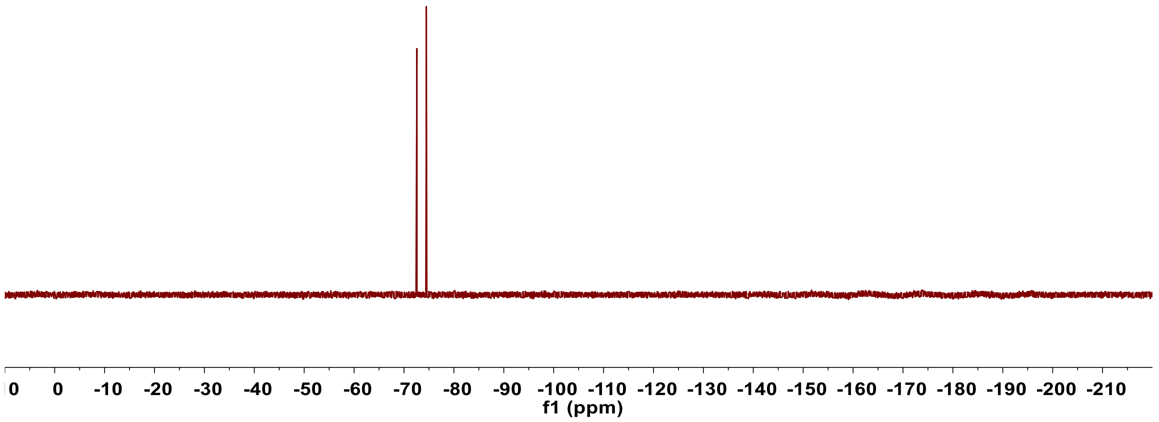


**Figure S19**. $^{19}F$ NMR (376 MHz, $CD_2Cl_2$, 298 K) specra of **R1a-H**$^{+}$.

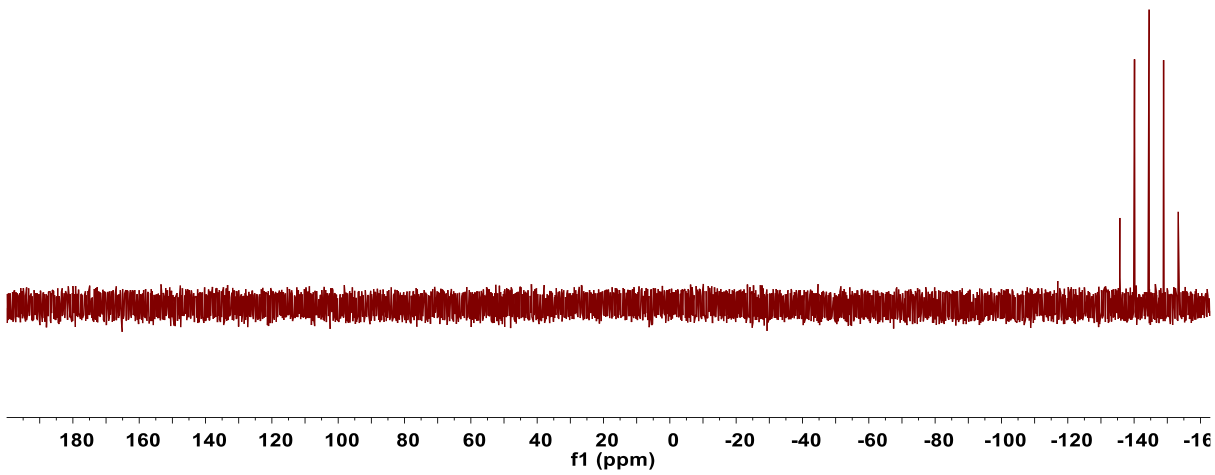


**Figure S20**. $^{31}$P NMR (162 MHz, $CD_2Cl_2$, 298 K) spectra of **R1a-H**$^{+}$.

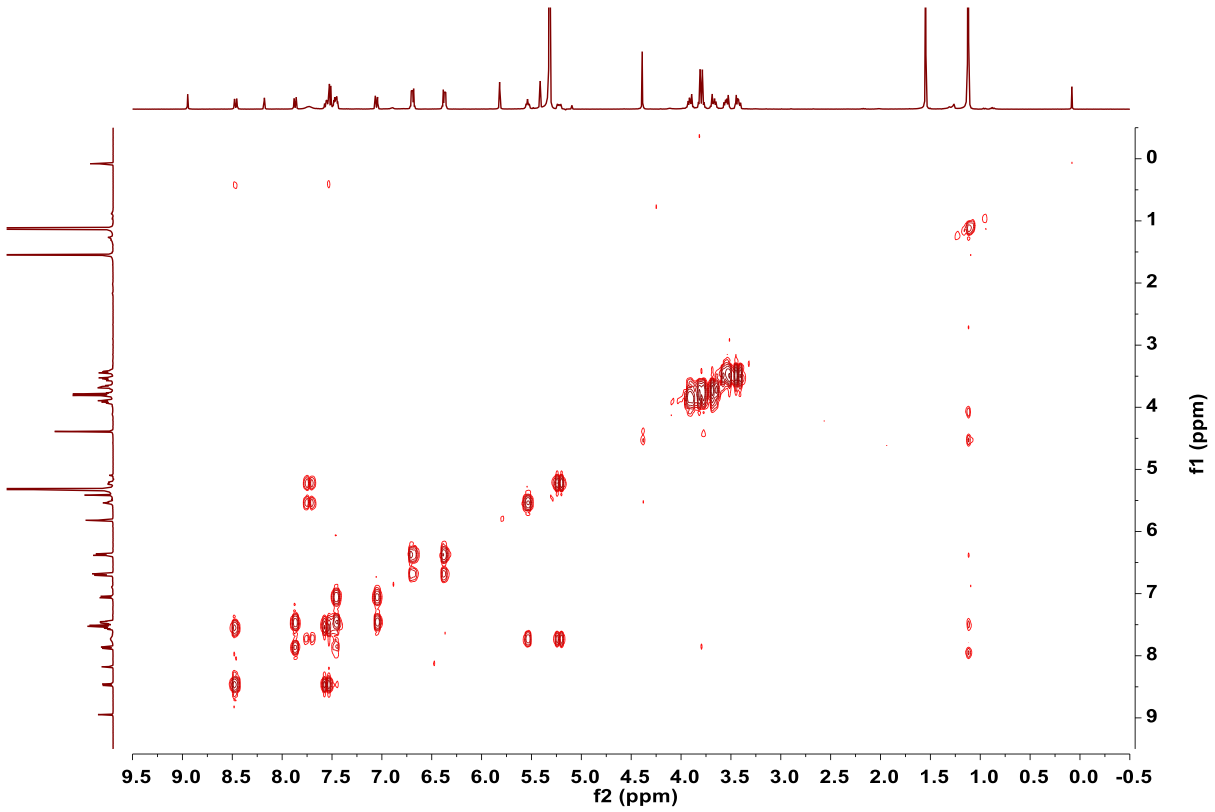


**Figure S21.** $^{1}$H-$^{1}$H NMR COSY (400 MHz/400 MHz, $CD_2Cl_2$, 298 K) spectra of **R1-H**$^{2+}$.

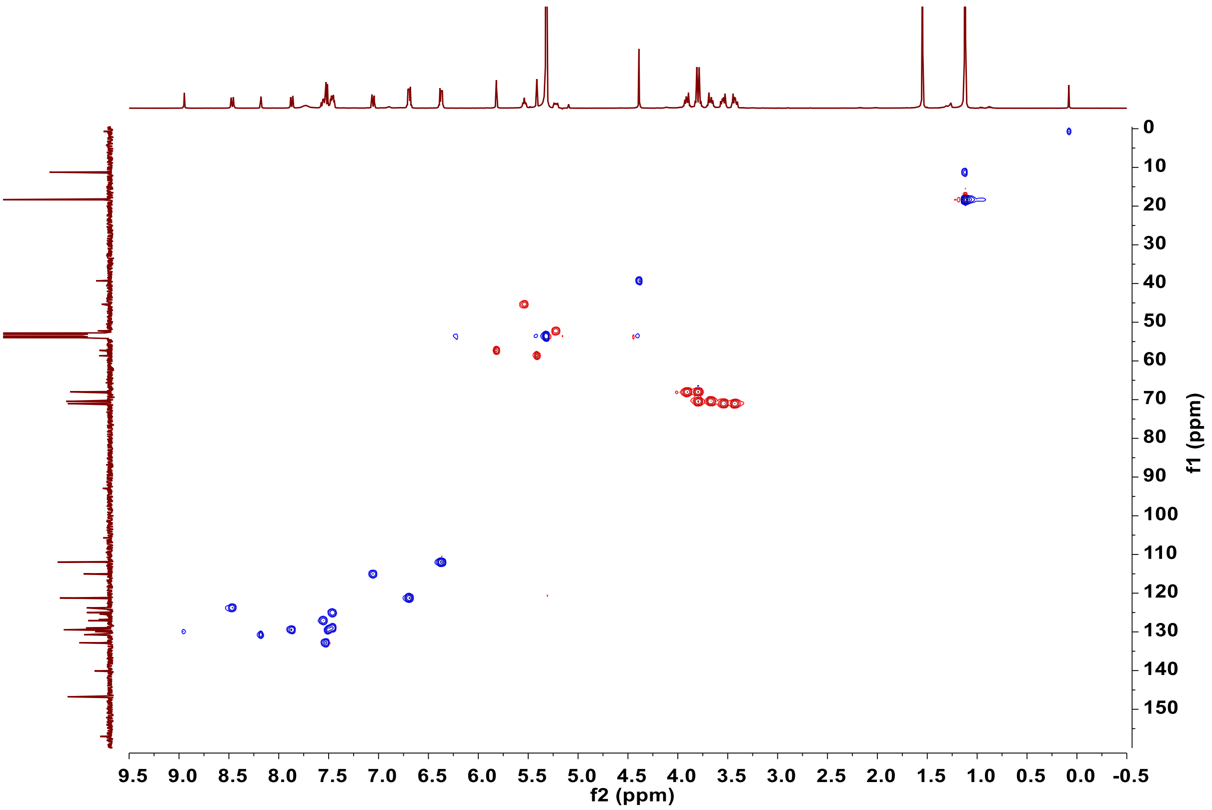


**Figure S22**. $^{1}$H-$^{13}$C HSQC (400 MHz/101 MHz, $CD_2Cl_2$, 298 K) spectra of **R1-H$^{2+}$**.

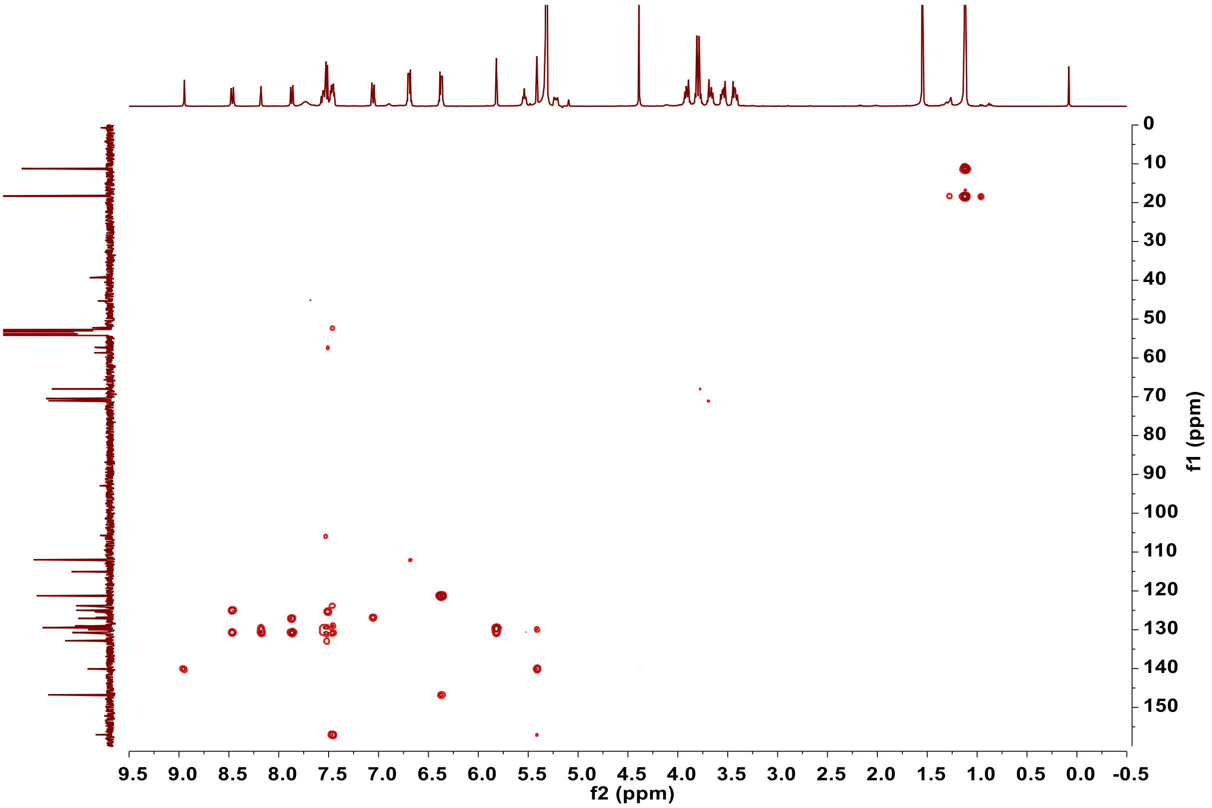


**Figure S23**. $^{1}$H-$^{13}$C HMBC (400 MHz/101 MHz, $CD_2Cl_2$, 298 K) spectra of **R1-H$^{2+}$**.

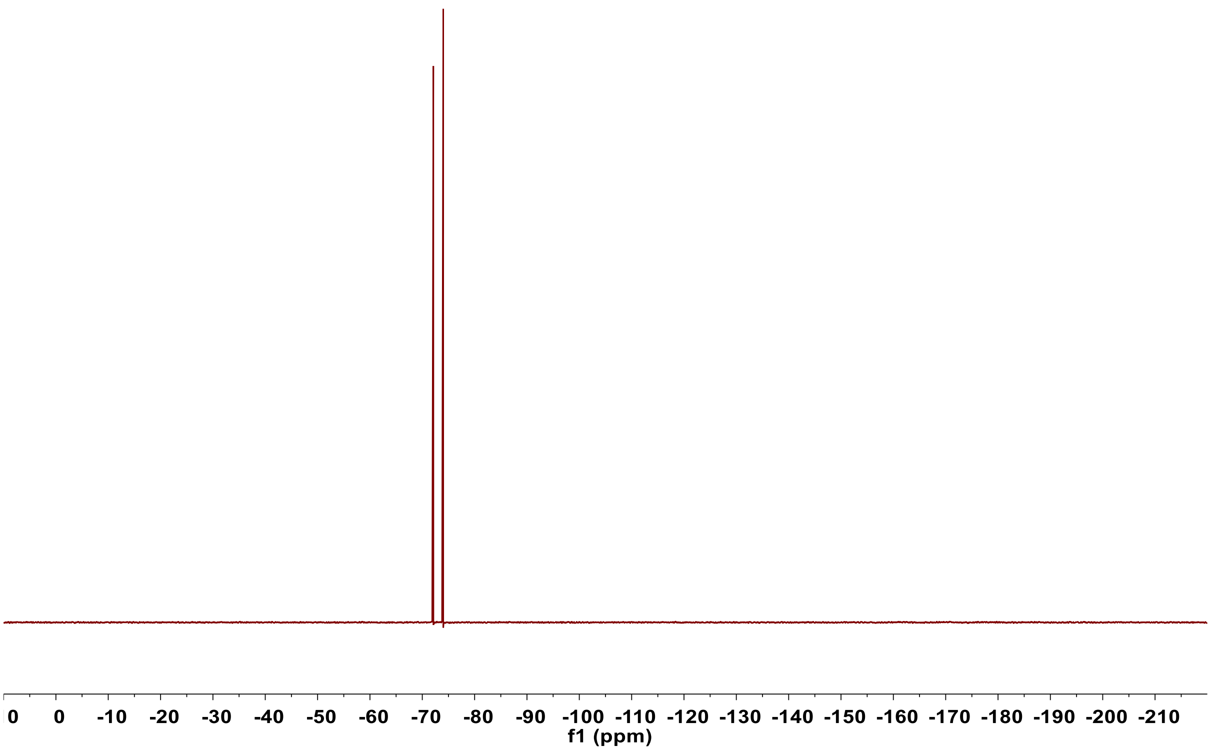


**Figure S24**. $^{19}$F NMR (376 MHz, $CD_2Cl_2$, 298 K) specra of **R1-H$^{2+}$**.

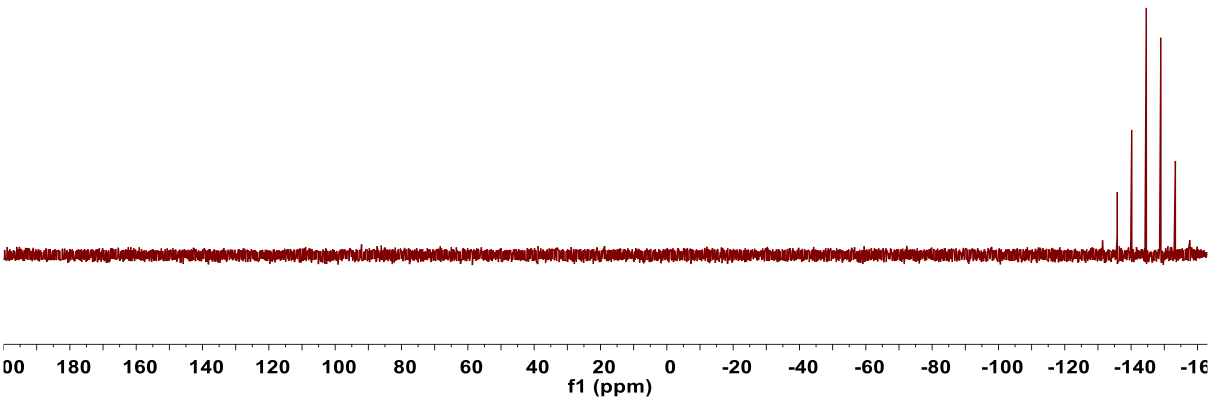


**Figure S25**. $^{31}$P NMR (162 MHz, $CD_2Cl_2$, 298 K) spectra of **R1-H$^{2+}$**.

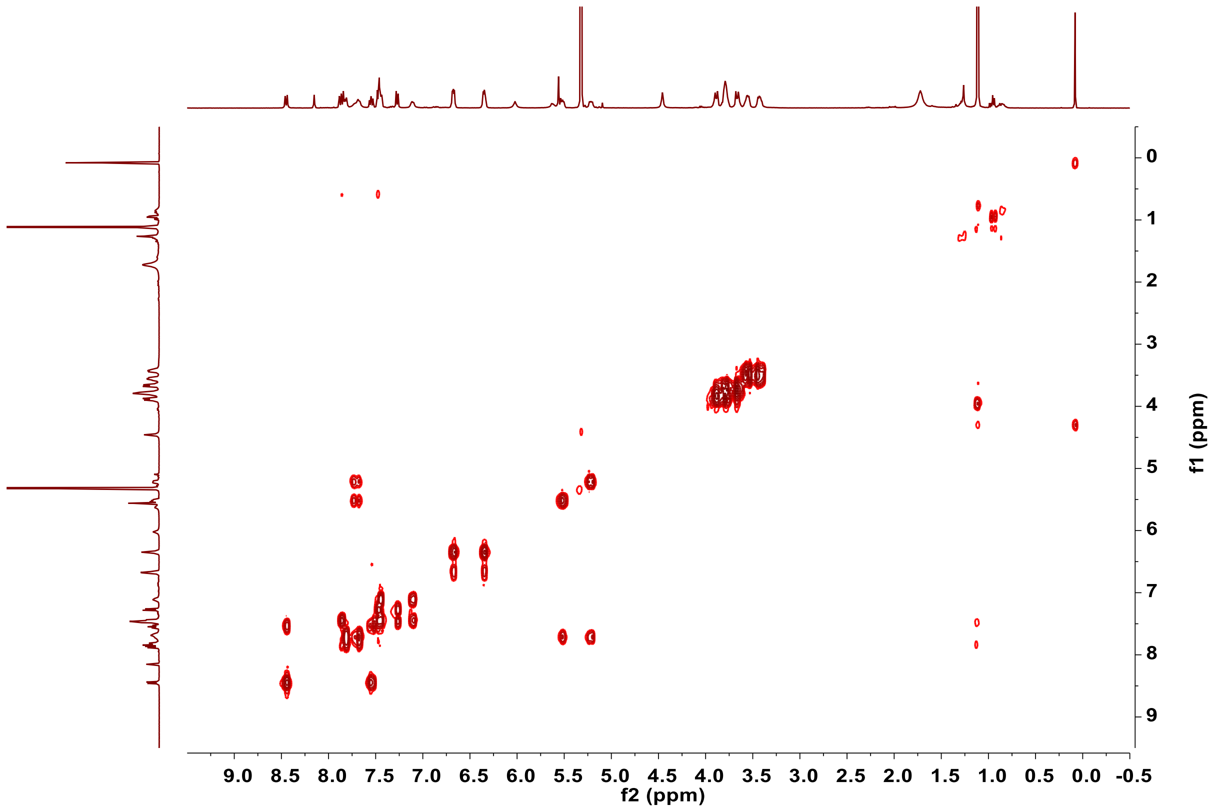


**Figure S26.** $^{1}H$-$^{1}H$ NMR COSY (400 MHz/400 MHz, $CD_2Cl_2$, 298 K) spectra of **R2-H$^{2+}$**.

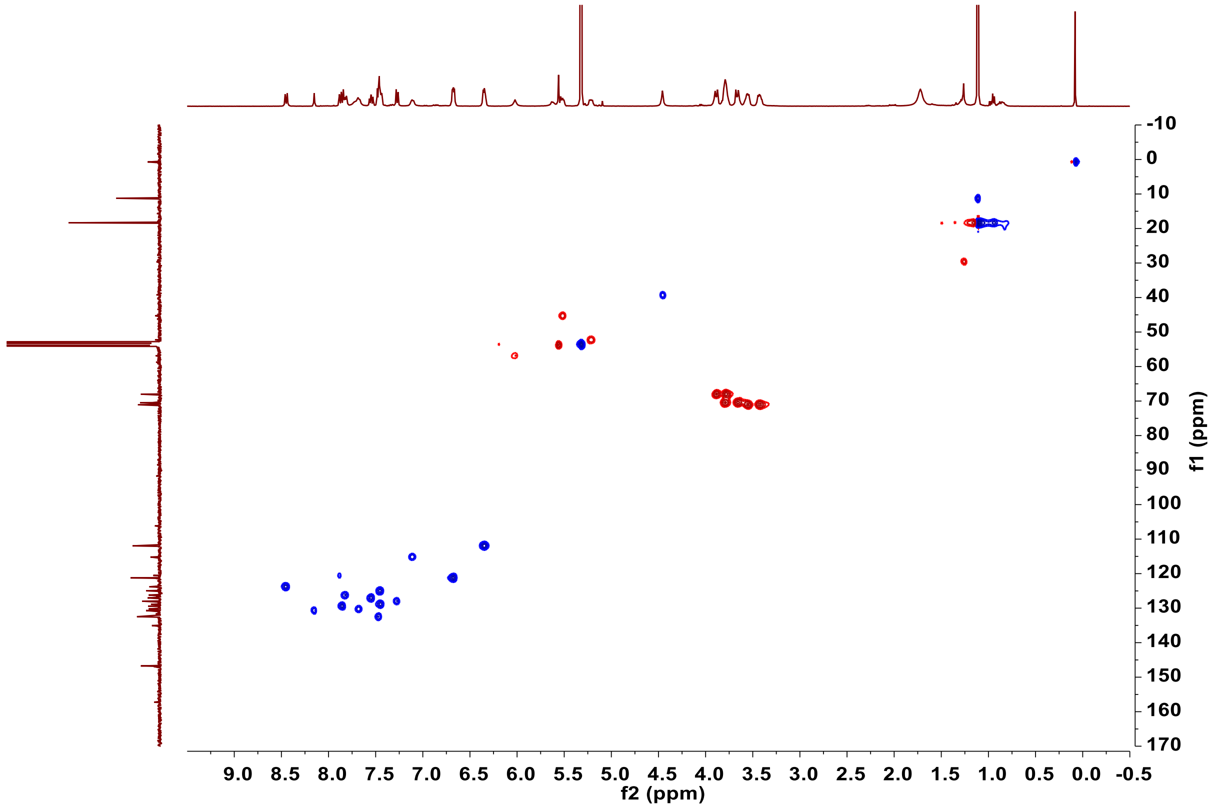


**Figure S27**. $^{1}H$-$^{13}C$ HSQC (400 MHz/101 MHz, $CD_2Cl_2$, 298 K) spectra of **R2-H$^{2+}$**.

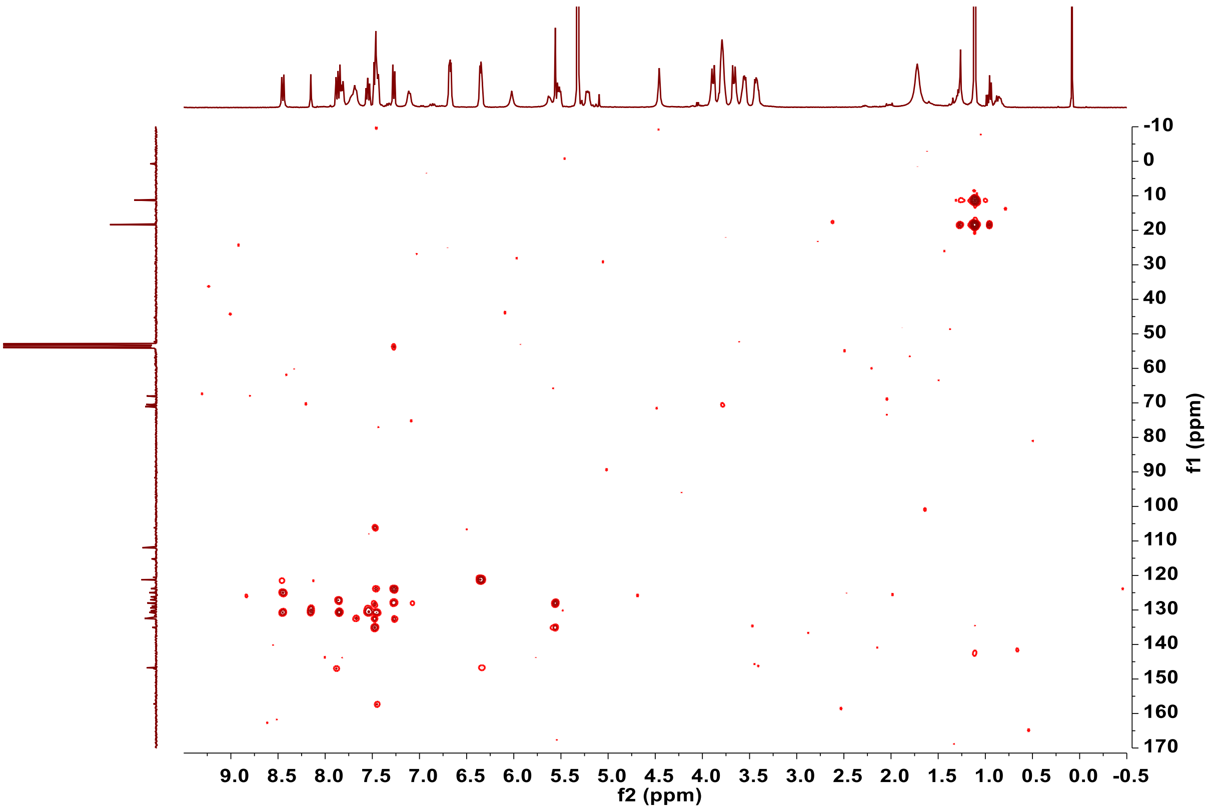


**Figure S28**. $^{1}H$-$^{13}C$ HMBC (400 MHz/101 MHz, $CD_2Cl_2$, 298 K) spectra of **R2-H$^{2+}$**.

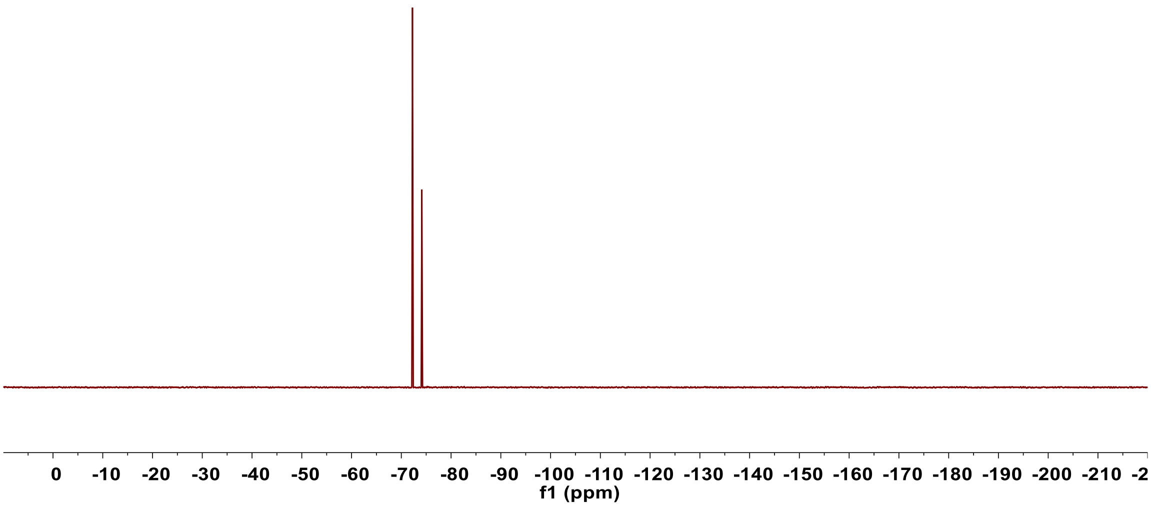


**Figure S29**. $^{19}F$ NMR (376 MHz, $CD_2Cl_2$, 298 K) specra of **R2-H$^{2+}$**.

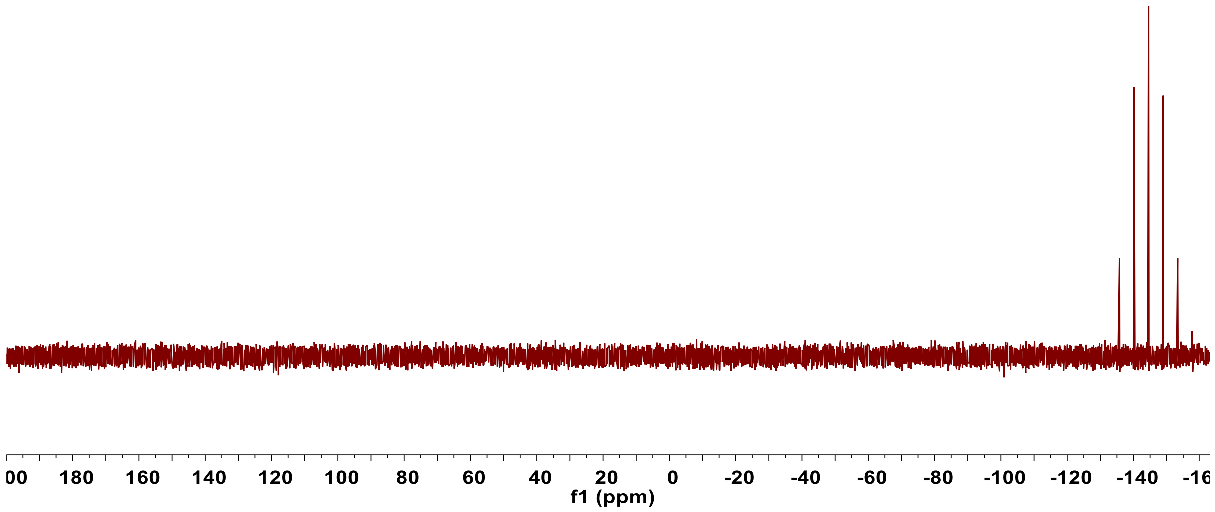


**Figure S30**. $^{31}$P NMR (162 MHz, $CD_2Cl_2$, 298 K) spectra of **R1-H$^{2+}$**.

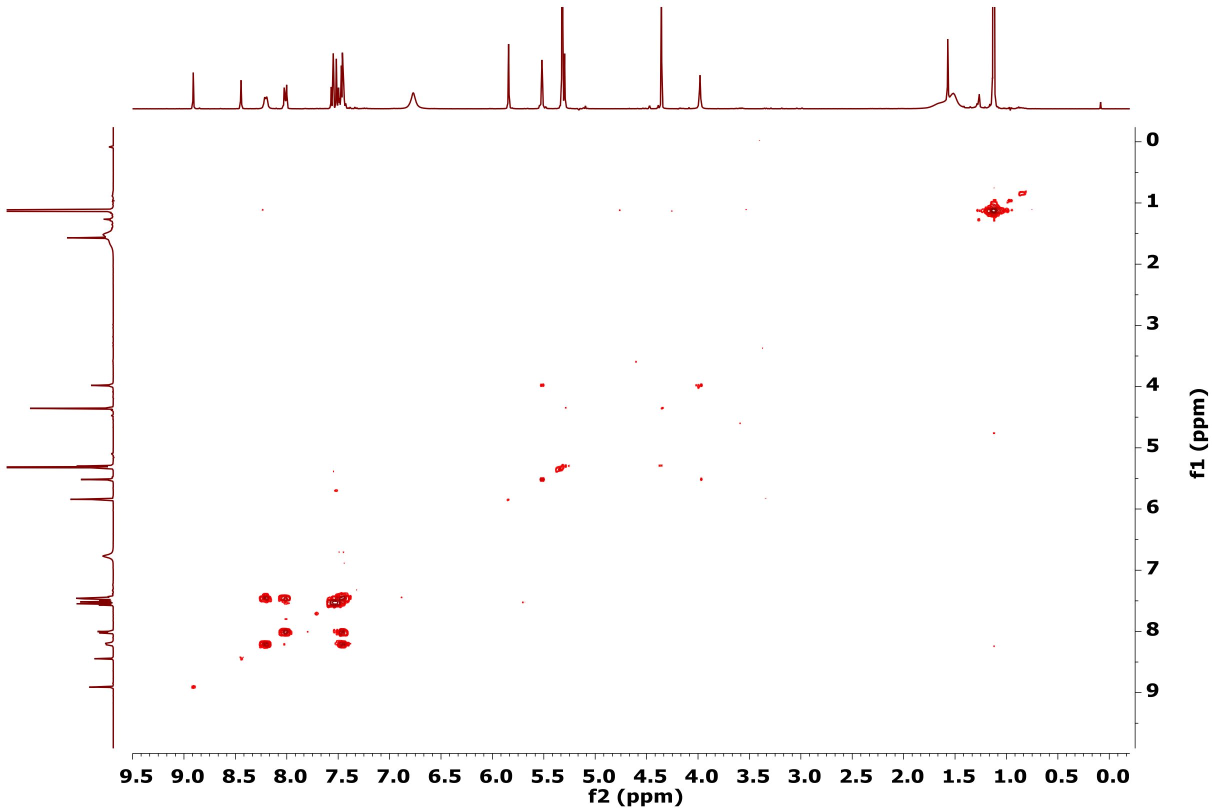


**Figure S31.** $^{1}$H-$^{1}$H NMR COSY (400 MHz/400 MHz, $CD_2Cl_2$, 298 K) spectra of **S1$^{+}$**.

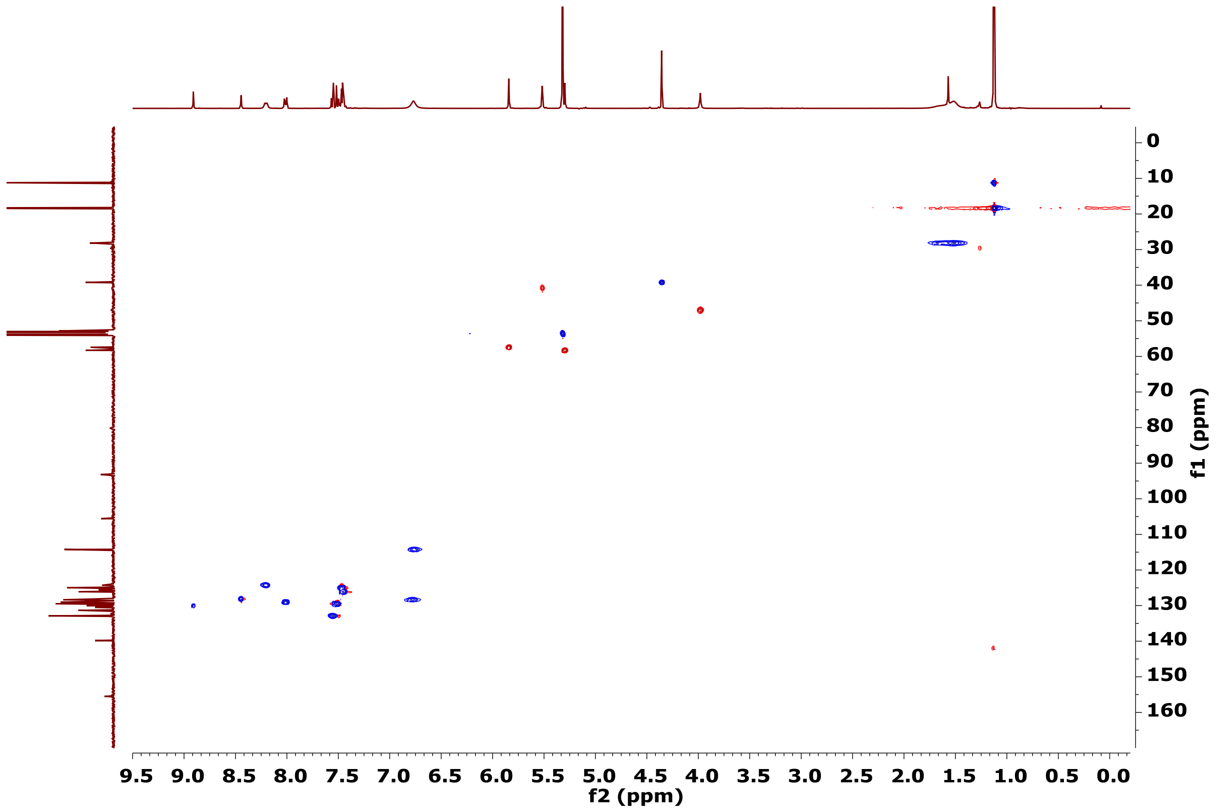


**Figure S32**. $^{1}H$-$^{13}C$ HSQC (400 MHz/101 MHz, $CD_2Cl_2$, 298 K) spectra of **S1**$^{+}$.

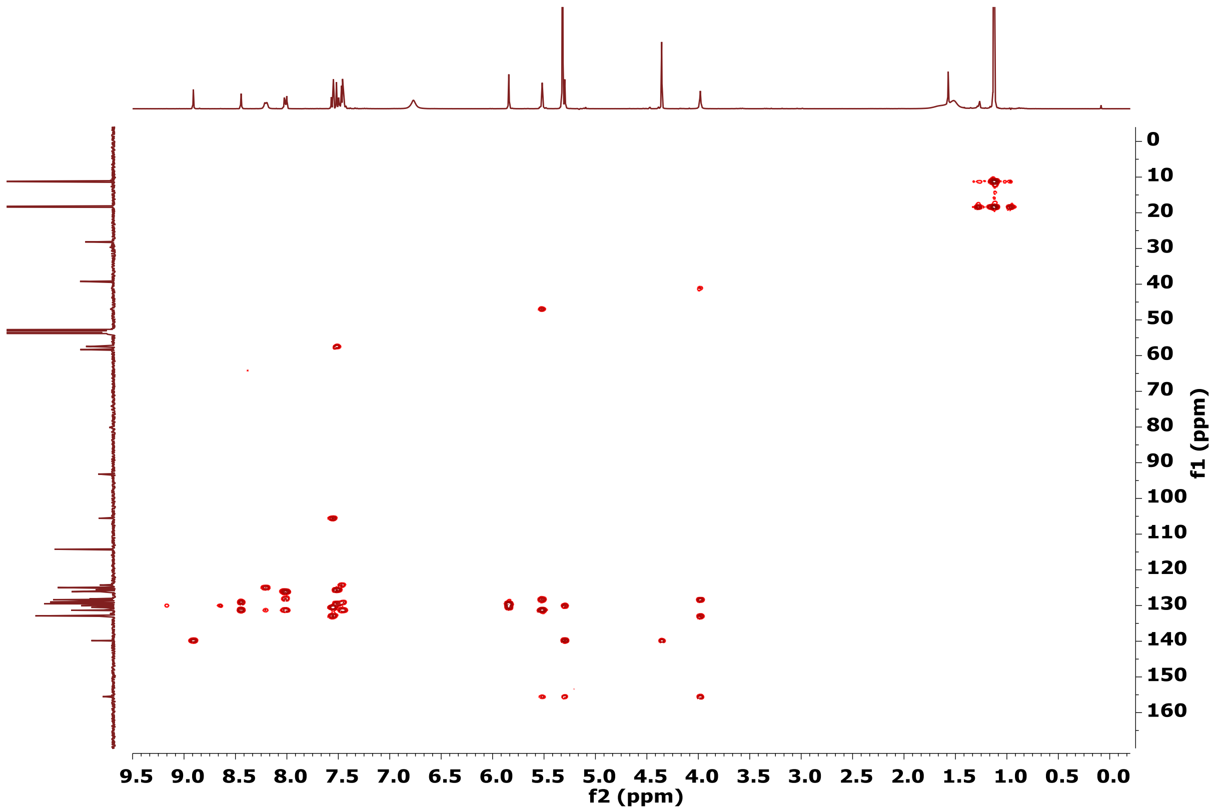


**Figure S33**. $^{1}H$-$^{13}C$ HMBC (400 MHz/101 MHz, $CD_2Cl_2$, 298 K) spectra of **S1**$^{+}$.

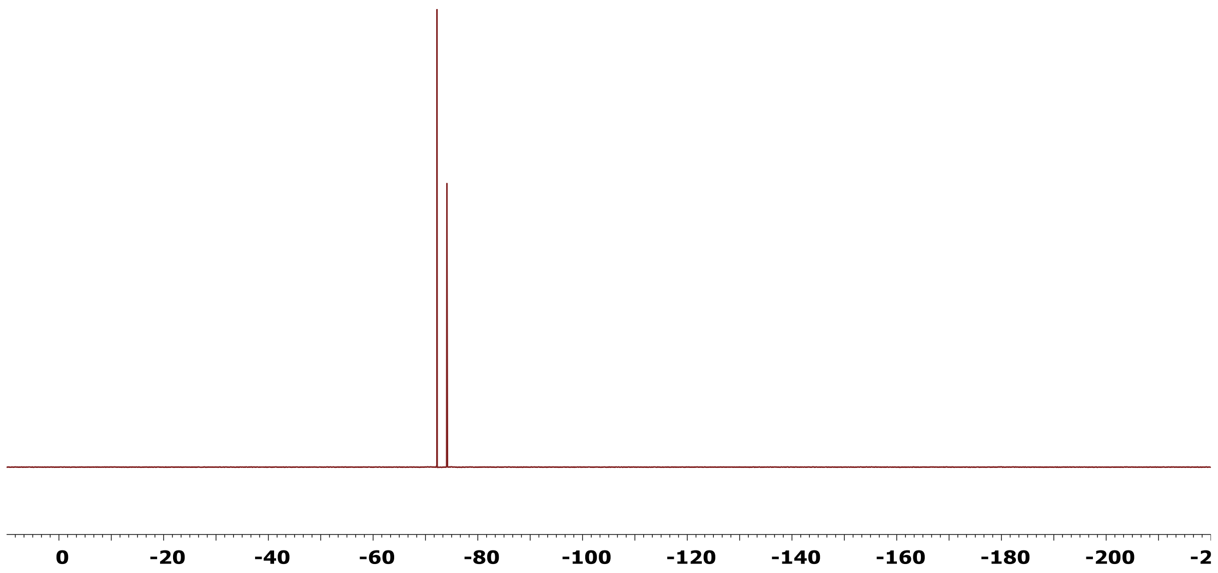


**Figure S34**. $^{19}$F NMR (376 MHz, $CD_2Cl_2$, 298 K) specra of **S1**$^+$.

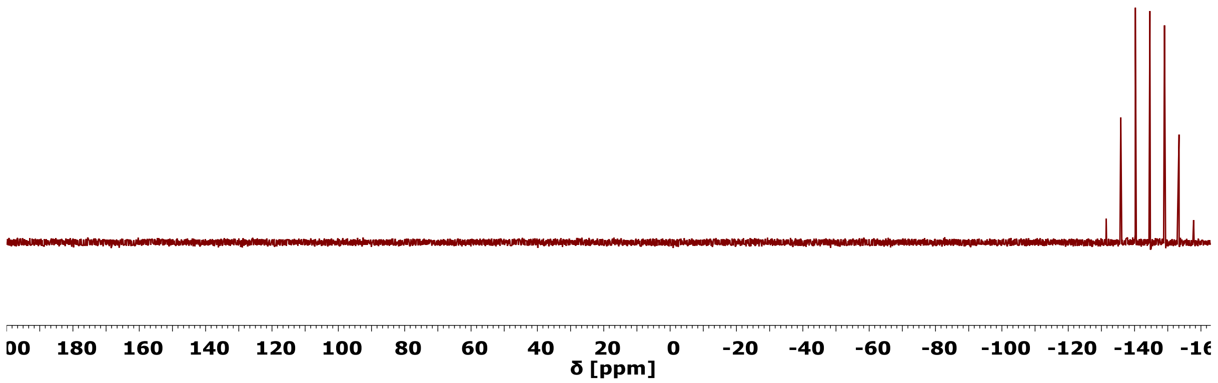


**Figure S35**. $^{31}$P NMR (162 MHz, $CD_2Cl_2$, 298 K) spectra of **S1**$^+$.

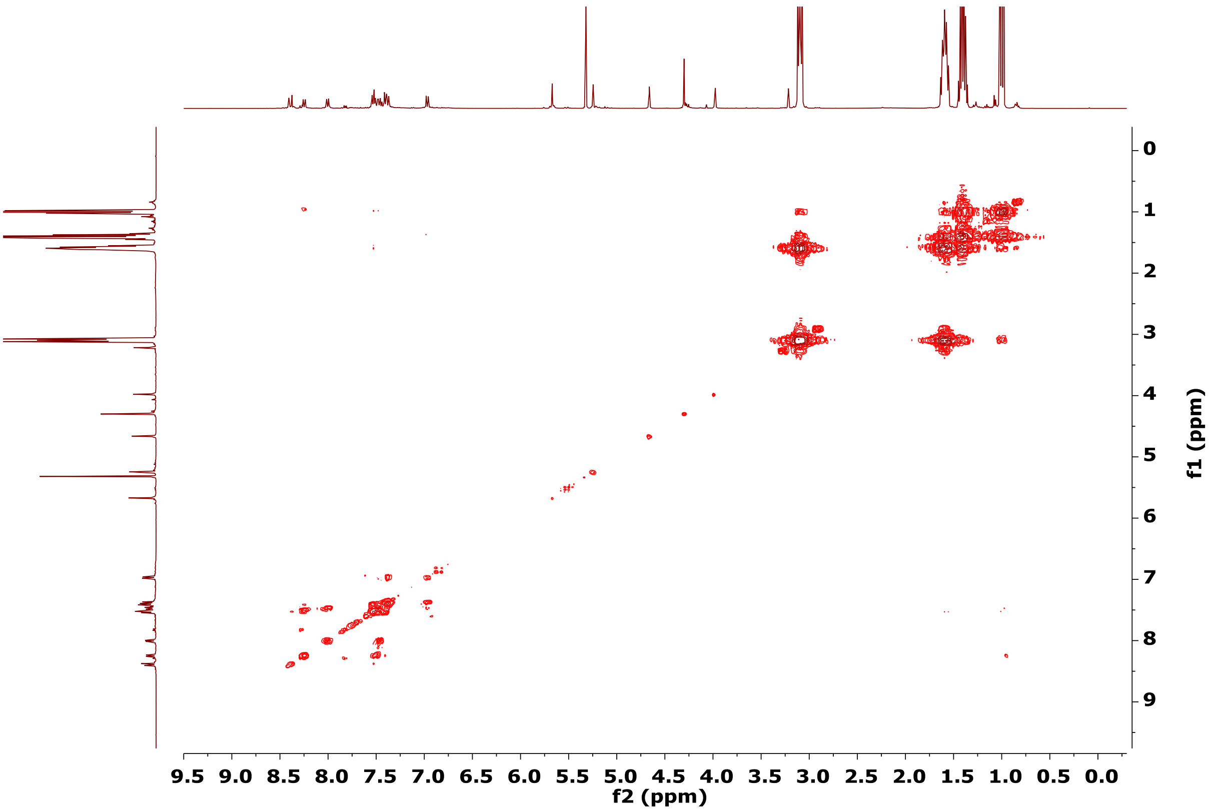


**Figure S36.** $^{1}H$-$^{1}H$ NMR COSY (400 MHz/400 MHz, $CD_2Cl_2$, 298 K) spectra of **$2^{+}$**.

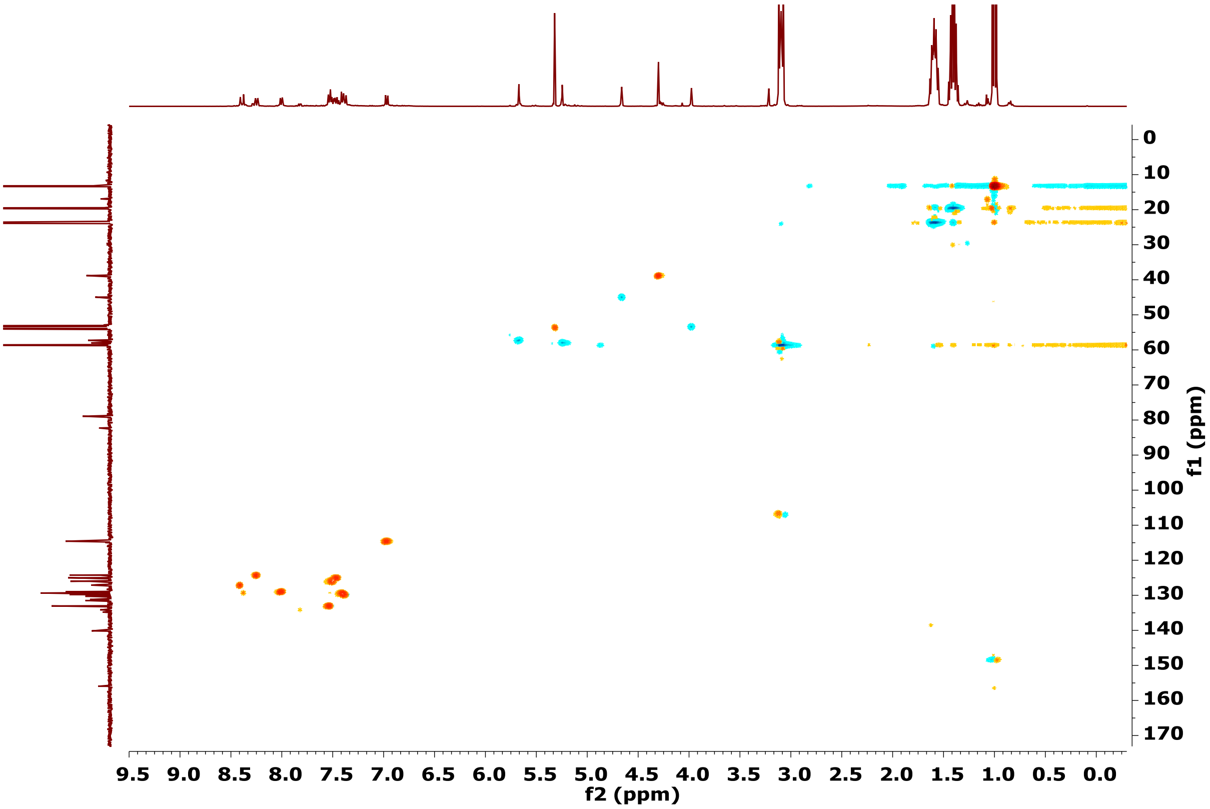


**Figure S37**. $^{1}H$-$^{13}C$ HSQC (400 MHz/101 MHz, $CD_2Cl_2$, 298 K) spectra of **$2^{+}$**.

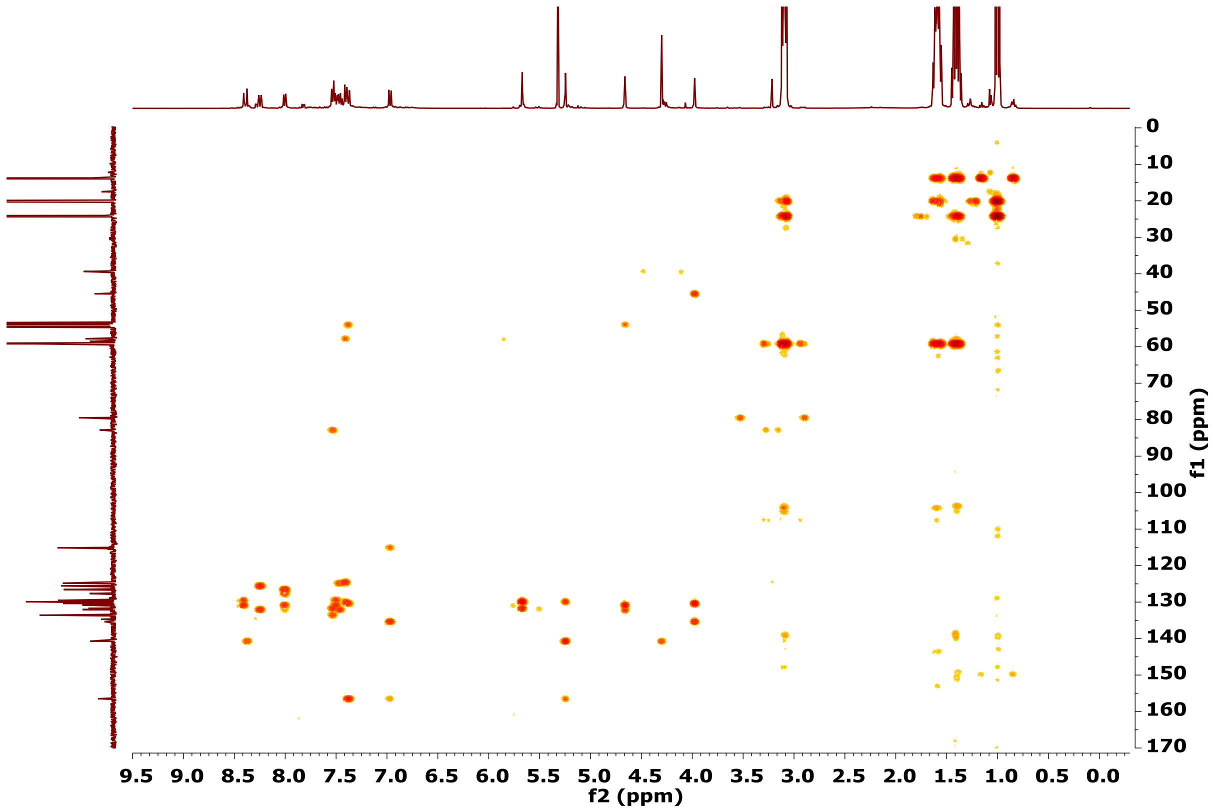


**Figure S38**. $^{1}H$-$^{13}C$ HMBC (400 MHz/101 MHz, $CD_2Cl_2$, 298 K) spectra of **$2^+$.**

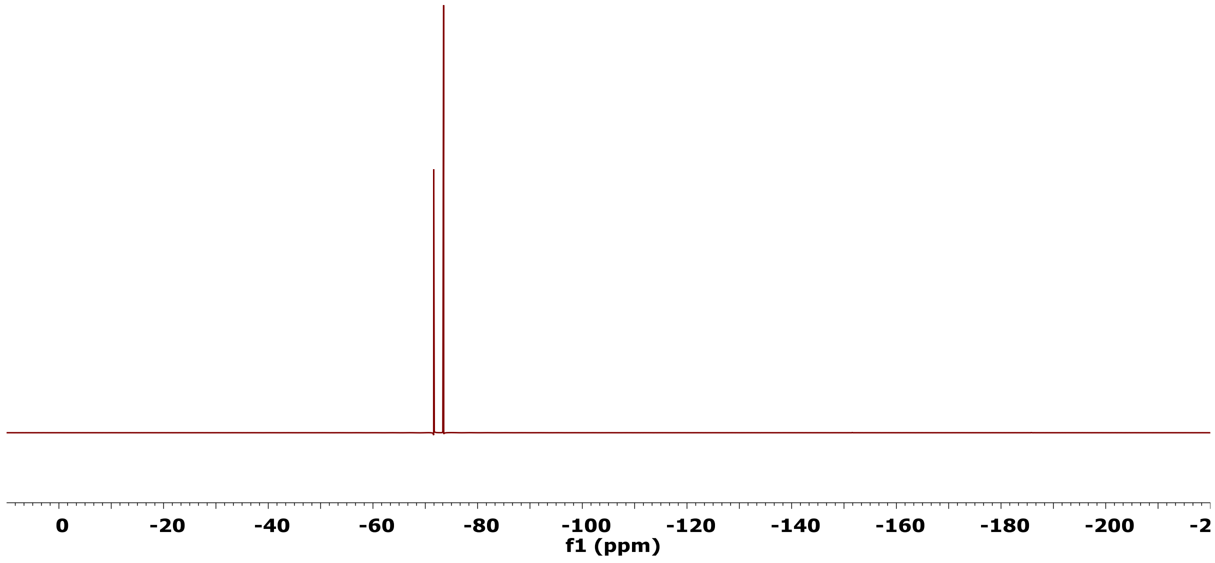


**Figure S39**. $^{19}F$ NMR (376 MHz, $CD_2Cl_2$, 298 K) specra of **$2^+$**.

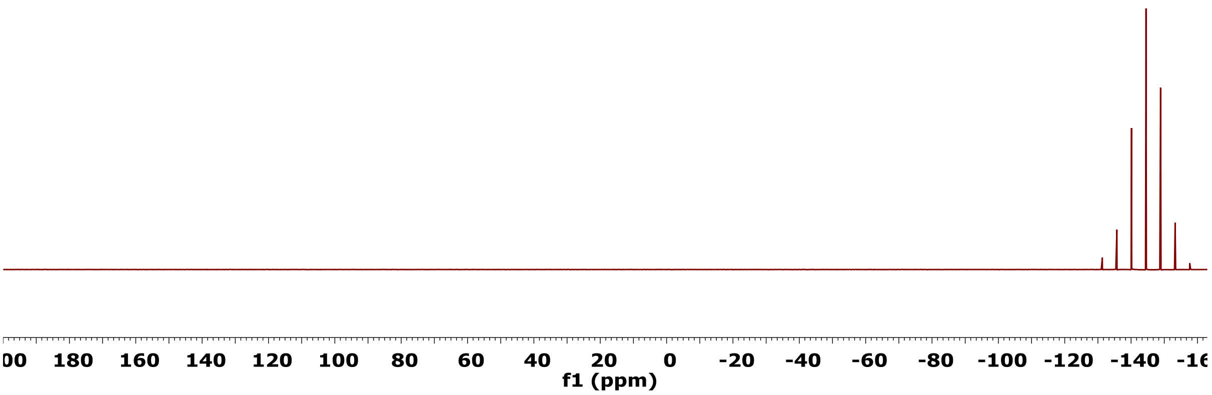


**Figure S40**. $^{31}$P NMR (162 MHz, $CD_2Cl_2$, 298 K) spectra of **2**$^+$.

## 7. IR Spectra

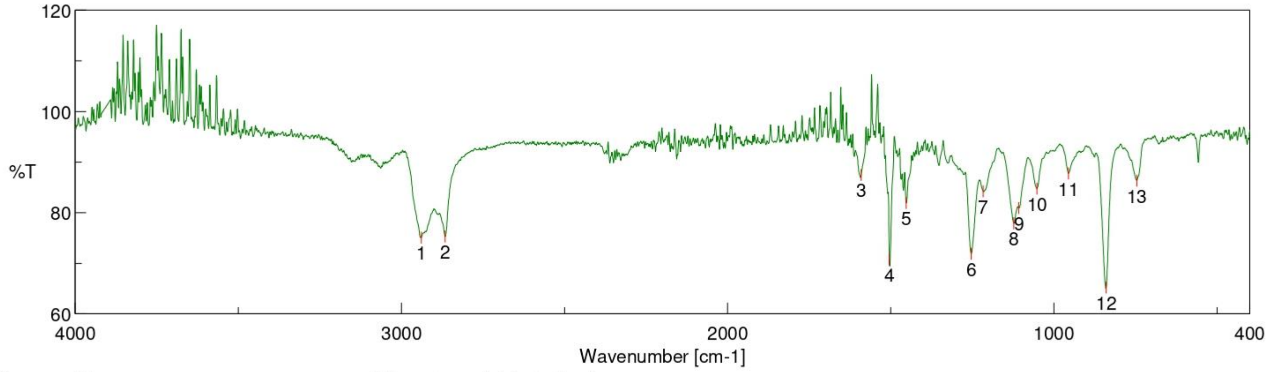


| | | | |
|---|---|---|---|
| [Comments] | | [Measurement Information] | |
| Sample name | skn96_5 | Model Name | FT/IR-4600typeA |
| Comment | dcm | Serial Number | F179361786 |
| User | sohom | | |
| Division | | Measurement Date | 25.02.2025 17:41 |
| Company | Uni Essen | | |
| | | Light Source | Standard |
| [Detailed Information] | | Detector | TGS |
| Creation date | 25.02.2025 17:42 | Accumulation | 64 |
| | | Resolution | 2 cm-1 |
| Data array type | Linear data array | Zero Filling | On |
| Horizontal axis | Wavenumber [cm-1] | Apodization | Cosine |
| Vertical axis | %T | Gain | Auto (32) |
| Start | 399.675 cm-1 | Aperture | Auto (5 mm) |
| End | 4000.12 cm-1 | Scanning Speed | Auto (2 mm/sec) |
| Data interval | 0.482117 cm-1 | Filter | Auto (30000 Hz) |
| Data points | 7469 | | |

**Figure S41.** IR (ATR-FT) spectra of **R1a-H**$^+$

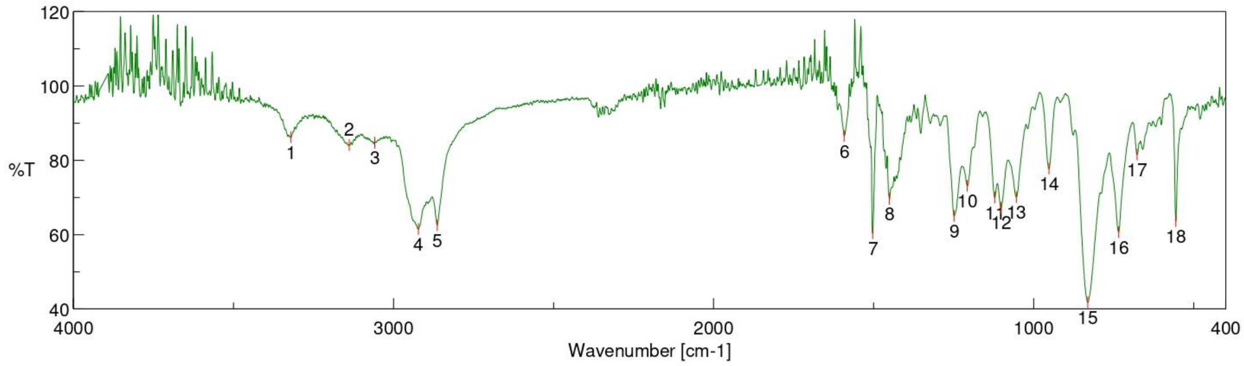


| | | | |
|---|---|---|---|
| [Comments] | | [Measurement Information] | |
| Sample name | skn88 | Model Name | FT/IR-4600typeA |
| Comment | solid | Serial Number | F179361786 |
| User | sohom | | |
| Division | | Measurement Date | 25.02.2025 17:47 |
| Company | Uni Essen | | |
| | | Light Source | Standard |
| [Detailed Information] | | Detector | TGS |
| Creation date | 25.02.2025 17:48 | Accumulation | 64 |
| | | Resolution | 2 cm-1 |
| Data array type | Linear data array | Zero Filling | On |
| Horizontal axis | Wavenumber [cm-1] | Apodization | Cosine |
| Vertical axis | %T | Gain | Auto (32) |
| Start | 399.675 cm-1 | Aperture | Auto (5 mm) |
| End | 4000.12 cm-1 | Scanning Speed | Auto (2 mm/sec) |
| Data interval | 0.482117 cm-1 | Filter | Auto (30000 Hz) |
| Data points | 7469 | | |

**Figure S42.** IR (ATR-FT) spectra of **R1-H$^{2+}$**

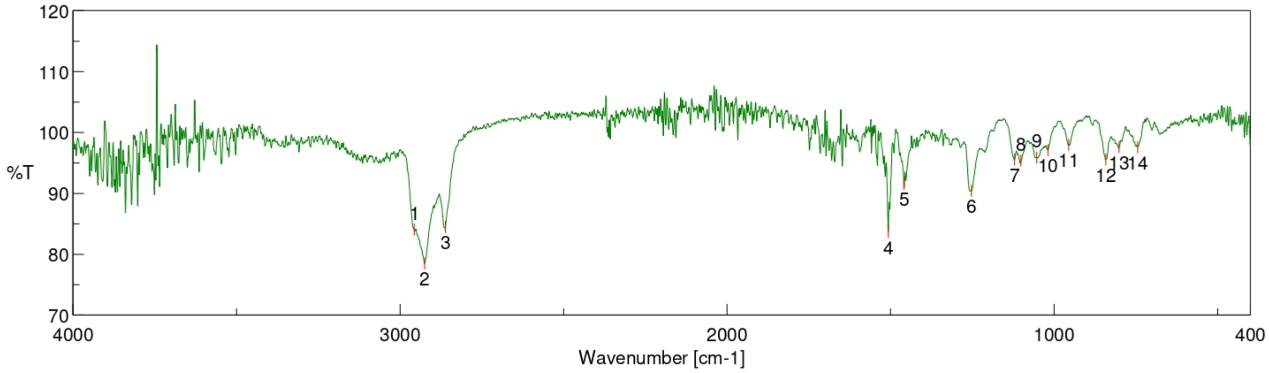


| | | | |
|---|---|---|---|
| [Comments] | | [Measurement Information] | |
| Sample name | skn92-1_5 | Model Name | FT/IR-4600typeA |
| Comment | dcm solution | Serial Number | F179361786 |
| User | sohom | | |
| Division | | Measurement Date | 25.02.2025 17:05 |
| Company | Uni Essen | | |
| | | Light Source | Standard |
| [Detailed Information] | | Detector | TGS |
| Creation date | 25.02.2025 17:08 | Accumulation | 32 |
| | | Resolution | 2 cm-1 |
| Data array type | Linear data array | Zero Filling | On |
| Horizontal axis | Wavenumber [cm-1] | Apodization | Cosine |
| Vertical axis | %T | Gain | Auto (32) |
| Start | 399.675 cm-1 | Aperture | Auto (5 mm) |
| End | 4000.12 cm-1 | Scanning Speed | Auto (2 mm/sec) |
| Data interval | 0.482117 cm-1 | Filter | Auto (30000 Hz) |
| Data points | 7469 | | |

**Figure S43.** IR (ATR-FT) spectra of **R2-H$^{2+}$**

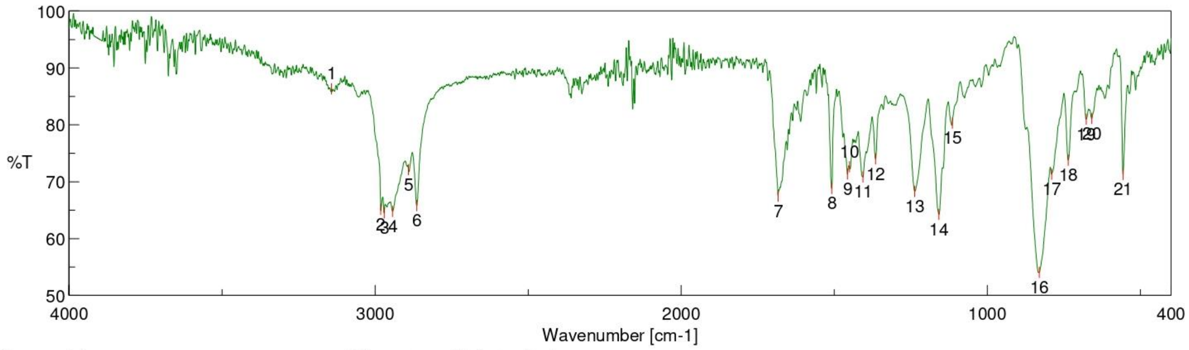


| [Comments] | | [Measurement Information] | |
|---|---|---|---|
| Sample name | jr721-1 | Model Name | FT/IR-4600typeA |
| Comment | solid | Serial Number | F179361786 |
| User | Jan Riebe | | |
| Division | ak niemeyer | Measurement Date | 09.03.2025 17:57 |
| Company | Uni Essen | | |
| | | Light Source | Standard |
| [Detailed Information] | | Detector | TGS |
| Creation date | 09.03.2025 17:58 | Accumulation | 32 |
| | | Resolution | 2 cm-1 |
| Data array type | Linear data array | Zero Filling | On |
| Horizontal axis | Wavenumber [cm-1] | Apodization | Cosine |
| Vertical axis | %T | Gain | Auto (32) |
| Start | 399.675 cm-1 | Aperture | Auto (5 mm) |
| End | 4000.12 cm-1 | Scanning Speed | Auto (2 mm/sec) |
| Data interval | 0.482117 cm-1 | Filter | Auto (30000 Hz) |
| Data points | 7469 | | |

[ Result of Peak Picking ]

| No. | Position | Intensity | No. | Position | Intensity | No. | Position | Intensity |
|---|---|---|---|---|---|---|---|---|
| 1 | 3142.44 | 86.2925 | 2 | 2981.41 | 65.1329 | 3 | 2970.32 | 64.5162 |
| 4 | 2942.84 | 64.7527 | 5 | 2890.77 | 72.1081 | 6 | 2863.77 | 65.7652 |
| 7 | 1683.55 | 67.4324 | 8 | 1508.06 | 68.7901 | 9 | 1456.96 | 71.3175 |
| 10 | 1450.21 | 72.6061 | 11 | 1406.33 | 70.8242 | 12 | 1365.35 | 73.9754 |
| 13 | 1237.11 | 68.2695 | 14 | 1158.04 | 64.2508 | 15 | 1114.65 | 80.2587 |
| 16 | 830.687 | 54.0175 | 17 | 789.707 | 71.298 | 18 | 735.71 | 73.7542 |
| 19 | 677.374 | 80.9486 | 20 | 658.571 | 81.1025 | 21 | 556.845 | 71.2542 |

**Figure S44.** IR (ATR-FT) spectra of **S1**$^{+}$

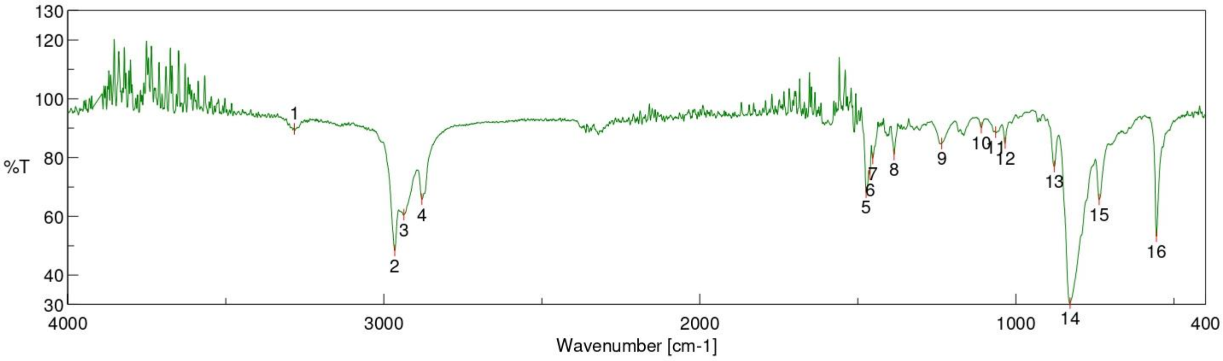


| [Comments] | | [Measurement Information] | |
|---|---|---|---|
| Sample name | jr729-1 | Model Name | FT/IR-4600typeA |
| Comment | solid | Serial Number | F179361786 |
| User | jr | | |
| Division | | Measurement Date | 25.02.2025 18:01 |
| Company | Uni Essen | | |
| | | Light Source | Standard |
| [Detailed Information] | | Detector | TGS |
| Creation date | 25.02.2025 18:01 | Accumulation | 64 |
| | | Resolution | 2 cm-1 |
| Data array type | Linear data array | Zero Filling | On |
| Horizontal axis | Wavenumber [cm-1] | Apodization | Cosine |
| Vertical axis | %T | Gain | Auto (32) |
| Start | 399.675 cm-1 | Aperture | Auto (5 mm) |
| End | 4000.12 cm-1 | Scanning Speed | Auto (2 mm/sec) |
| Data interval | 0.482117 cm-1 | Filter | Auto (30000 Hz) |
| Data points | 7469 | | |

**Figure S45.** IR (ATR-FT) spectra of **2**$^{+}$

## 8. HR-MS Spectra

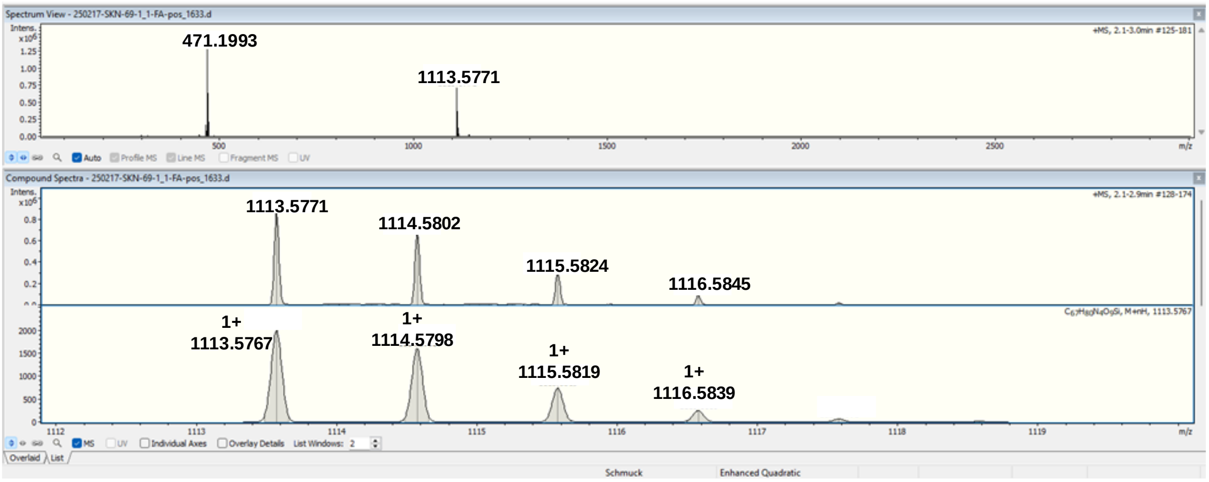


**Figure S46.** HR-MS spectra of [**R1a-H**]$^{+}$. *m/z* = 471.1993 represents [**1-**Na]$^{+}$ .

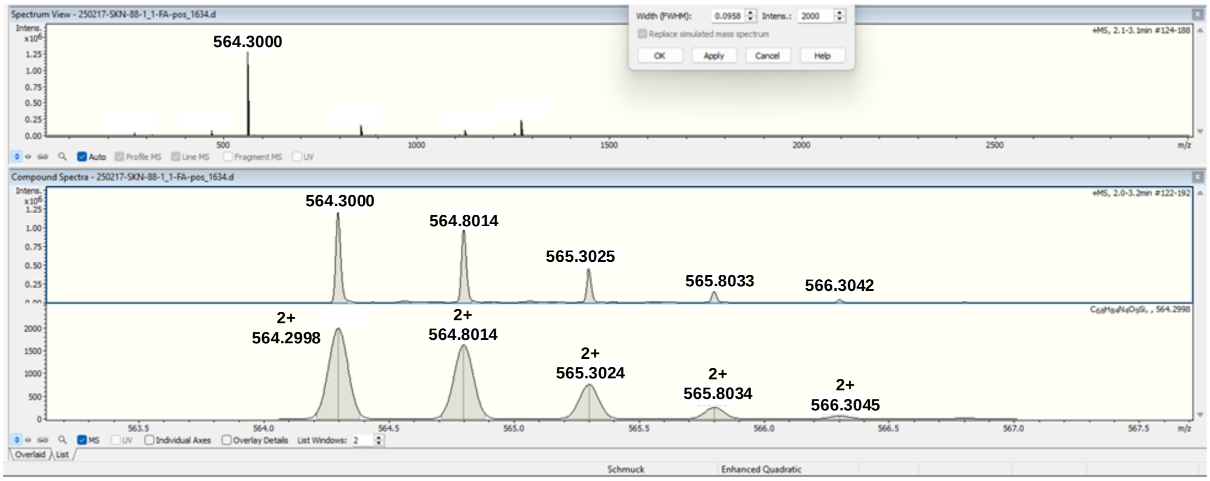


**Figure S47.** HR-MS spectra of [**R1-H**]$^{2+}$.

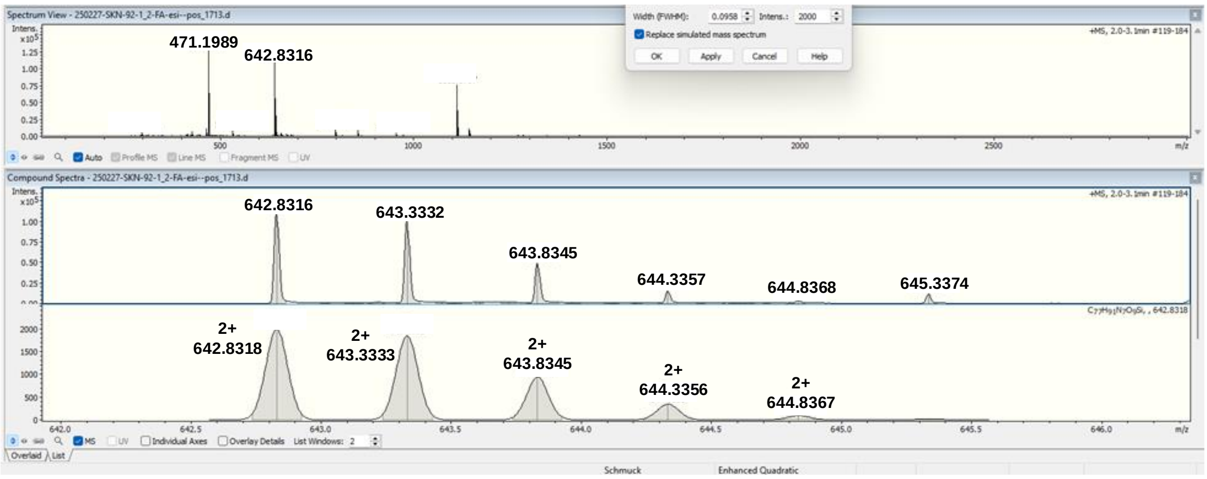


**Figure S48.** HR-MS spectra of [**R2-H**]$^{2+}$. $m/z$ = 471.1993 represents [**1**-Na]$^{+}$

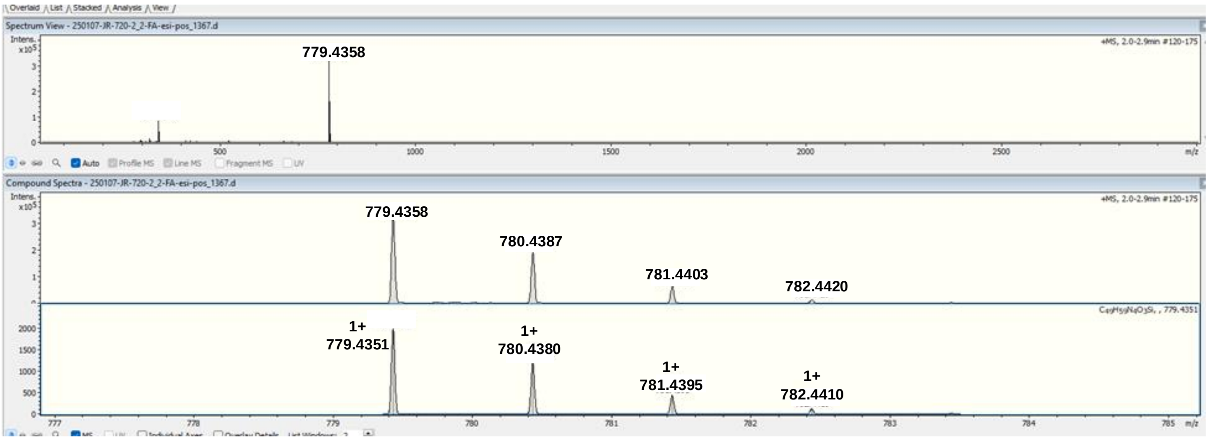


**Figure S49.** HR-MS spectra of [**S1**]$^{+}$.

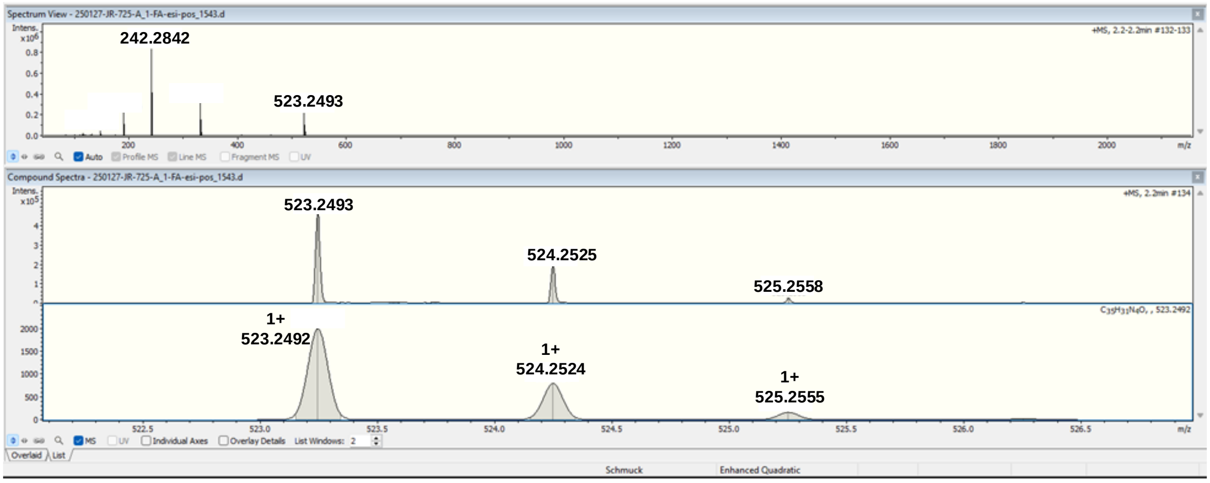


**Figure S50.** HR-MS spectra of [**2**]$^{+}$. $m/z$ = 242.2842 represents residual [$n$-$Bu_4$]$^{+}$.

## 9. References


[1] M. Betou, N. Kerisit, E. Meledje, Y. R. Leroux, C. Katan, J. F. Halet, J. C. Guillemin, Y. Trolez, *Chem. Eur. J.* **2014**, *20*, 9553-9557.

[2] A. Ghosh, I. Paul, M. Schmittel, *J. Am. Chem. Soc.* **2021**, *143*, 5319-5323.
